# Multiscale Coupling of One-dimensional Vascular Models and Elastic Tissues

**DOI:** 10.1007/s10439-021-02804-0

**Published:** 2021-07-19

**Authors:** Luca Heltai, Alfonso Caiazzo, Lucas O. Müller

**Affiliations:** 1 International School for Advanced Studies (SISSA), Trieste, Italy; 2 Weierstrass Institute for Applied Analysis and Stochastics (WIAS) Berlin, Mohrenstrasse 39, 10117 Berlin, Germany; 3 University of Trento, Via Sommarive 14, 38123 Povo, Italy

**Keywords:** Vascularized tissues, Immersed methods, Finite element methods, Finite volume methods

## Abstract

We present a computational multiscale model for the efficient simulation of vascularized tissues, composed of an elastic three-dimensional matrix and a vascular network. The effect of blood vessel pressure on the elastic tissue is surrogated via hyper-singular forcing terms in the elasticity equations, which depend on the fluid pressure. In turn, the blood flow in vessels is treated as a one-dimensional network. Intravascular pressure and velocity are simulated using a high-order finite volume scheme, while the elasticity equations for the tissue are solved using a finite element method. This work addresses the feasibility and the potential of the proposed coupled multiscale model. In particular, we assess whether the multiscale model is able to reproduce the tissue response at the effective scale (of the order of millimeters) while modeling the vasculature at the microscale. We validate the multiscale method against a full scale (three-dimensional) model, where the fluid/tissue interface is fully discretized and treated as a Neumann boundary for the elasticity equation. Next, we present simulation results obtained with the proposed approach in a realistic scenario, demonstrating that the method can robustly and efficiently handle the one-way coupling between complex fluid microstructures and the elastic matrix.

**Supplementary Information:**

The online version contains supplementary material available at 10.1007/s10439-021-02804-0.

## Introduction

The mechanics of vascularized tissues involves processes happening on a wide range of spatial scales, as well as the intrinsic coupling of solid and fluid phases. Developing efficient and high fidelity computational models to understand these processes is extremely important in order to characterize biomarkers for tissue diseases and to support non-invasive diagnosis based on medical imaging.

This work is motivated by modern medical imaging protocols based on Magnetic Resonance Elastography (MRE), a quantitative imaging technique sensitive to the mechanical properties of living tissues. By combining mechanical excitations at moderate frequencies with phase-contrast MRI, MRE allows to acquire the *internal displacement* field within a tissue sample. This information, combined with a mechanical model, allows to retrieve, non invasively and *in vivo*, information about the elastic parameters of the tissue.

Due to the limited resolution—typically order of millimeters—MRE allows to reconstruct properties at the *effective* tissue scale. The models currently used in MRE therefore mostly describe tissues as linear (visco-)elastic materials, for applications in the context of the diagnosis and monitoring of diseases such as cancer and fibrosis, that are characterized by different tissue stiffnesses.^11^,^24^,^27^,^31^

However, in several clinical applications, the intrinsic properties of vascularized tissues and the role of the interstitial fluid cannot be neglected. The pioneer experimental protocol recently proposed in,^14^ aimed at estimating the contribution of fluid components in the brain MRE, demonstrated the importance of taking into account the fluid phase. From a computational point of view, fully resolved biphasic models, i.e., accounting for the coupling between the tissue and the fluid vasculature at the microscale, are practically unfeasible. With the purpose of enhancing the quality and the outreach of MRE analysis, this paper addresses the issues of developing a computational multiscale model that can account for an arbitrary complexity of the (microscale) fluid vasculature and, at the same time, be efficiently upscaled for the application in the context of (coarse) MRE data. To this aim, we extend the immersed multiscale framework recently proposed in,^9^,^10^ based on describing the tissue as an elastic material, taking into account the presence of the fluid network as a singular forcing term. In particular, we address the coupling between the three-dimensional elastic matrix and a finite volume one-dimensional blood flow model that can efficiently handle complex vasculature networks. The elasticity problem is solved using a finite element method on hexahedral meshes, implemented in the open source library deal.II.^2^,^3^ The blood flow within the 1D network is computed using a high order finite volume method (see, e.g. 17), which has already been used in several contexts (see, e.g., 18,19).

The main goal of this work is to demonstrate the potential of the multiscale approach for the *in silico* simulation of vascular tissues. We show numerical results in two different examples. Firstly, we compare the multiscale method against a fully resolved 3D simulation for the case of a bifurcation immersed in a tissue sample. Next, we employ the immersed method to simulate—at the microscale—a vascular network of about 1900 vessels immersed in a tissue voxel of the order of few millimeters. In particular, we analyze the mechanical response of the tissue at the effective scale, i.e., the one typically observed in the context of medical imaging.

## Materials and Methods

### Model Setting

In order to introduce the mathematical model, let us consider a three-dimensional tissue sample

$$\Omega = \Omega ^{\text{tissue}} \cup {\mathcal {V}},$$

containing an elastic matrix, denoted by an elastic matrix $\Omega ^{\text{tissue}}$ and a vasculature ${\mathcal {V}}$, assumed to be a connected set of thin vessels. We also denote with $\Gamma$ the common interface between the tissue and vasculature subdomains.

In what follows, we decompose the vascular network in a set of non-intersecting vessel segments. We further assume that each one of these vessel segments can be approximated with a cylindrical domain, described *via* a one-dimensional manifold, for which it is possible to introduce a one-dimensional arc-length curve

$$\gamma (s): [0,L] \rightarrow \Omega \subset R^3,$$

describing the vessel *centerline*, and a positive function

$$a(s): [0,L] \rightarrow R,$$

denoting the *radius* of the vessel cross-section for each $s \in [0,L]$.

We will assume that the curvature of each vessel, i.e., within a single segment, varies slowly w.r.t. to its arc length, and that the elastic stiffness of the vessels is comparable to that of the surrounding elastic matrix. For a possible way to model separately the elastic behaviour of the vessels we refer to.^1^

**Figure 1:**
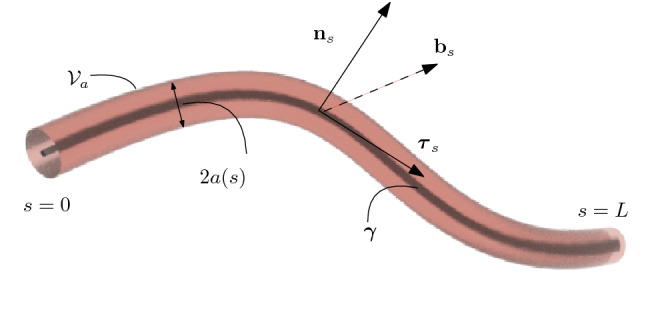
Sketch of a three-dimensional thin vessel, identified *via* its cross-sectional radius and its centerline.

Let us denote with *A*(*s*) the cross-section, i.e., the disk of radius *a*(*s*) orthogonal to $\gamma (s)$, and with $|A(s)| = \pi a^2(s)$ the cross-sectional area, for all $s \in [0,L]$ (see Fig. 1).

In order to formally derive the multiscale model, we introduce at each $s \in [0,L]$ also the *Frenet* frame $\varvec{\tau }_s =\gamma ^{\prime }(s)$ (tangential vector in *s*), $\mathbf {n}_s=\varvec{\tau }_s^{\prime }/|\varvec{\tau }_s^{\prime }|, \mathbf {b}_s= \varvec{\tau }_s\times \mathbf {n}_s$ (basis of the normal plane in *s*). The set

1 $${\mathcal {V}}_a(\gamma ) = \{ \mathbf {x}\in \Omega \text { s.t. } \text {dist}(\mathbf {x},\gamma ) < a \},$$

denotes the thin vessel domain.

Assuming that the domain ${\mathcal {V}}_a(\gamma )$ describes a non-intersecting vessel segment, we introduce a local coordinate transformation mapping

2 $$\varvec{\varphi }(r, \theta , s) := \gamma (s) + r\cos (\theta ) \mathbf {n}_s +r\sin (\theta ) \mathbf {b}_s,$$

which is one-to-one from a cylindrical domain in polar coordinates $(r,\theta ,s) \in (0,a(s)] \times [0,2\pi ] \times [0,L]$ onto ${\mathcal {V}}_a(\gamma )$.

We denote with $\gamma ^{-1}: {\mathcal {V}}_a(\gamma ) \mapsto [0,L]$ the function that identifies, for each point $\mathbf {x}$ in ${\mathcal {V}}_a(\gamma )$, the arc-length coordinate $s \in [0,L]$ such that $\gamma (s)$ has minimum distance from $\mathbf {x}$, i.e.,

3 $$\gamma ^{-1}(\varvec{\varphi }(r,\theta ,s)) := s, \quad \forall r \in [0,a(s)], \quad \forall \theta \in [0,2\pi ].$$

In order to describe the hemodynamics in the vasculature, we finally introduce the one-dimensional blood pressure functions

$$p_{\gamma }(s) : [0,L] \rightarrow \Omega \subset R$$

and its extension

$$p_{\text{3D}}(\mathbf {x}) = p_{\gamma }\left( \gamma ^{-1}(\mathbf {x})\right) \,, \forall \mathbf {x}\in {\mathcal {V}}_a\,,$$

defined in the three dimensional vessel.

### Three-Dimensional Elastic Tissue Modeling

Let [0, *T*] be the considered time interval, and let us assume to be given a function $\overline{p}(t)$, $t \in [0,T]$, denoting the excess pressure field inside the vasculature, defined as the difference between the fluid pressure at time *t* and the initial pressure. The focus of the current work is to infer effective elastic properties of vascularized tissue samples in the framework of elastography (MRE). In the regime of MRE measurements, we therefore initially assume the dynamics of the tissue to be described by a quasi-static linear elastic problem, even though the framework we present may be extended easily to non-linear viscoelastic materials. Further justification for the choice of linear models comes from the fact that the cross-section area of the vessels only varies of a few percent, resulting in a very small perturbation of the overall displacement of the elastic tissue, due to the relative size of the vessel radius w.r.t. to the size of the tissue sample. We assume that the displacement field $\mathbf {u}^{\text{3D}}_t:\Omega ^{\text{tissue}} \rightarrow {\mathbb {R}}^3$ at each time $t \in [0,T]$ obeys the partial differential equation

4 $$\begin{aligned}&-\nabla \cdot \underline{\varvec{\sigma }}(\mathbf {u}^{\text{3D}}_t) = \varvec{0},\quad \;\text { in }\;\Omega ^{\text{tissue}}, \\&\mathbf {u}^{\text{3D}}_t = \varvec{0},\quad \text { on }\; \Gamma _D \,, \\&\underline{\varvec{\sigma }}(\mathbf {u}^{\text{3D}}_t) \cdot \mathbf {n}= \varvec{0},\quad \text { on }\; \Gamma _N\,, \\&\underline{\varvec{\sigma }}(\mathbf {u}^{\text{3D}}_t) \cdot \mathbf {n}=-\overline{p}(t) \, \mathbf {n}\quad \text { on }\; \Gamma \,. \end{aligned}$$

In (4),

5 $$\underline{\varvec{\sigma }}(\mathbf {u}) := 2 \mu \underline{\varvec{e}}(\mathbf {u}) + \lambda I \varvec{\nabla }\cdot \mathbf {u}$$

stands for the Cauchy stress tensor, $\underline{\varvec{e}}(\mathbf {u}) =\frac{1}{2}( \nabla \mathbf {u}+ \nabla \mathbf {u}^T)$ denotes the symmetric part of the infinitesimal strain tensor, $\mu$ and $\lambda$ are the so called Lamé constants, and *I* is the identity matrix. Moreover, $\Gamma _D$ (resp. $\Gamma _N$) is the subset of $\partial \Omega ^{\text{tissue}}$ where displacement (resp. external forces) are imposed as boundary conditions.

#### Remark 1

In the quasi-static approximation of the elasticity problem, the time dependency is only given by the the variation over time of the boundary condition (4)$_3$.

Let us now introduce the functional spaces

6 $$\mathbf {V} := \{ \mathbf {v}\in (H^1(\Omega ^{\text{tissue}}))^d, \text { such that } \mathbf {v}|_{\Gamma _D} = \varvec{0} \},$$

and let us denote, for a general $\Omega ^a \subset {\mathbb {R}}^d$, with $(\cdot , \cdot )_{\Omega ^a}$ the inner product in $(L^2(\Omega ^a))^2$.

Multiplying (4) with $\mathbf {v}\in \mathbf {V}$ and integrating by parts yields a standard variational formulation of problem (4): Find the displacement $\mathbf {u}^{\text{3D}}_t \in \mathbf {V}$ solution to:

7 $$\begin{aligned}&2\mu (\underline{\varvec{e}}(\mathbf {u}^{\text{3D}}_t), \underline{\varvec{e}}(\mathbf {v}))_{\Omega ^{\text{tissue}}} + \lambda (\varvec{\nabla }\cdot \mathbf {u}^{\text{3D}}_t, \varvec{\nabla }\cdot \mathbf {v})_{\Omega ^{\text{tissue}}} \\&\quad = \int _{\Gamma } \bar{p}(t) \mathbf {n}\cdot \mathbf {v}\text{d} \Gamma \quad \forall \mathbf {v}\in \mathbf {V}\,. \end{aligned}$$

We aim to approximate the solution of the elasticity problem (4) on the domain $\Omega _a$ by constructing a variational formulation on the whole domain $\Omega$. To this aim, we extend the original problem with a fictitious problem in ${\mathcal {V}}$. As described in,^9^ we seek for the solution of a problem of the following form:

let $p_{\gamma }$ denote the one-dimensional excess pressure field defined on ${\mathcal {V}}$, and let be given the vessel centerline $\gamma$, and the radius function *a* (as defined in Section 2.1). For the sake of simplicity, we remove the subscript referring to time dependency in the following derivation.

At each $t \in [0,T]$, we then seek $\mathbf {u}\in \mathbf {V}$ such that

$$\begin{aligned}&(2\mu \underline{\varvec{e}}(\mathbf {u}), \underline{\varvec{e}}(\mathbf {v}) )_{\Omega } + (\lambda \varvec{\nabla }\cdot \mathbf {u}, \varvec{\nabla }\cdot \mathbf {v})_{\Omega }\\&\quad = <\mathbf {F}_{(\gamma , p_{\gamma }, a)},\mathbf {v}>\quad \forall \mathbf {v}\in \mathbf {V}. \end{aligned}$$

The singular source term $\mathbf {F}_{(\gamma , p_{\gamma },a)}$ is defined in such a way to enforce, for each $s \in [0,L]$, the correct value of the normal stresses across $\Gamma \cap A(s)$ depending on the fluid pressure on the vessel boundary $\Gamma$. For the detailed derivation of the forcing term, we refer to,^9^ which is briefly summarized below.

Let $\varvec{\tau }$ denote the vector tangential to the vessel centerline, and let us introduce the gradient operator in the plane orthogonal to $\varvec{\tau }$ as

8 $$\nabla _{\varvec{\tau }} \mathbf {u}:= \left( \underline{\varvec{1}} - \varvec{\tau }\otimes \varvec{\tau }\right) \nabla \mathbf {u}$$

and the planar divergences as

9 $$\nabla _{\varvec{\tau }} \cdot \mathbf {u}:= \text {tr} \left( \nabla _{\varvec{\tau }} \mathbf {u}\right) = \nabla \cdot \mathbf {u}- \varvec{\tau }\cdot \left( \nabla \mathbf {u}\, \varvec{\tau }\right) \,.$$

We consider a singular force of the form

10 $$\mathbf {F}_{(\gamma , p_{\gamma }, a)} = \mathbf {F}_{(\gamma , p_{\gamma },a)}^{H} +\mathbf {F}_{(\gamma , p_{\gamma },a)}^{\varvec{\tau }}$$

composed of a hyper-singular term

11 $$\begin{aligned}&\mathbf {F}_{(\gamma , p_{\gamma },a)}^{H}(\mathbf {x}) \\&\quad := \int _0^L \frac{2\mu +\lambda }{\mu }A(s) p_{\gamma }(s) \varvec{\nabla }_{\varvec{\tau }} \delta (\mathbf {x}-\gamma (s)) \text{d} s,\; \quad \forall \mathbf {x}\in \Omega \, \end{aligned}$$

and a singular source

12 $$\begin{aligned}&\mathbf {F}_{(\gamma , p_{\gamma },a)}^{\varvec{\tau }}(\mathbf {x}) \\&\quad = \int _0^L \frac{2\mu +\lambda }{\mu }(A(s) p_{\gamma }(s))' \, \delta (\mathbf {x}-\gamma (s)) \varvec{\tau }\text{d} s ,\; \quad \forall \mathbf {x}\in \Omega \,. \end{aligned}$$

The term defined in (12) has support on the centerline and it is directed tangential to it. In particular, if vessel radius and pressure vary slowly along $\gamma$, the singular term (12) can be neglected, and the immersed method reduces to a hypersingular force equal to the tangential derivative of a Dirac delta distribution.

The two source terms (11) and (12) have the effect of imposing a solution which is close to the one that would be obtained with a full domain discretization, provided that the cross-sectional area of the vessels is negligible w.r.t. to the diameter of the sample domain $\Omega$. As detailed in,^9^ this is achieved by computing the singular source terms that would be required to solve the problem exactly on a large domain with a single embedded vessel. A similar strategy is possible in the non-linear case, provided that one resorts to Lagrange multipliers, as in.^1^

Notice that the forces introduced in (11)–(12) depend only on one-dimensional information, such as centerline, the excess pressure $p_{\gamma }(s)$, the radius, and the cross-sectional area, and it allows therefore to represent the vessel uniquely through a one-dimensional manifold.

Moreover, it is important to observe that the definition of the hypersingular line sources (i.e., the line where the singular sources are applied) is independent of the discretization used for the solution of the one-dimensional flow. In fact, the coupling with the one-dimensional flow solver depends only on the values of the one-dimensional pressure and area, interpolated on the hypersingular line (see Section 2.4).

Since the hypersingular source term is not in $V^*$, we resort to a regularized strategy^10^ which allows one to replace the Dirac delta distribution of the source terms in (11) and (12) with a smooth approximation with compact support of radius $\varepsilon$. The analysis in^10^ suggests that the support of the approximated Dirac distribution should be chosen proportional to the grid size *h*, in order to obtain the best results in terms of convergence rates and computational costs.

In particular, we use a tensor product $C^1$ approximation of the Dirac delta distribution, defined through the generating function

13 $$\psi (s) := \chi _{[-1,1]}(s) \frac{1}{2} (1+\cos (\pi s)),$$

where $\chi _{[-1,1]}$ is one inside the interval $[-1,1]$, and zero elsewhere.

A possible one-parameter approximation of the Dirac delta distribution is then given by

14 $$\delta ^\varepsilon (x) := \frac{1}{\varepsilon ^3} \prod _{i=1}^3 \psi \left( \frac{1}{\varepsilon }x_i\right) ,$$

and we have that

15 $$\lim _{\varepsilon \rightarrow 0} \delta ^\varepsilon (x) = \delta (x).$$

In the simulations, we set $\varepsilon = h$. The right hand side of the elasticity problem may then be computed (taking the hyper singuar term as an example) by

16 $$\begin{aligned} <\mathbf {F}^H_{(\gamma , p_{\gamma }, a)},\mathbf {v}_i>&\simeq \sum _{x_q} \sum _{s_q} \frac{2\mu +\lambda }{\mu } \\&\quad A(s_q) p_{\gamma }(s_q) \nabla _{\varvec{\tau }} \delta ^\varepsilon (x_q-\gamma (s_q)) \mathbf {v}_i(x_q) w_{x_q} w_{s_q}. \end{aligned}$$

We notice here that the approximation of (16) only requires the definition of integration points on the vessels and on the computational grids, and the identification of all cells of the grid that fall within $\varepsilon$ distance from the vessels. The deal.II library uses efficient r-tree algorithms to construct nearest-neighbour informations on quadrature points and ease the computation of (16).

### One-Dimensional Blood Flow Model

The main advantage of 1D hemodynamic models is that they provide suitable approaches to investigate pressure and flow waveform in complex arterial and venous networks, while keeping the overall computational cost reasonably low. In the last decades, these approaches have been widely used for several applications (see, e.g., 5,12,13,20,25). Moreover, they have been deeply validated both versus *in vitro* (see, e.g., 16) and *in vivo* experiments (see, e.g., 6,19,26).

In order to introduce the model equation, we consider a network of interconnected impermeable vessels, denoting with *s* the one-dimensional coordinate along the segments. The blood flow is then described in terms of the vessel cross-sectional area *A*(*s*, *t*), the mass flow rate *q*(*s*, *t*) and the average blood pressure over the cross section *p*(*s*, *t*), according to mass conservation and momentum balance laws:

17 $$\begin{aligned} \left\{ \begin{array}{c} \dfrac{\partial A}{\partial t}+\dfrac{\partial q}{\partial s} = 0,\\ \dfrac{\partial q}{\partial t}+\dfrac{\partial }{\partial s} \left( \displaystyle \frac{q^2}{A} \right) +\dfrac{A}{\rho }\dfrac{\partial p}{\partial s} = -\dfrac{8\pi \eta }{\rho }\dfrac{q}{A}\,. \end{array}\right. \end{aligned}$$

In (17), $\eta$ stands for the dynamic viscosity of blood and $\rho$ denotes the blood density. These constants are set equal to $\eta =0.032\,P$ and $\rho =1.04\,g/cm^3$.

System (17) is closed introducing a constitutive law (so-called *tube law*) that accounts for the fluid-structure interaction between blood and vessel wall, relating the strain and strain rate of the vessel wall to the internal pressure.

We adopted a tube law of the form^5^:

18 $$\begin{aligned} p = \frac{\pi R_0 h_0}{A} \left[ E_e \varepsilon +E_c \epsilon _r \ln \left( e^{\frac{\varepsilon -\varepsilon _0}{\epsilon _r}}+1\right) \right] \,, \end{aligned}$$

where $R_0$, $A_0$, and $h_0$ are the vessel radius, the cross-sectional area and the wall thickness at reference state, respectively. Following,^5^ the vessel wall thickness is computed as

19 $$\begin{aligned} h_0 = R_0 \, \left( a e^{b\,R_0 + c e^{d\,R_0}}\right) \,, \end{aligned}$$

with $\text {a}=0.2802$, $\text {b}=-5.053\ \text {cm}^{-1}$, $c=0.1324$ and $d=-0.1114\ \text {cm}^{-1}$. The parameter $E_e=1.8\times 10^6\ \text {dyn}/\text {cm}^2$ stands for the effective Young modulus of elastin, while $E_c = 150\times 10^6\ \text {dyn}/\text {cm}^2$ is the effective Young modulus of collagen. Moreover, $\varepsilon _0 = 0.35$ is the deformation state for which $50\%$ of collagen fibers have been activated, $\epsilon _r=0.05$ is the standard deviation of the fiber activation state distribution and $\varepsilon = \sqrt{\frac{A}{A_0}}-1$ is the current deformation state.

Boundary conditions at the vessels’ ends can be of different nature. One can prescribe a certain variable, for example a pressure or flow rate waveform prescribed at the inlet of the network. Also, vessels can be coupled to other vessels via appropriate junction conditions. In this work, 1D vessels are coupled at junctions enforcing mass conservation and total pressure continuity, as well as using generalized Riemann invariants.^21^ In addition, at terminal sites, vessels can be coupled to lumped parameter models representing the peripheral circulation (see, e.g., 8).

The general solution of system (17)–(18) requires an efficient and robust numerical method, suitable to be used on large and small vessels and in the case of this work, on large vessel networks. We use a local time stepping finite volume numerical scheme,^22^, which has shown to possess the above mentioned features. This scheme is based on the ADER (Arbitrary high-order DERivative Riemann problem) methodology, which allows for arbitrary accuracy in space and time (see, e.g., 29,30).

### Coupling

For computing the one-dimensional hemodynamics, the blood vessel network is described by a finite set of interconnected impermeable segments. For the high-order finite volume method described in Section 2.3 each segment (vessel) is discretized as a single finite volume. In what follows, we will denote as $h_{1D,i}$ the size of the *i*-th vessel discretization in the 1D model, corresponding, in this case, to the vessel length. On the other hand, the three-dimensional tissue model is discretized using a structured hexahedra finite element mesh, whose element characteristic length will be denote with $h_{3D}$.

In order to technically implement the coupling between the one-dimensional hemodynamics and the three-dimensional elasticity problem, we first introduced an additional discretization of the vessel network, the size of which will be denoted as $h_{1D}^{3D}$. We consider the case $h_{1D}^{3D} < h_{1D,i}$. Then, for each vessel, we introduce additional internal points along the segments, with a spacing $h_{1D}^{3D}$,We refer to this set of points as the *discrete hypersingular points*. For each vessel *i*, we also introduce a function ${\mathcal {G}}_{1D,i}^{3D}$, defined on the one-dimensional coordinate of the vessel, mapping each discrete hypersingular point onto a point of the 3D domain, a direction (i.e., the tangential vector to the vessel *i*), and the values of cross-sectional area and pressure obtained from the 1D model:

20 $$\begin{aligned} {\mathcal {G}}_{1D,i}^{3D}: s \mapsto (\gamma _i(s),\varvec{\tau }_i,p_{\gamma }(s),A(s)) \in {\mathcal {V}}\times {\mathbb {R}}^3 \times {\mathbb {R}} \times {\mathbb {R}}\,. \end{aligned}$$

In this work, for computing pressure and cross-sectional areas, we considered a simple piecewise constant approximation over each vessel. Depending on the physical setting, i.e., on the variability of the results, higher order interpolations can also be considered.

At each time step of the solution of the one-dimensional model, the corresponding values obtained from (20) are then used in order to compute the hypersingular forcing term (11).

This approach is also used when solving a full 3D problem (4), in which the elastic tissue dynamics is equipped with Neumann boundary conditions on the surface of the vessel (considered as a full three-dimensional domain, see, e.g., Section 3.1). In this case, the one-dimensional pressures and areas need to be mapped onto the two-dimensional vessel surface and evaluated in order at selected (Gauss) points when computing the corresponding term in integral formulation. In order to compute the 3D extension, the values of the pressure $p_{\text{1D}}$ and areas $A_{\text{1D}}$ on the closest point of the one-dimensional network is used.

## Results

### Bifurcation

The goal of the first test is a comparison between the numerical results obtained with the proposed coupled multiscale method and a high-fidelity solution, obtained with a matching discretisation considering the full scale problem, where both tissue and vessel are discretized in three-dimensions. We observe that a comparison against experimental measurements is less relevant in this context, since it would require the resolution of a cascade of complex inverse problems to infer all the physical parameters that cannot be measured directly. Instead, the procedure we follow guarantees that we do not affect the overall accuracy when replacing a high fidelity model—only feasible in examples where the model parameters can be fully determined—with the multi-scale approach, i.e., that the error introduced by the multiscale formulation is of the same order of numerical discretisation errors.

**Figure 2:**
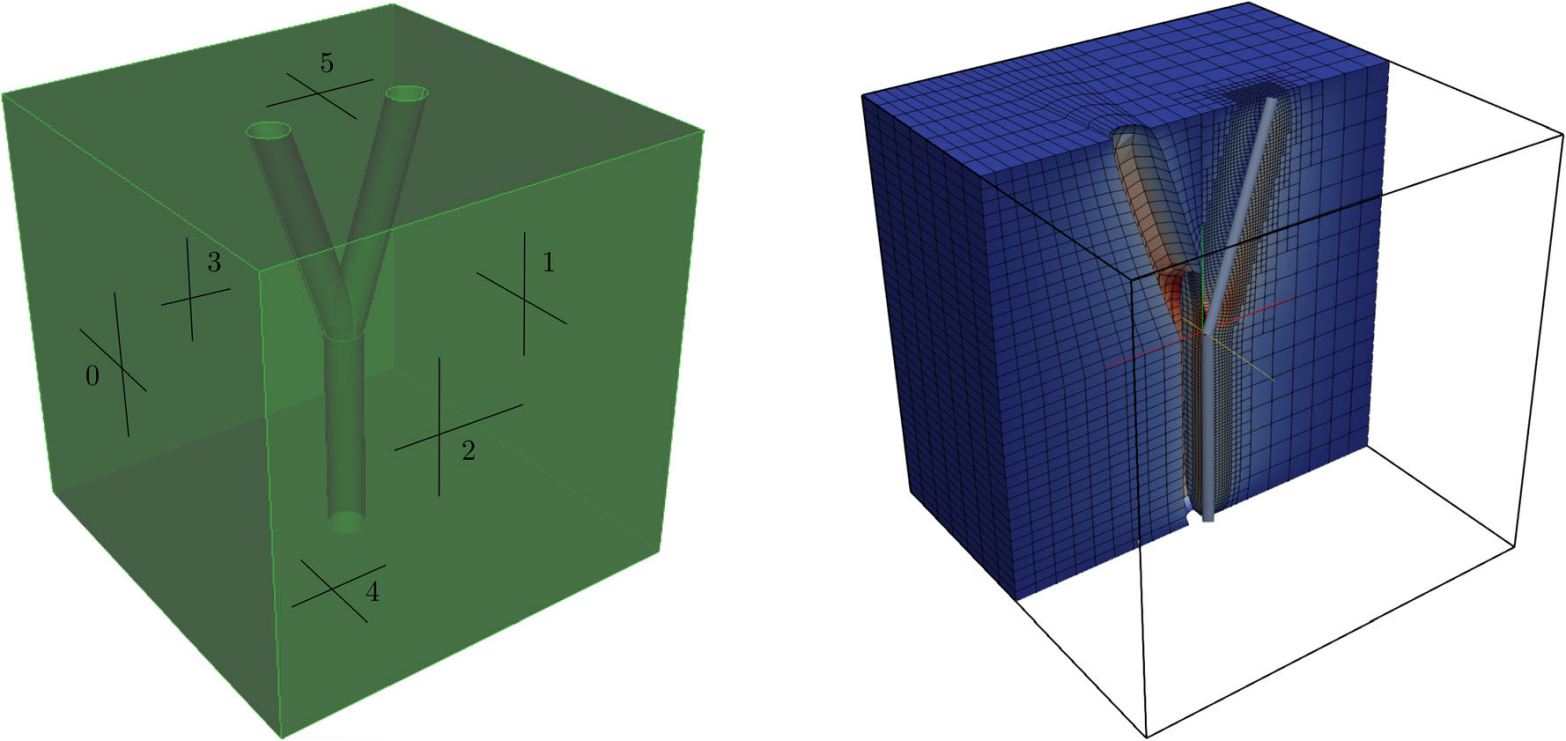
Left: The three-dimensional domain considered for the bifurcation model. The corresponding one-dimensional description is obtained considering vessel centerlines. Right: Comparison between the displacement obtained in the fully resolved 3D model (left part) and the result of the hypersingular model (right part). The right part of the figure shows also the one-dimensional vessel.

To this purpose, we consider first a simple bifurcation problem. Namely, the setup consists of a cubic tissue domain, of dimensions $2\times 2\times 2\ \text {mm}$, coupled to a bifurcation, composed of three vessel segments (see Fig. 2, left) with initial radius of 0.1 mm.

**Figure 3:**
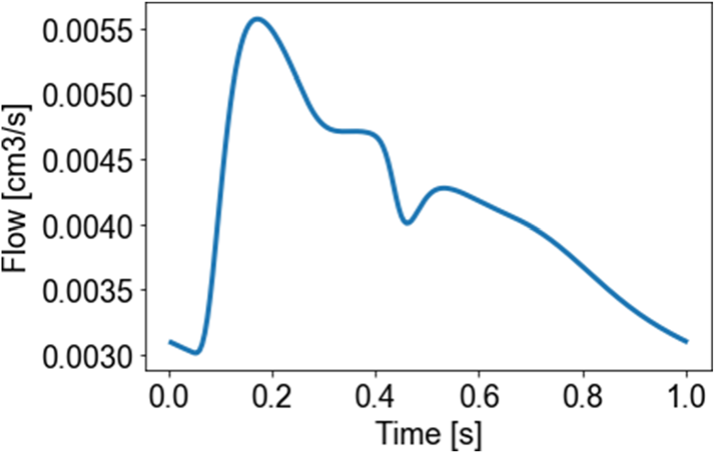
The flow rate imposed for solving the one-dimensional hemodynamics in the bifurcation setting.

For this problem, the solution of the one-dimensional model has been computed imposing a flow rate at the entrance of the bottom vessel (see Fig. 3) and using 3-elements Windkessel terminal models at the end of the top vessels. The resulting pressures and areas have then used to solve the coupled problem, both considering a full three-dimensional description and a reduced immersed method.

The full three-dimensional problem has been solved using a finite element method on hexahedral elements, using a uniform grid that resolves the interface between the fluid and the tissue domains. For the immersed 3D–1D method, the one dimensional bifurcation has been discretized using about 200 points along the each vessel centerline, and the elasticity problem has been solved using a hexahedral grid locally refined near the vessel centerline (see Fig. 2, right).

**Figure 4:**
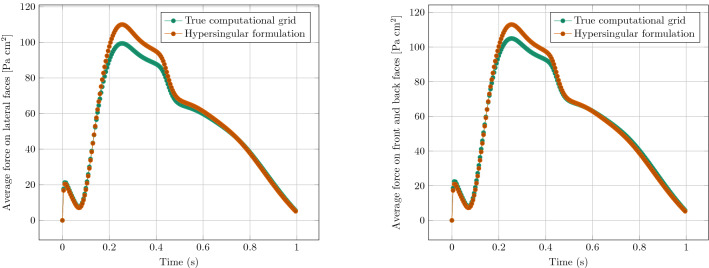
Comparison of the average forces measured on the lateral faces (0 and 1) (left) and on the front and back faces (2 and 3) (right), between the matching grid and the singular method.

A qualitative comparison of the displacement fields obtained in the two cases is shown in Fig. 2, right). For a more quantitative assessment, we monitor the average forces measured on the lateral faces. This choice is motivated by the fact that the differences between fully resolved (3D) and reduced (3D-1D) description shall be evaluated—from the practical point of view—at a *coarse* scale where the vessel cannot be fully resolved. A comparison of these average forces is plotted in Fig. 4, showing that the immersed method is able to reproduce the results of the fully resolved simulation very well, with a relative error of about 5% at the peak pressure instant.

### Vascular Tree

**Figure 5:**
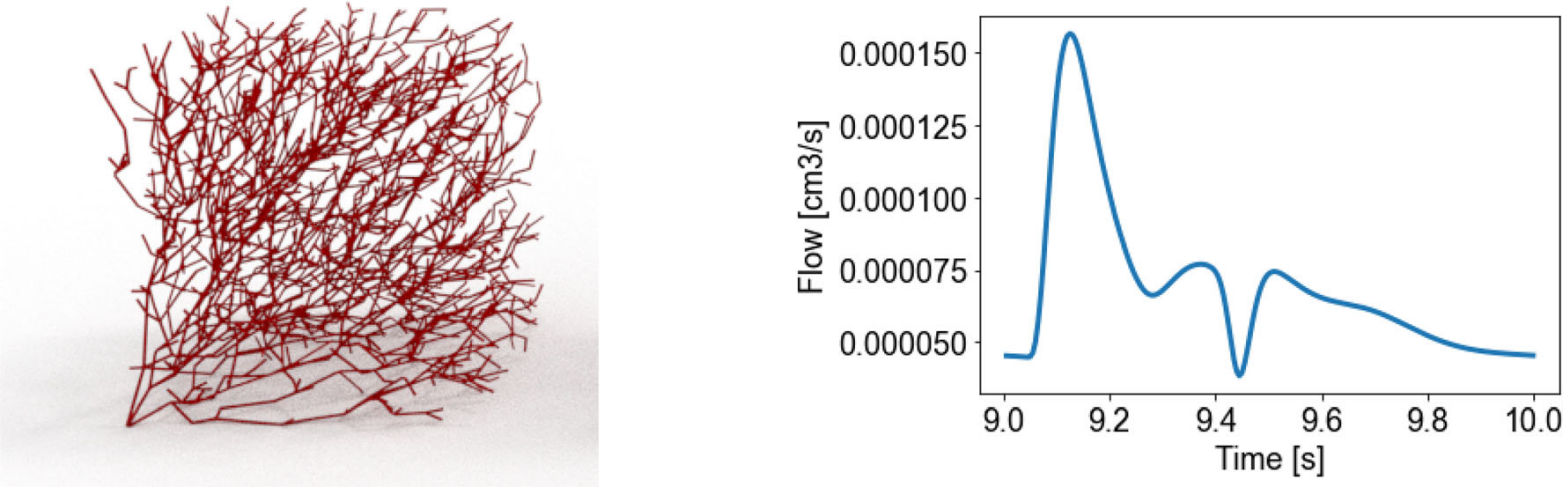
Left: Randomly distributed vasculature, irrorating two thousands randomly distributed points in the sample. The root point is situated in the lower left corner. The average radius of the vessels is about 0.012 mm. Right: Flow rate imposed at the bottom-left corner for simulating the one-dimensional hemodynamics.

Next, we consider a randomly generated vascular tree on a tissue sample mimicking a realistic setting used in elastography. The elastic tissue domain consists of a cubic tissue sample $3\ \text {mm}\times 3\ \text {mm}\times 3\ \text {mm}$ (of the order of voxel resolution of MRI scans), and elastic characteristics similar to those found in the human liver ($\mu =2kPa$, $\lambda =50$ kPa). The vessel distribution was constructed in silico, using the assumption that a vascular tree should fulfil the perfusion task with the minimum effort, while maintaining its anatomical structure. In general, this results in two or more competing mechanisms: on the one hand, one expects that the total length of the vasculature shall be minimized; on the other hand, other relevant physiological quantities shall be minimized as well, e.g., the time needed by oxygenated blood to reach perfusion points. In this work we generated the vascular tree using a publicly available code^1^ originally written to produce synthetic neuronal structures.^7^ The approach uses a simplified cost function, where the weight assigned to each edge of the tree is the weighted average of two factors: the *piping cost*, represented by the Euclidean distance between the irroration point and the connecting node in the tree, and a *total path length cost*, measuring the total path cost along the tree from the root to the irroration point. The resulting vasculature is shown in Fig. 5.

The one-dimensional hemodynamics was solved imposing a flow rate on the bottom-left corner of the tree (see Fig. 5, right) and using 3-elements Windkessel terminal models on the nodes situated on the lateral faces. Total inflow was computed based on domain volume and normal cerebral perfusion values (50 mL/min/100 g^4^). The inlet vessel radius was defined by assuming a physiological wall shear stress.^15^ The radii of the remaining vessels were assigned using Murray’s law^23^ and assuming that for a given mother vessel all daughters had the same radius. This automatically determined an equal flow split among daughters. Peripheral resistances for terminal network points were computed from estimated pressure at the outlets for the flow distribution resulting from the previous calibration step. That resistance was distributed in a three-element Windkessel, with 15% of total resistance assigned to the proximal resistance and the remaining resistance assigned to the distal resistance. The compliance of the three-element Windkessel was computed using the characteristic RC constant used in.^6^ At the outlet of the RCR Windkessel terminal elements, zero terminal pressure was assumed. Further details on the parametrization of the 1D model are provided as supplementary material. The simulation has been run for 10 seconds in order to reach a periodic state. For the coupling with the three-dimensional matrix, only the last period (time between 9 and 10 s) has been considered.

The elasticity problem for the tissue has been discretized using a uniform hexahedral mesh with element size of about 0.1 mm (32,800 elements in total). The hypersingular forces have been introduced discretizing the one-dimensional vascular network with a spacing between singular points of 0.04 mm, resulting in about 8600 singular points. On these points, the cross-sectional areas and the pressures obtained with the one-dimensional simulation have been used to compute the corresponding singular forces. In order to compute the forces, the vessel pressure has been rescaled taking into account an external pressure of $10^4$ Pa. For the regularization of the Dirac delta function (14), we used $\epsilon =0.1mm$, coinciding with the grid size used for the finite element approximation of the elasticity problem.

**Figure 6:**
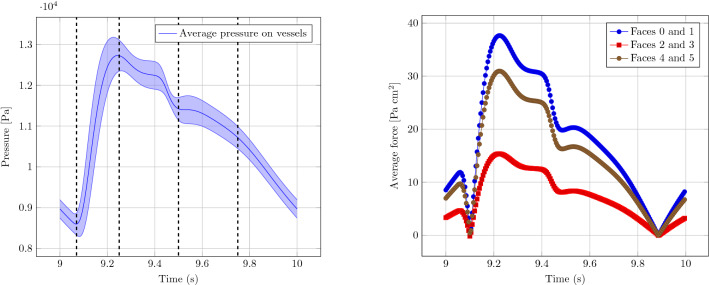
Left. Average pressure and standard deviation within the fluid vasculature over time in the considered period. The dashed lines indicated the time steps corresponding to the plots in Fig. 7. Right. Resulting absolute value of total forces on opposite faces, using the same numbering as in Fig. 2.

Figure 6 shows the results for the absolute value of the average forces on opposite lateral faces (right), together with the statistical information on the average and standard deviation of the pressure over the whole vasculature (left). In particular (see the notation introduced in Fig. 2), the sum of the contributions on faces 0 and 1 corresponds to the net force in the *x*-direction, while the sums on faces 2 and 3 (resp. on faces 4 and 5), correspond to the force along *y* (resp. along *z*). We remark here that average values on the vasculature network (left in Fig. 6) in general do not provide enough information to infer properties on the effective tissue scale. Even if Fig. 6 (right) shows a similar behaviour in terms of general values of the forces for the coupled multi-scale model, the relationship between the total forces in each direction are clearly different, and depend non-linearly and non-trivially from the topology of the vessel network, as well as from the solution of the one-dimensional network model. Even if one is only interested on the effect of the vasculature at a much larger scale w.r.t. to the scale of individual vessels, the coupling between the vasculature and the tissue cannot be neglected, as shown in Fig. 6 (right).

**Figure 7:**
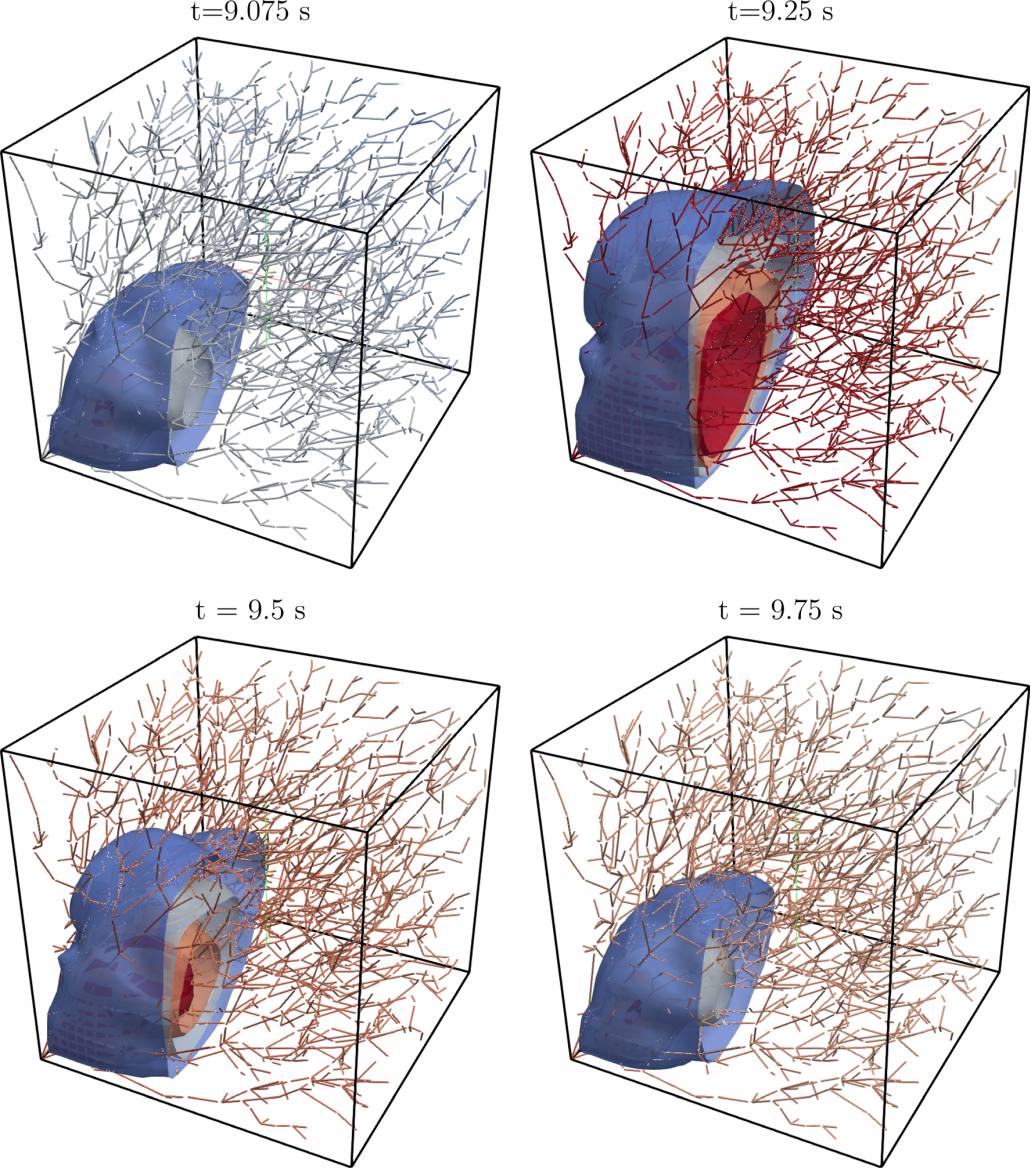
Contour plots of the 3D displacement field and plot of the 1D pressure on the vessels at different times. The considered time steps are also indicated with dashed lines in Fig. 6.

For a qualitative visualization, Fig. 7 shows the results for the internal displacement field at selected time steps, together with the varying pressure within the one-dimensional network.

One of the main practical advantages of the immersed method is that it allows to decouple the discretization of the three-dimensional tissue from the vasculature. In the case of a complex network of thin vessels, as the one considered in this section, a full three-dimensional discretization in which each vessel surface is discretized with, e.g., a triangular mesh, shall be considered practically unfeasible, due to the extremely high number of degrees of freedom resulting from the mesh. Nevertheless, in order to roughly quantify the gain in computational cost introduced by the immersed method, we sampled the one-dimensional network with a spacing $h_{1D}^{3D}$ comparable to the radius of the vessels and generated an unstrucutred tetrahedral mesh contraining these discrete hypersingular points to be vertices of the mesh tetrahedra. The mesh has been generated using TetGen.^28^ In particular, we considered $h_{1D}^{3D}$ equal to 0.01 and 0.005 mm, yielding about half million vertices and 2.8 millions vertices, respectively.

**Figure 8:**
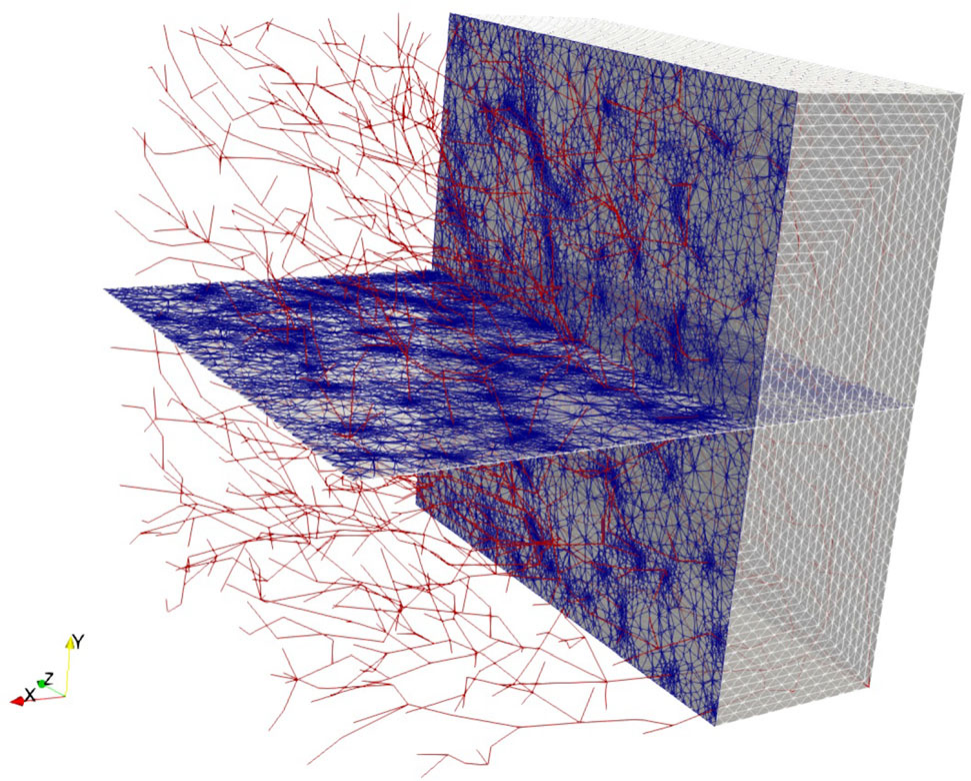
Visualization of the unstructured tetrahedral mesh obtained choosing $h_{1D}^{3D}=0.01\ \text {mm}$ and constraining the discrete hypersingular points to be vertices of tetrahedra. The red segments show the one-dimensional vasculature, while the blue lines depicts the intersection of the volume elements with the cutting planes.

Hence, the immersed method, used with the structured mesh on piecewise linear elements (less than 33,000 vertices), allows therefore a cost reduction of at least 90%. See also Fig. 8 and Table 1 for a more detailed comparison. We observe once more that the tetrahedral computational grids used for this comparison should still be considered insufficient for a simulation of a high fidelity full-scale model, since they neglect the discretization of the surfaces of the actual vessel network. Such computational grid would result in a number of elements which is at the very least five or six times larger w.r.t. the unstructured grid numbers reported in Table 1.

**Table 1:** Comparison of some characteristics of a structured mesh (used with the immersed method) and unstructured meshes obtained constraining the discrete hypersingular points to be vertices of tetrahedra.

Mesh	# nodes	# elements	Shortest edge (mm)
Structured (hexa)	34,800	32,800	0.1
Unstructured (tetra, $h_{1D}^{3D}=0.01\ \text {mm}$)	488,000	2,860,000	$1.1\cdot 10^{-3}$
Unstructured (tetra, $h_{1D}^{3D}=0.005\ \text {mm}$)	1,120,000	6,594,800	$1.7\cdot 10^{-4}$

The parameter $h_{1D}^{3D}$. refers to the sampling of the one-dimensional vessel when defining the location of the hypersingular sources (see Section 2.4)

## Discussion

We have proposed and validated *in silico* an efficient multiscale model for the simulation of an elastic tissue containing a thin vasculature. The fluid part is modeled using an *immersed* method, i.e., it is taken into account in the three-dimensional problem only through singular forces applied on a one-dimensional manifold. We considered a one-way coupling, from the blood flow on the tissue, neglecting the effect of the tissue response on hemodynamics. This back-coupling, as well as the extension of the model to the case of viscous and poroelastic tissues, taking into account vessel permeability and fluid exchange between tissue and vasculature, are currently objects of ongoing research.

The first advantage of the proposed approach is that it only requires one-dimensional information (centerlines, cross-sectional areas, mean pressures along the vessels) in order to define the coupling terms. This allows to solve the one-dimensional model problem independently, mapping then the necessary quantities onto the three-dimensional tissue domain. A further advantage of the immersed method is that it does not require the discretization of the vasculature within the three-dimensional mesh. In particular, it is sufficient to refine locally the mesh close to the vessel centerline.

To assess the performance of the proposed framework we discussed two different benchmarks. In the case of a simple bifurcation (Section 3.1) we compared the multiscale description with the results of a fully resolved three-dimensional problem. In particular, the results showed that the immersed method is able to reproduce the mechanical behavior of the effective tissue (i.e., the average forces on the boundary of the tissue sample) with an error below 5%. The goal of the the second test (a vascular tree containing more than 1800 vessel segments, Section 3.2) is to show the potential of the immersed method in the case of a complex scenario, where the full three-dimensional discretization of the problem might be practically unfeasible. The numerical results demonstrate that the method is able to consistently characterize the effective behavior of the tissue sample, following the internal fluid pressure, by up-scaling the effect of the vascular network, and providing non-trivial force information at the scale of the tissue sample. This information is not available through the one-dimensional solution of the vasculature network alone, and the coupling between the vasculature and the tissue should not be neglected.

It is worth to observe that the coupled formulation introduces an additional numerical parameter, $h_{1D}^{3D}$, related to the definition of the hypersingular points discretizing the one-dimensional manifold which is, a priori, decoupled from the three-dimensional finite element mesh used for the tissue. The optimal choice of the discretization characteristic size depends on the model and on the physical radius of the vessels. However, as observed in,^9^ the discretization sizes can be chosen in such a way that the error introduced by the multiscale modeling is comparable with the error of the numerical discretizations used in the three dimensional model. Moreover, since the mesh size is decoupled from the fluid vasculature size, different refinement strategies can be used to solve the elasticity problem (including local refinement strategies), that allow a further reduction of the computational and/or an increase in accuracy. A detailed assessment of the numerical parameters and of the influence of the spatial discretization on the effective tissue description is currently subject of ongoing research.

## Supplementary Information

Below is the link to the electronic supplementary material.

Electronic supplementary material 1 (PDF 477 kb)

## Footnotes

1 See https://github.com/pherbers/MST-Dendrites (setting the balancing factor to 0.5).

## References

[1] Alzetta G, Heltai L (2020). Multiscale modeling of fiber reinforced materials via non-matching immersed methods. Comput. Struct..

[2] Arndt D, Bangerth W, Blais B, Clevenger TC, Fehling M, Grayver AV, Heister T, Heltai L, Kronbichler M, Maier M, Munch P, Pelteret J-P, Rastak R, Tomas I, Turcksin B, Wang Z, Wells D (2020). The deal.II library version 9.2.. J. Num. Math..

[3] Arndt D, Bangerth W, Davydov D, Heister T, Heltai L, Kronbichler M, Maier M, Pelteret J-P, Turcksin B, Wells D (2021). The deal.II finite element library: Design, features, and insights. Comput. Math. Appl..

[4] Baron J-C (2001). Perfusion thresholds in human cerebral ischemia: Historical perspective and therapeutic implications. Cerebrovasc. Dis..

[5] Blanco PJ, Watanabe SM, Dari EA, Passos MARF, Feijóo RA (2014). Blood flow distribution in an anatomically detailed arterial network model: criteria and algorithms. Biomech. Model. Mechanobiol..

[6] Blanco PJ, Watanabe SM, Passos MRF, Lemos PA, Feijóo RA (2015). An anatomically detailed arterial network model for one-dimensional computational hemodynamics. IEEE Trans. Biomed. Eng..

[7] Cuntz H, Forstner F, Borst A, Häusser M (2010). One rule to grow them all: A general theory of neuronal branching and its practical application. PLoS Comput. Biol..

[8] Formaggia L, Quarteroni A, Veneziani A (2009). Cardiovascular Mathematics: Modeling and Simulation of the Circulatory System.

[9] Heltai L, Caiazzo A (2019). Multiscale modeling of vascularized tissues via nonmatching immersed methods. Int. J. Numer. Methods Biomed. Eng..

[10] Heltai L, Wenyu L (2020). A priori error estimates of regularized elliptic problems. Numer. Math..

[11] Hirsch S, Braun J, Sack I (2017). Magnetic Resonance Elastography: Physical Background And Medical Applications.

[12] Huberts W, Bode AS, Kroon W, Planken RN, Tordoir JHM, van de Vosse FN, Bosboom EMH (2012). A pulse wave propagation model to support decision-making in vascular access planning in the clinic. Med. Engnr. & Phys..

[13] Liang FY, Fukasaku K, Liu H, Takagi S (2011). A computational model study of the influence of the anatomy of the circle of willis on cerebral hyperperfusion following carotid artery surgery. Biomed. Eng..

[14] Lilaj L, Fischer T, Guo J, Braun J, Sack I, Hirsch S (2021). Separation of fluid and solid shear wave fields and quantification of coupling density by magnetic resonance poroelastography. Magn. Reson. Med..

[15] Lipowsky HH, Kovalcheck S, Zweifach BW (1978). The distribution of blood rheological parameters in the microvasculature of cat mesentery. Circ. Res..

[16] Matthys KS, Alastruey J, Peiró J, Khir AW, Segers P, Verdonck PR, Parker KH, Sherwin SJ (2007). Pulse wave propagation in a model human arterial network: Assessment of 1-D numerical simulations against in vitro measurements. J. Biomech..

[17] Müller LO, Blanco PJ, Watanabe SM, Feijóo RA (2016). A high-order local time stepping finite volume solver for one-dimensional blood flow simulations: Application to the ADAN model. Int. J. Num. Meth. Biomed. Eng..

[18] Müller LO, Caiazzo A, Blanco PJ (2019). Reduced-order unscented Kalman filter with observations in the frequency domain: Application to computational hemodynamics. IEEE Trans. Biomed. Eng..

[19] Müller LO, Toro EF (2014). A global multiscale model for the human circulation with emphasis on the venous system. Int. J. Numer. Meth. Biomed. Eng..

[20] Müller LO, Toro EF, Haacke EM, Utriainen D (2015). Impact of CCSVI on cerebral haemodynamics: A mathematical study using MRI angiographic and flow data. Phlebology.

[21] Müller LO, Leugering G, Blanco PJ (2016). Consistent treatment of viscoelastic effects at junctions in one-dimensional blood flow models. J. Comput. Phys..

[22] Müller LO, Blanco PJ, Watanabe SM, Feijóo RA (2016). A high-order local time stepping finite volume solver for one-dimensional blood flow simulations: Application to the ADAN model. Int. J. Numer. Meth. Biomed. Eng..

[23] Murray CD (1926). The physiological principle of minimum work. Proc. Natl. Acad. Sci. USA.

[24] Muthupillai R, Ehman RL (1996). Magnetic resonance elastography. Nat. Med..

[25] Mynard JP, Smolich JJ (2015). One-dimensional haemodynamic modeling and wave dynamics in the entire adult circulation. Ann. Biomed. Eng..

[26] Reymond P, Bohraus Y, Perren F, Lazeyras F, Stergiopulos N (2011). Validation of a patient-specific one-dimensional model of the systemic arterial tree. Am. J. Physiol..

[27] Sack I, Beierbach B, Hamhaber U, Klatt D, Braun J (2008). Non-invasive measurement of brain viscoelasticity using magnetic resonance elastography. NMR Biomed..

[28] Si H (2015). Tetgen, a delaunay-based quality tetrahedral mesh generator. ACM Trans. Math. Softw..

[29] Toro EF (2009). Riemann Solvers and Numerical Methods for Fluid Dynamics: A Practical Introduction.

[30] Toro EF, Millington R, Nejad LAM, Toro EF (2001). Towards very high order Godunov schemes. Godunov Methods: Theory and Applications.

[31] Wuerfel J, Paul F, Beierbach B, Hamhaber U, Klatt D, Papazoglou S, Zipp F, Martus P, Braun J, Sack I (2010). MR-elastography reveals degradation of tissue integrity in multiple sclerosis. Neuroimage.

