# Supplementary material for: Multiscale Coupling of One-dimensional Vascular Models and Elastic Tissues
Source: Ann Biomed Eng. 2021 Jul 19;49(12):3243–54. doi: 10.1007/s10439-021-02804-0 (PMC8671283; doi:10.1007/s10439-021-02804-0)
Supplement: Supplementary file 1 — Electronic supplementary material 1 (PDF 477 kb) [file 10439_2021_2804_MOESM1_ESM.pdf]

Inlet #	Outlet #	L [cm]	Inlet r [cm]	Outlet r [cm]	R prox [dyn s/cm2 /ml]	R dist	C [ml cm2 /dyn]
1	0	0,0395280	0,0071091	0,0071091	0	-1	-1
0	2	0,0209095	0,0049292	0,0049292	0	-1	-1
2	3	0,0143388	0,0039123	0,0039123	1520601540	8616742060	7,91796E-12
2	4	0,0330588	0,0039123	0,0039123	0	-1	-1
4	5	0,0163647	0,0027126	0,0027126	4554386225	25808188611	2,64362E-12
4	6	0,0312361	0,0027126	0,0027126	0	-1	-1
6	7	0,0118878	0,0021530	0,0021530	0	-1	-1
7	8	0,0106610	0,0017088	0,0017088	18170247637	1,02965E+11	6,62625E-13
7	9	0,0211775	0,0017088	0,0017088	0	-1	-1
9	10	0,0190271	0,0010765	0,0010765	72419533301	4,10377E+11	1,66254E-13
9	11	0,0205151	0,0010765	0,0010765	72404366593	4,10291E+11	1,66289E-13
9	12	0,0255307	0,0010765	0,0010765	0	-1	-1
12	13	0,0289655	0,0010765	0,0010765	0	-1	-1
13	14	0,0148353	0,0008544	0,0008544	0	-1	-1
14	15	0,0127313	0,0008544	0,0008544	0	-1	-1
15	16	0,0169725	0,0008544	0,0008544	0	-1	-1
16	17	0,0102819	0,0006782	0,0006782	2,85279E+11	1,61658E+12	4,22046E-14
16	18	0,0120622	0,0006782	0,0006782	2,85164E+11	1,61593E+12	4,22216E-14
13	19	0,0309696	0,0008544	0,0008544	0	-1	-1
19	20	0,0115494	0,0008544	0,0008544	1,43024E+11	8,10469E+11	8,41822E-14
9	21	0,0269581	0,0010765	0,0010765	0	-1	-1
21	22	0,0137446	0,0007464	0,0007464	2,1641E+11	1,22632E+12	5,56355E-14
21	23	0,0198054	0,0007464	0,0007464	0	-1	-1
23	24	0,0165829	0,0007464	0,0007464	2,15411E+11	1,22066E+12	5,58934E-14
21	25	0,0311803	0,0007464	0,0007464	0	-1	-1
25	26	0,0210870	0,0004702	0,0004702	0	-1	-1
26	27	0,0210262	0,0004702	0,0004702	0	-1	-1
27	28	0,0249645	0,0004702	0,0004702	0	-1	-1
28	29	0,0282750	0,0003732	0,0003732	0	-1	-1
29	30	0,0143994	0,0002962	0,0002962	3,30962E+12	1,87545E+13	3,6379E-15
29	31	0,0178409	0,0002962	0,0002962	0	-1	-1
31	32	0,0164542	0,0002962	0,0002962	3,27425E+12	1,85541E+13	3,6772E-15
28	33	0,0303194	0,0003732	0,0003732	0	-1	-1
33	34	0,0378135	0,0003732	0,0003732	0	-1	-1
34	35	0,0171792	0,0002962	0,0002962	0	-1	-1
35	36	0,0275796	0,0002962	0,0002962	0	-1	-1
36	37	0,0155656	0,0002351	0,0002351	0	-1	-1
37	38	0,0212758	0,0002351	0,0002351	0	-1	-1
38	39	0,0171388	0,0002351	0,0002351	0	-1	-1
39	40	0,0095877	0,0002351	0,0002351	0	-1	-1
40	41	0,0187460	0,0002351	0,0002351	0	-1	-1
41	42	0,0063628	0,0002351	0,0002351	6,00149E+12	3,40084E+13	2,00618E-15
36	43	0,0271405	0,0002351	0,0002351	0	-1	-1
43	44	0,0285640	0,0002351	0,0002351	6,14921E+12	3,48455E+13	1,95798E-15
34	45	0,0203451	0,0002962	0,0002962	0	-1	-1
45	46	0,0296704	0,0002962	0,0002962	0	-1	-1
46	47	0,0209163	0,0002962	0,0002962	0	-1	-1
47	48	0,0108896	0,0002962	0,0002962	3,1335E+12	1,77565E+13	3,84238E-15
25	49	0,0279924	0,0004702	0,0004702	0	-1	-1
49	50	0,0171052	0,0003260	0,0003260	0	-1	-1
50	51	0,0134739	0,0003260	0,0003260	2,52713E+12	1,43204E+13	4,76433E-15
49	52	0,0228524	0,0003260	0,0003260	0	-1	-1

52	53	0,0156213	0,0003260	0,0003260	2,51756E+12	1,42662E+13	4,78243E-15
49	54	0,0269705	0,0003260	0,0003260	0	-1	-1
54	55	0,0145388	0,0002588	0,0002588	5,01861E+12	2,84388E+13	2,39908E-15
54	56	0,0205611	0,0002588	0,0002588	0	-1	-1
56	57	0,0168366	0,0002588	0,0002588	4,94883E+12	2,80434E+13	2,43291E-15
25	58	0,0343928	0,0004702	0,0004702	0	-1	-1
58	59	0,0151067	0,0004702	0,0004702	8,48703E+11	4,80932E+12	1,41864E-14
25	60	0,0368301	0,0004702	0,0004702	0	-1	-1
60	61	0,0288363	0,0004702	0,0004702	0	-1	-1
61	62	0,0175996	0,0003260	0,0003260	2,51121E+12	1,42302E+13	4,79454E-15
61	63	0,0213647	0,0003260	0,0003260	0	-1	-1
63	64	0,0062535	0,0002588	0,0002588	4,9942E+12	2,83004E+13	2,41081E-15
63	65	0,0152374	0,0002588	0,0002588	0	-1	-1
65	66	0,0232756	0,0002588	0,0002588	0	-1	-1
66	67	0,0161420	0,0002588	0,0002588	0	-1	-1
67	68	0,0127426	0,0002054	0,0002054	9,59483E+12	5,43707E+13	1,25485E-15
67	69	0,0253715	0,0002054	0,0002054	9,49767E+12	5,38202E+13	1,26769E-15
61	70	0,0322225	0,0003260	0,0003260	0	-1	-1
70	71	0,0287054	0,0002588	0,0002588	0	-1	-1
71	72	0,0082931	0,0002588	0,0002588	0	-1	-1
72	73	0,0193968	0,0002588	0,0002588	0	-1	-1
73	74	0,0277702	0,0002588	0,0002588	0	-1	-1
74	75	0,0102385	0,0001630	0,0001630	1,87216E+13	1,06089E+14	6,4311E-16
74	76	0,0183172	0,0001630	0,0001630	1,8565E+13	1,05202E+14	6,48535E-16
74	77	0,0185820	0,0001630	0,0001630	1,85599E+13	1,05173E+14	6,48715E-16
74	78	0,0195286	0,0001630	0,0001630	1,85415E+13	1,05069E+14	6,49357E-16
70	79	0,0323452	0,0002588	0,0002588	0	-1	-1
79	80	0,0029257	0,0001794	0,0001794	1,4626E+13	8,28809E+13	8,23194E-16
79	81	0,0098352	0,0001794	0,0001794	0	-1	-1
81	82	0,0077534	0,0001794	0,0001794	1,44324E+13	8,17833E+13	8,34242E-16
79	83	0,0100624	0,0001794	0,0001794	0	-1	-1
83	84	0,0091856	0,0001794	0,0001794	1,44104E+13	8,16591E+13	8,35511E-16
6	85	0,0337745	0,0021530	0,0021530	0	-1	-1
85	86	0,0184643	0,0017088	0,0017088	18129836947	1,02736E+11	6,64102E-13
85	87	0,0193810	0,0017088	0,0017088	0	-1	-1
87	88	0,0273692	0,0017088	0,0017088	0	-1	-1
88	89	0,0173155	0,0013563	0,0013563	0	-1	-1
89	90	0,0180883	0,0010765	0,0010765	0	-1	-1
90	91	0,0155439	0,0007464	0,0007464	0	-1	-1
91	92	0,0073531	0,0007464	0,0007464	2,1503E+11	1,2185E+12	5,59925E-14
90	93	0,0192558	0,0007464	0,0007464	2,15191E+11	1,21941E+12	5,59507E-14
90	94	0,0205495	0,0007464	0,0007464	2,15134E+11	1,21909E+12	5,59655E-14
89	95	0,0253735	0,0010765	0,0010765	0	-1	-1
95	96	0,0223158	0,0008544	0,0008544	0	-1	-1
96	97	0,0200454	0,0006782	0,0006782	0	-1	-1
97	98	0,0125193	0,0004702	0,0004702	0	-1	-1
98	99	0,0175987	0,0003732	0,0003732	0	-1	-1
99	100	0,0080588	0,0003732	0,0003732	1,68676E+12	9,55831E+12	7,13799E-15
98	101	0,0187265	0,0003732	0,0003732	1,69165E+12	9,58602E+12	7,11735E-15
97	102	0,0146808	0,0004702	0,0004702	8,51827E+11	4,82702E+12	1,41344E-14
97	103	0,0150753	0,0004702	0,0004702	0	-1	-1
103	104	0,0179029	0,0004702	0,0004702	0	-1	-1
104	105	0,0132895	0,0004702	0,0004702	0	-1	-1

105	106	0,0310296	0,0004702	0,0004702	0	-1	-1
106	107	0,0072445	0,0003732	0,0003732	1,66347E+12	9,42635E+12	7,23791E-15
106	108	0,0173014	0,0003732	0,0003732	0	-1	-1
108	109	0,0141151	0,0003732	0,0003732	0	-1	-1
109	110	0,0217124	0,0003732	0,0003732	0	-1	-1
110	111	0,0370637	0,0003732	0,0003732	1,60495E+12	9,09469E+12	7,50185E-15
96	112	0,0232143	0,0006782	0,0006782	0	-1	-1
112	113	0,0153145	0,0006782	0,0006782	2,84116E+11	1,60999E+12	4,23772E-14
95	114	0,0413811	0,0008544	0,0008544	0	-1	-1
114	115	0,0211749	0,0005924	0,0005924	0	-1	-1
115	116	0,0098725	0,0004108	0,0004108	0	-1	-1
116	117	0,0378623	0,0004108	0,0004108	0	-1	-1
117	118	0,0194873	0,0003260	0,0003260	0	-1	-1
118	119	0,0132709	0,0002588	0,0002588	0	-1	-1
119	120	0,0158078	0,0002588	0,0002588	4,88531E+12	2,76834E+13	2,46455E-15
118	121	0,0148245	0,0002588	0,0002588	4,92883E+12	2,793E+13	2,44279E-15
117	122	0,0255341	0,0003260	0,0003260	0	-1	-1
122	123	0,0157295	0,0002588	0,0002588	4,91141E+12	2,78313E+13	2,45145E-15
122	124	0,0168630	0,0002588	0,0002588	0	-1	-1
124	125	0,0204991	0,0002054	0,0002054	9,6582E+12	5,47298E+13	1,24662E-15
124	126	0,0265079	0,0002054	0,0002054	0	-1	-1
126	127	0,0156878	0,0002054	0,0002054	9,49129E+12	5,3784E+13	1,26854E-15
115	128	0,0141776	0,0004108	0,0004108	0	-1	-1
128	129	0,0105151	0,0004108	0,0004108	0	-1	-1
129	130	0,0328554	0,0004108	0,0004108	0	-1	-1
130	131	0,0011260	0,0004108	0,0004108	0	-1	-1
131	132	0,0239776	0,0003260	0,0003260	2,47108E+12	1,40028E+13	4,87238E-15
131	133	0,0301970	0,0003260	0,0003260	0	-1	-1
133	134	0,0215015	0,0003260	0,0003260	2,4375E+12	1,38125E+13	4,93952E-15
115	135	0,0179784	0,0004108	0,0004108	1,26963E+12	7,19459E+12	9,4831E-15
114	136	0,0246946	0,0005924	0,0005924	4,25702E+11	2,41231E+12	2,82829E-14
114	137	0,0275756	0,0005924	0,0005924	0	-1	-1
137	138	0,0278471	0,0005924	0,0005924	0	-1	-1
138	139	0,0043460	0,0005924	0,0005924	4,21804E+11	2,39022E+12	2,85442E-14
88	140	0,0180564	0,0013563	0,0013563	36095827878	2,04543E+11	3,33558E-13
4	141	0,0513200	0,0027126	0,0027126	0	-1	-1
141	142	0,0112600	0,0017088	0,0017088	0	-1	-1
142	143	0,0100457	0,0017088	0,0017088	18147997257	1,02839E+11	6,63438E-13
141	144	0,0279182	0,0017088	0,0017088	0	-1	-1
144	145	0,0144345	0,0011848	0,0011848	0	-1	-1
145	146	0,0058612	0,0009404	0,0009404	1,08521E+11	6,14954E+11	1,10947E-13
145	147	0,0120157	0,0009404	0,0009404	1,08414E+11	6,14343E+11	1,11057E-13
144	148	0,0165374	0,0011848	0,0011848	54297290795	3,07685E+11	2,21743E-13
144	149	0,0167950	0,0011848	0,0011848	0	-1	-1
149	150	0,0105865	0,0011848	0,0011848	0	-1	-1
150	151	0,0121320	0,0011848	0,0011848	0	-1	-1
151	152	0,0072066	0,0011848	0,0011848	54087661122	3,06497E+11	2,22603E-13
141	153	0,0437737	0,0017088	0,0017088	0	-1	-1
153	154	0,0088212	0,0011848	0,0011848	0	-1	-1
154	155	0,0023451	0,0006929	0,0006929	2,71233E+11	1,53699E+12	4,43901E-14
154	156	0,0155075	0,0006929	0,0006929	0	-1	-1
156	157	0,0205063	0,0006929	0,0006929	0	-1	-1
157	158	0,0073519	0,0004804	0,0004804	8,05813E+11	4,56628E+12	1,49415E-14

157	159	0,0215754	0,0004804	0,0004804	0	-1	-1
159	160	0,0212381	0,0003813	0,0003813	0	-1	-1
160	161	0,0208847	0,0003027	0,0003027	0	-1	-1
161	162	0,0278486	0,0003027	0,0003027	0	-1	-1
162	163	0,0124354	0,0001907	0,0001907	1,22777E+13	6,95737E+13	9,80644E-16
162	164	0,0154147	0,0001907	0,0001907	1,22468E+13	6,93988E+13	9,83116E-16
162	165	0,0154283	0,0001907	0,0001907	0	-1	-1
165	166	0,0113495	0,0001907	0,0001907	1,21291E+13	6,87318E+13	9,92656E-16
162	167	0,0155468	0,0001907	0,0001907	1,22455E+13	6,9391E+13	9,83226E-16
160	168	0,0306396	0,0003027	0,0003027	3,13115E+12	1,77432E+13	3,84525E-15
159	169	0,0246922	0,0003813	0,0003813	0	-1	-1
169	170	0,0102616	0,0002644	0,0002644	4,73625E+12	2,68387E+13	2,54211E-15
169	171	0,0166966	0,0002644	0,0002644	0	-1	-1
171	172	0,0066123	0,0002098	0,0002098	9,38977E+12	5,32087E+13	1,28225E-15
171	173	0,0432161	0,0002098	0,0002098	0	-1	-1
173	174	0,0071104	0,0001455	0,0001455	2,7177E+13	1,54003E+14	4,43024E-16
173	175	0,0162756	0,0001455	0,0001455	0	-1	-1
175	176	0,0114485	0,0001455	0,0001455	2,65475E+13	1,50436E+14	4,5353E-16
173	177	0,0199430	0,0001455	0,0001455	2,67851E+13	1,51782E+14	4,49506E-16
169	178	0,0265284	0,0002644	0,0002644	0	-1	-1
178	179	0,0295578	0,0002644	0,0002644	0	-1	-1
179	180	0,0203220	0,0002098	0,0002098	9,07232E+12	5,14098E+13	1,32712E-15
179	181	0,0253444	0,0002098	0,0002098	9,03687E+12	5,12089E+13	1,33233E-15
157	182	0,0317650	0,0004804	0,0004804	0	-1	-1
182	183	0,0060805	0,0003813	0,0003813	0	-1	-1
183	184	0,0098765	0,0003813	0,0003813	1,58875E+12	9,00292E+12	7,57832E-15
182	185	0,0102102	0,0003813	0,0003813	0	-1	-1
185	186	0,0286517	0,0003813	0,0003813	1,57392E+12	8,91889E+12	7,64973E-15
154	187	0,0194871	0,0006929	0,0006929	0	-1	-1
187	188	0,0461390	0,0006929	0,0006929	0	-1	-1
188	189	0,0067352	0,0004052	0,0004052	0	-1	-1
189	190	0,0189517	0,0003216	0,0003216	2,64367E+12	1,49808E+13	4,5543E-15
189	191	0,0253362	0,0003216	0,0003216	0	-1	-1
191	192	0,0243051	0,0003216	0,0003216	2,60441E+12	1,47583E+13	4,62296E-15
188	193	0,0175102	0,0004052	0,0004052	1,32849E+12	7,5281E+12	9,06299E-15
188	194	0,0202669	0,0004052	0,0004052	0	-1	-1
194	195	0,0304383	0,0004052	0,0004052	0	-1	-1
195	196	0,0143421	0,0002810	0,0002810	0	-1	-1
196	197	0,0119157	0,0002810	0,0002810	3,87722E+12	2,19709E+13	3,10533E-15
195	198	0,0219137	0,0002810	0,0002810	0	-1	-1
198	199	0,0195000	0,0002810	0,0002810	0	-1	-1
199	200	0,0097303	0,0002810	0,0002810	3,82256E+12	2,16611E+13	3,14974E-15
195	201	0,0310043	0,0002810	0,0002810	0	-1	-1
201	202	0,0233027	0,0002810	0,0002810	3,81561E+12	2,16218E+13	3,15548E-15
188	203	0,0224780	0,0004052	0,0004052	0	-1	-1
203	204	0,0023596	0,0002553	0,0002553	5,29626E+12	3,00121E+13	2,27332E-15
203	205	0,0184666	0,0002553	0,0002553	5,24433E+12	2,97179E+13	2,29582E-15
203	206	0,0325709	0,0002553	0,0002553	0	-1	-1
206	207	0,0273189	0,0001493	0,0001493	0	-1	-1
207	208	0,0099869	0,0001185	0,0001185	4,9789E+13	2,82138E+14	2,41822E-16
207	209	0,0200786	0,0001185	0,0001185	0	-1	-1
209	210	0,0298377	0,0001185	0,0001185	4,70158E+13	2,66423E+14	2,56085E-16
206	211	0,0303784	0,0001493	0,0001493	2,5157E+13	1,42556E+14	4,78597E-16

206	212	0,0347504	0,0001493	0,0001493	0	-1	-1
212	213	0,0225613	0,0001185	0,0001185	4,8506E+13	2,74868E+14	2,48218E-16
212	214	0,0245830	0,0001185	0,0001185	4,83656E+13	2,74072E+14	2,48938E-16
206	215	0,0374724	0,0001493	0,0001493	0	-1	-1
215	216	0,0192179	0,0001035	0,0001035	7,25926E+13	4,11358E+14	1,65858E-16
215	217	0,0378301	0,0001035	0,0001035	0	-1	-1
217	218	0,0243954	0,0000822	0,0000822	0	-1	-1
218	219	0,0096111	0,0000822	0,0000822	0	-1	-1
219	220	0,0096954	0,0000822	0,0000822	1,27613E+14	7,23141E+14	9,43482E-17
217	221	0,0249201	0,0000822	0,0000822	0	-1	-1
221	222	0,0163405	0,0000822	0,0000822	1,28347E+14	7,27298E+14	9,38089E-17
215	223	0,0442093	0,0001035	0,0001035	0	-1	-1
223	224	0,0122925	0,0001035	0,0001035	0	-1	-1
224	225	0,0142628	0,0001035	0,0001035	6,64453E+13	3,76523E+14	1,81203E-16
206	226	0,0572453	0,0001493	0,0001493	0	-1	-1
226	227	0,0220806	0,0001185	0,0001185	0	-1	-1
227	228	0,0191245	0,0001185	0,0001185	0	-1	-1
228	229	0,0312533	0,0001185	0,0001185	4,38005E+13	2,48203E+14	2,74884E-16
226	230	0,0254664	0,0001185	0,0001185	0	-1	-1
230	231	0,0379195	0,0001185	0,0001185	0	-1	-1
231	232	0,0229723	0,0001185	0,0001185	0	-1	-1
232	233	0,0129356	0,0001185	0,0001185	4,19367E+13	2,37641E+14	2,87101E-16
203	234	0,0459267	0,0002553	0,0002553	0	-1	-1
234	235	0,0252055	0,0002553	0,0002553	5,07455E+12	2,87558E+13	2,37264E-15
188	236	0,0306272	0,0004052	0,0004052	1,32183E+12	7,49036E+12	9,10865E-15
154	237	0,0226939	0,0006929	0,0006929	0	-1	-1
237	238	0,0147046	0,0005500	0,0005500	0	-1	-1
238	239	0,0208700	0,0005500	0,0005500	5,34727E+11	3,03012E+12	2,25163E-14
237	240	0,0414283	0,0005500	0,0005500	0	-1	-1
240	241	0,0057172	0,0003813	0,0003813	0	-1	-1
241	242	0,0208846	0,0003813	0,0003813	1,58433E+12	8,97787E+12	7,59947E-15
240	243	0,0235019	0,0003813	0,0003813	1,58634E+12	8,98925E+12	7,58985E-15
240	244	0,0272470	0,0003813	0,0003813	1,58391E+12	8,97551E+12	7,60147E-15
154	245	0,0231552	0,0006929	0,0006929	0	-1	-1
245	246	0,0182049	0,0006929	0,0006929	0	-1	-1
246	247	0,0123680	0,0005500	0,0005500	5,35983E+11	3,03723E+12	2,24635E-14
246	248	0,0287877	0,0005500	0,0005500	5,33526E+11	3,02331E+12	2,2567E-14
153	249	0,0235488	0,0011848	0,0011848	54172239510	3,06976E+11	2,22255E-13
153	250	0,0275397	0,0011848	0,0011848	0	-1	-1
250	251	0,0119456	0,0011848	0,0011848	54061555211	3,06349E+11	2,2271E-13
141	252	0,0530001	0,0017088	0,0017088	18097121060	1,0255E+11	6,65303E-13
0	253	0,0253985	0,0049292	0,0049292	0	-1	-1
253	254	0,0183802	0,0049292	0,0049292	0	-1	-1
254	255	0,0056255	0,0039123	0,0039123	0	-1	-1
255	256	0,0069885	0,0039123	0,0039123	0	-1	-1
256	257	0,0446017	0,0039123	0,0039123	0	-1	-1
257	258	0,0253209	0,0039123	0,0039123	0	-1	-1
258	259	0,0150283	0,0027126	0,0027126	4542869672	25742928142	2,65032E-12
258	260	0,0226501	0,0027126	0,0027126	4540942838	25732009415	2,65145E-12
258	261	0,0397681	0,0027126	0,0027126	0	-1	-1
261	262	0,0282102	0,0021530	0,0021530	0	-1	-1
262	263	0,0065472	0,0021530	0,0021530	9051089178	51289505341	1,33023E-12
261	264	0,0341246	0,0021530	0,0021530	0	-1	-1

264	265	0,0137355	0,0014928	0,0014928	0	-1	-1
265	266	0,0108424	0,0014928	0,0014928	27086733676	1,53491E+11	4,44501E-13
264	267	0,0307406	0,0014928	0,0014928	0	-1	-1
267	268	0,0193476	0,0010351	0,0010351	80978510262	4,58878E+11	1,48682E-13
267	269	0,0197648	0,0010351	0,0010351	0	-1	-1
269	270	0,0171494	0,0010351	0,0010351	80769018439	4,57691E+11	1,49068E-13
267	271	0,0400957	0,0010351	0,0010351	0	-1	-1
271	272	0,0200884	0,0008215	0,0008215	1,60858E+11	9,11531E+11	7,48488E-14
271	273	0,0272542	0,0008215	0,0008215	0	-1	-1
273	274	0,0279165	0,0006520	0,0006520	3,19172E+11	1,80864E+12	3,77228E-14
273	275	0,0386069	0,0006520	0,0006520	0	-1	-1
275	276	0,0132336	0,0004521	0,0004521	0	-1	-1
276	277	0,0145308	0,0004521	0,0004521	9,45992E+11	5,36062E+12	1,27275E-14
275	278	0,0134326	0,0004521	0,0004521	0	-1	-1
278	279	0,0140659	0,0004521	0,0004521	9,46079E+11	5,36111E+12	1,27263E-14
275	280	0,0274739	0,0004521	0,0004521	0	-1	-1
280	281	0,0113430	0,0004521	0,0004521	9,42371E+11	5,3401E+12	1,27764E-14
264	282	0,0397130	0,0014928	0,0014928	0	-1	-1
282	283	0,0097290	0,0011848	0,0011848	0	-1	-1
283	284	0,0143388	0,0008215	0,0008215	0	-1	-1
284	285	0,0297884	0,0006520	0,0006520	0	-1	-1
285	286	0,0180616	0,0006520	0,0006520	3,1965E+11	1,81135E+12	3,76665E-14
284	287	0,0483062	0,0006520	0,0006520	0	-1	-1
287	288	0,0192637	0,0006520	0,0006520	0	-1	-1
288	289	0,0298590	0,0006520	0,0006520	3,15895E+11	1,79007E+12	3,81141E-14
283	290	0,0237164	0,0008215	0,0008215	0	-1	-1
290	291	0,0258964	0,0008215	0,0008215	1,60576E+11	9,09933E+11	7,49803E-14
283	292	0,0297288	0,0008215	0,0008215	0	-1	-1
292	293	0,0263028	0,0008215	0,0008215	0	-1	-1
293	294	0,0168948	0,0008215	0,0008215	1,59876E+11	9,05963E+11	7,53088E-14
282	295	0,0133732	0,0011848	0,0011848	53997153637	3,05984E+11	2,22976E-13
254	296	0,0566659	0,0039123	0,0039123	0	-1	-1
296	297	0,0148205	0,0031052	0,0031052	3031953739	17181071189	3,97106E-12
296	298	0,0199346	0,0031052	0,0031052	0	-1	-1
298	299	0,0313788	0,0031052	0,0031052	0	-1	-1
299	300	0,0193588	0,0021530	0,0021530	9067410553	51381993135	1,32784E-12
299	301	0,0267661	0,0021530	0,0021530	0	-1	-1
301	302	0,0212928	0,0013563	0,0013563	36164640889	2,04933E+11	3,32924E-13
301	303	0,0281774	0,0013563	0,0013563	36136793322	2,04775E+11	3,3318E-13
301	304	0,0302664	0,0013563	0,0013563	0	-1	-1
304	305	0,0184039	0,0013563	0,0013563	36053902133	2,04305E+11	3,33946E-13
301	306	0,0350743	0,0013563	0,0013563	0	-1	-1
306	307	0,0179172	0,0013563	0,0013563	0	-1	-1
307	308	0,0079501	0,0009404	0,0009404	0	-1	-1
308	309	0,0157299	0,0006520	0,0006520	0	-1	-1
309	310	0,0177781	0,0006520	0,0006520	3,21373E+11	1,82111E+12	3,74645E-14
308	311	0,0213238	0,0006520	0,0006520	0	-1	-1
311	312	0,0154280	0,0004521	0,0004521	9,61832E+11	5,45038E+12	1,25178E-14
311	313	0,0162588	0,0004521	0,0004521	0	-1	-1
313	314	0,0124993	0,0004521	0,0004521	9,57465E+11	5,42563E+12	1,25749E-14
311	315	0,0270111	0,0004521	0,0004521	0	-1	-1
315	316	0,0110384	0,0004521	0,0004521	9,54421E+11	5,40838E+12	1,26151E-14
308	317	0,0215806	0,0006520	0,0006520	0	-1	-1

317	318	0,0141580	0,0005175	0,0005175	0	-1	-1
318	319	0,0254992	0,0004108	0,0004108	1,27144E+12	7,20484E+12	9,46962E-15
318	320	0,0291945	0,0004108	0,0004108	1,26966E+12	7,19477E+12	9,48287E-15
317	321	0,0203971	0,0005175	0,0005175	6,40661E+11	3,63041E+12	1,87932E-14
307	322	0,0084456	0,0009404	0,0009404	1,07961E+11	6,11782E+11	1,11522E-13
307	323	0,0238152	0,0009404	0,0009404	0	-1	-1
323	324	0,0473839	0,0009404	0,0009404	0	-1	-1
324	325	0,0108477	0,0007464	0,0007464	0	-1	-1
325	326	0,0118857	0,0007464	0,0007464	0	-1	-1
326	327	0,0084210	0,0007464	0,0007464	2,12352E+11	1,20333E+12	5,66985E-14
324	328	0,0130430	0,0007464	0,0007464	0	-1	-1
328	329	0,0141655	0,0005924	0,0005924	0	-1	-1
329	330	0,0152421	0,0005924	0,0005924	0	-1	-1
330	331	0,0112576	0,0005924	0,0005924	4,21783E+11	2,39011E+12	2,85456E-14
328	332	0,0188502	0,0005924	0,0005924	0	-1	-1
332	333	0,0195571	0,0004702	0,0004702	8,42939E+11	4,77665E+12	1,42834E-14
332	334	0,0320097	0,0004702	0,0004702	0	-1	-1
334	335	0,0087742	0,0003732	0,0003732	1,67271E+12	9,47871E+12	7,19793E-15
334	336	0,0113233	0,0003732	0,0003732	1,67091E+12	9,46851E+12	7,20568E-15
299	337	0,0273999	0,0021530	0,0021530	0	-1	-1
337	338	0,0155482	0,0017088	0,0017088	0	-1	-1
338	339	0,0161450	0,0017088	0,0017088	0	-1	-1
339	340	0,0094414	0,0013563	0,0013563	36109215001	2,04619E+11	3,33435E-13
339	341	0,0126771	0,0013563	0,0013563	0	-1	-1
341	342	0,0141802	0,0013563	0,0013563	0	-1	-1
342	343	0,0147603	0,0010765	0,0010765	71927095330	4,07587E+11	1,67393E-13
342	344	0,0162352	0,0010765	0,0010765	71912062936	4,07502E+11	1,67428E-13
337	345	0,0321157	0,0017088	0,0017088	0	-1	-1
345	346	0,0205458	0,0017088	0,0017088	0	-1	-1
346	347	0,0119424	0,0011848	0,0011848	0	-1	-1
347	348	0,0219246	0,0009404	0,0009404	1,07691E+11	6,10247E+11	1,11802E-13
347	349	0,0320168	0,0009404	0,0009404	0	-1	-1
349	350	0,0250906	0,0009404	0,0009404	1,07075E+11	6,06758E+11	1,12445E-13
346	351	0,0290321	0,0011848	0,0011848	53918492330	3,05538E+11	2,23301E-13
346	352	0,0368101	0,0011848	0,0011848	0	-1	-1
352	353	0,0219446	0,0011848	0,0011848	53712058495	3,04368E+11	2,24159E-13
0	354	0,0264926	0,0049292	0,0049292	0	-1	-1
354	355	0,0203520	0,0034177	0,0034177	2279728795	12918463173	5,28136E-12
354	356	0,0249273	0,0034177	0,0034177	0	-1	-1
356	357	0,0127220	0,0023697	0,0023697	6832286922	38716292559	1,76223E-12
356	358	0,0232576	0,0023697	0,0023697	6827713575	38690376925	1,76341E-12
356	359	0,0235253	0,0023697	0,0023697	0	-1	-1
359	360	0,0195406	0,0023697	0,0023697	0	-1	-1
360	361	0,0051256	0,0023697	0,0023697	0	-1	-1
361	362	0,0385723	0,0023697	0,0023697	0	-1	-1
362	363	0,0176914	0,0018808	0,0018808	0	-1	-1
363	364	0,0155712	0,0013041	0,0013041	0	-1	-1
364	365	0,0079010	0,0013041	0,0013041	40631738730	2,30247E+11	2,96322E-13
363	366	0,0168895	0,0013041	0,0013041	0	-1	-1
366	367	0,0046903	0,0013041	0,0013041	40640694878	2,30297E+11	2,96256E-13
363	368	0,0421951	0,0013041	0,0013041	0	-1	-1
368	369	0,0117391	0,0013041	0,0013041	0	-1	-1
369	370	0,0196248	0,0010351	0,0010351	80741102848	4,57533E+11	1,49119E-13

369	371	0,0273706	0,0010351	0,0010351	0	-1	-1
371	372	0,0157650	0,0010351	0,0010351	80460721069	4,55944E+11	1,49639E-13
362	373	0,0189513	0,0018808	0,0018808	0	-1	-1
373	374	0,0283424	0,0018808	0,0018808	0	-1	-1
374	375	0,0119453	0,0014928	0,0014928	27064200351	1,53364E+11	4,44871E-13
374	376	0,0160569	0,0014928	0,0014928	0	-1	-1
376	377	0,0239856	0,0014928	0,0014928	26986757026	1,52925E+11	4,46147E-13
354	378	0,0290163	0,0034177	0,0034177	0	-1	-1
378	379	0,0216592	0,0034177	0,0034177	0	-1	-1
379	380	0,0155493	0,0034177	0,0034177	0	-1	-1
380	381	0,0170870	0,0027126	0,0027126	0	-1	-1
381	382	0,0213816	0,0027126	0,0027126	4540528096	25729659211	2,65169E-12
380	383	0,0218772	0,0027126	0,0027126	0	-1	-1
383	384	0,0208568	0,0027126	0,0027126	0	-1	-1
384	385	0,0170702	0,0021530	0,0021530	9068025423	51385477398	1,32775E-12
384	386	0,0316781	0,0021530	0,0021530	0	-1	-1
386	387	0,0290012	0,0021530	0,0021530	0	-1	-1
387	388	0,0128316	0,0017088	0,0017088	18059892996	1,02339E+11	6,66674E-13
387	389	0,0266971	0,0017088	0,0017088	18037635829	1,02213E+11	6,67497E-13
1	390	0,0505322	0,0071091	0,0071091	0	-1	-1
390	391	0,0158387	0,0044784	0,0044784	1014164487	5746932093	1,18719E-11
390	392	0,0276905	0,0044784	0,0044784	0	-1	-1
392	393	0,0107231	0,0031052	0,0031052	3039704815	17224993949	3,96093E-12
392	394	0,0164667	0,0031052	0,0031052	0	-1	-1
394	395	0,0318748	0,0031052	0,0031052	0	-1	-1
395	396	0,0069867	0,0021530	0,0021530	9098048034	51555605524	1,32337E-12
395	397	0,0205806	0,0021530	0,0021530	0	-1	-1
397	398	0,0116065	0,0017088	0,0017088	18160145890	1,02907E+11	6,62994E-13
397	399	0,0288935	0,0017088	0,0017088	0	-1	-1
399	400	0,0151988	0,0011848	0,0011848	0	-1	-1
400	401	0,0078935	0,0011848	0,0011848	54236806101	3,07342E+11	2,21991E-13
399	402	0,0311236	0,0011848	0,0011848	0	-1	-1
402	403	0,0096399	0,0007464	0,0007464	0	-1	-1
403	404	0,0229101	0,0007464	0,0007464	2,15289E+11	1,21997E+12	5,59252E-14
402	405	0,0258175	0,0007464	0,0007464	0	-1	-1
405	406	0,0089827	0,0005924	0,0005924	4,30173E+11	2,43765E+12	2,79889E-14
405	407	0,0308628	0,0005924	0,0005924	0	-1	-1
407	408	0,0111693	0,0004702	0,0004702	8,52355E+11	4,83001E+12	1,41256E-14
407	409	0,0117840	0,0004702	0,0004702	8,52183E+11	4,82904E+12	1,41285E-14
402	410	0,0267833	0,0007464	0,0007464	0	-1	-1
410	411	0,0126706	0,0005924	0,0005924	4,29678E+11	2,43484E+12	2,80211E-14
410	412	0,0219722	0,0005924	0,0005924	0	-1	-1
412	413	0,0204470	0,0004108	0,0004108	0	-1	-1
413	414	0,0101884	0,0004108	0,0004108	1,2712E+12	7,20348E+12	9,4714E-15
412	415	0,0237252	0,0004108	0,0004108	0	-1	-1
415	416	0,0147819	0,0003260	0,0003260	2,53114E+12	1,43431E+13	4,75677E-15
415	417	0,0152908	0,0003260	0,0003260	2,53052E+12	1,43396E+13	4,75793E-15
412	418	0,0268940	0,0004108	0,0004108	1,273E+12	7,21368E+12	9,45801E-15
402	419	0,0531513	0,0007464	0,0007464	0	-1	-1
419	420	0,0264667	0,0005924	0,0005924	0	-1	-1
420	421	0,0187362	0,0005924	0,0005924	0	-1	-1
421	422	0,0164436	0,0005924	0,0005924	0	-1	-1
422	423	0,0260940	0,0005924	0,0005924	0	-1	-1

423	424	0,0204488	0,0005924	0,0005924	0	-1	-1
424	425	0,0109693	0,0004702	0,0004702	8,30404E+11	4,70562E+12	1,44991E-14
424	426	0,0120532	0,0004702	0,0004702	8,301E+11	4,7039E+12	1,45044E-14
419	427	0,0415448	0,0005924	0,0005924	0	-1	-1
427	428	0,0139813	0,0004108	0,0004108	0	-1	-1
428	429	0,0182805	0,0003260	0,0003260	0	-1	-1
429	430	0,0147981	0,0002261	0,0002261	0	-1	-1
430	431	0,0308340	0,0001794	0,0001794	0	-1	-1
431	432	0,0063002	0,0001794	0,0001794	0	-1	-1
432	433	0,0248378	0,0001794	0,0001794	1,40818E+13	7,97971E+13	8,55006E-16
430	434	0,0417217	0,0001794	0,0001794	0	-1	-1
434	435	0,0054533	0,0001794	0,0001794	1,42773E+13	8,09047E+13	8,43301E-16
429	436	0,0191665	0,0002261	0,0002261	7,42733E+12	4,20882E+13	1,62105E-15
429	437	0,0217586	0,0002261	0,0002261	7,41374E+12	4,20112E+13	1,62402E-15
428	438	0,0241960	0,0003260	0,0003260	2,5021E+12	1,41786E+13	4,81198E-15
427	439	0,0218873	0,0004108	0,0004108	0	-1	-1
439	440	0,0169131	0,0004108	0,0004108	0	-1	-1
440	441	0,0141377	0,0003260	0,0003260	2,49042E+12	1,41124E+13	4,83455E-15
440	442	0,0167049	0,0003260	0,0003260	2,48731E+12	1,40948E+13	4,8406E-15
427	443	0,0278889	0,0004108	0,0004108	0	-1	-1
443	444	0,0205622	0,0003260	0,0003260	0	-1	-1
444	445	0,0157576	0,0002261	0,0002261	0	-1	-1
445	446	0,0117143	0,0001567	0,0001567	2,19247E+13	1,2424E+14	5,49156E-16
445	447	0,0262492	0,0001567	0,0001567	0	-1	-1
447	448	0,0069537	0,0001244	0,0001244	0	-1	-1
448	449	0,0056934	0,0001244	0,0001244	4,24671E+13	2,40647E+14	2,83515E-16
447	450	0,0191104	0,0001244	0,0001244	0	-1	-1
450	451	0,0201405	0,0001244	0,0001244	4,09466E+13	2,32031E+14	2,94043E-16
445	452	0,0287475	0,0001567	0,0001567	2,15383E+13	1,2205E+14	5,59007E-16
444	453	0,0173469	0,0002261	0,0002261	0	-1	-1
453	454	0,0102847	0,0001567	0,0001567	0	-1	-1
454	455	0,0147910	0,0001567	0,0001567	0	-1	-1
455	456	0,0195546	0,0001567	0,0001567	2,11531E+13	1,19867E+14	5,69187E-16
453	457	0,0132390	0,0001567	0,0001567	0	-1	-1
457	458	0,0153233	0,0001567	0,0001567	0	-1	-1
458	459	0,0182556	0,0001244	0,0001244	0	-1	-1
459	460	0,0053579	0,0001244	0,0001244	4,16855E+13	2,36218E+14	2,88831E-16
458	461	0,0258291	0,0001244	0,0001244	0	-1	-1
461	462	0,0107489	0,0001244	0,0001244	4,09445E+13	2,32019E+14	2,94058E-16
453	463	0,0260485	0,0001567	0,0001567	0	-1	-1
463	464	0,0156415	0,0001567	0,0001567	0	-1	-1
464	465	0,0173311	0,0001567	0,0001567	2,08267E+13	1,18018E+14	5,78108E-16
444	466	0,0399274	0,0002261	0,0002261	0	-1	-1
466	467	0,0217879	0,0001794	0,0001794	0	-1	-1
467	468	0,0209917	0,0001424	0,0001424	2,7806E+13	1,57567E+14	4,33002E-16
467	469	0,0388204	0,0001424	0,0001424	0	-1	-1
469	470	0,0144308	0,0001130	0,0001130	5,32148E+13	3,0155E+14	2,26254E-16
469	471	0,0170638	0,0001130	0,0001130	5,29939E+13	3,00299E+14	2,27197E-16
466	472	0,0251966	0,0001794	0,0001794	0	-1	-1
472	473	0,0256435	0,0001424	0,0001424	0	-1	-1
473	474	0,0130065	0,0001130	0,0001130	5,40313E+13	3,06177E+14	2,22835E-16
473	475	0,0188784	0,0001130	0,0001130	0	-1	-1
475	476	0,0193899	0,0001130	0,0001130	0	-1	-1

476	477	0,0160380	0,0001130	0,0001130	5,05673E+13	2,86548E+14	2,381E-16
472	478	0,0408679	0,0001424	0,0001424	2,70544E+13	1,53308E+14	4,45033E-16
443	479	0,0384536	0,0003260	0,0003260	0	-1	-1
479	480	0,0128390	0,0002588	0,0002588	0	-1	-1
480	481	0,0177752	0,0002588	0,0002588	4,84944E+12	2,74802E+13	2,48277E-15
479	482	0,0241864	0,0002588	0,0002588	0	-1	-1
482	483	0,0216853	0,0002588	0,0002588	0	-1	-1
483	484	0,0006756	0,0002588	0,0002588	4,8008E+12	2,72045E+13	2,50793E-15
399	485	0,0439756	0,0011848	0,0011848	0	-1	-1
485	486	0,0110206	0,0009404	0,0009404	0	-1	-1
486	487	0,0212563	0,0009404	0,0009404	0	-1	-1
487	488	0,0164857	0,0009404	0,0009404	0	-1	-1
488	489	0,0183382	0,0007464	0,0007464	2,13852E+11	1,21183E+12	5,63011E-14
488	490	0,0356856	0,0007464	0,0007464	0	-1	-1
490	491	0,0201013	0,0005924	0,0005924	4,23939E+11	2,40232E+12	2,84005E-14
490	492	0,0246923	0,0005924	0,0005924	4,23429E+11	2,39943E+12	2,84347E-14
485	493	0,0385454	0,0009404	0,0009404	0	-1	-1
493	494	0,0195784	0,0006520	0,0006520	0	-1	-1
494	495	0,0118650	0,0006520	0,0006520	3,20146E+11	1,81416E+12	3,76081E-14
493	496	0,0205567	0,0006520	0,0006520	0	-1	-1
496	497	0,0241179	0,0006520	0,0006520	0	-1	-1
497	498	0,0173863	0,0006520	0,0006520	3,17827E+11	1,80102E+12	3,78824E-14
493	499	0,0285330	0,0006520	0,0006520	0	-1	-1
499	500	0,0285030	0,0005175	0,0005175	0	-1	-1
500	501	0,0243937	0,0004108	0,0004108	0	-1	-1
501	502	0,0095461	0,0004108	0,0004108	0	-1	-1
502	503	0,0118751	0,0003260	0,0003260	2,49415E+12	1,41335E+13	4,82732E-15
502	504	0,0119886	0,0003260	0,0003260	2,49401E+12	1,41327E+13	4,82759E-15
500	505	0,0305851	0,0004108	0,0004108	0	-1	-1
505	506	0,0198482	0,0003260	0,0003260	0	-1	-1
506	507	0,0093746	0,0002588	0,0002588	4,94681E+12	2,80319E+13	2,4339E-15
506	508	0,0159133	0,0002588	0,0002588	0	-1	-1
508	509	0,0085508	0,0002588	0,0002588	4,90074E+12	2,77709E+13	2,45678E-15
505	510	0,0311930	0,0003260	0,0003260	0	-1	-1
510	511	0,0061625	0,0003260	0,0003260	2,4665E+12	1,39769E+13	4,88143E-15
499	512	0,0318414	0,0005175	0,0005175	0	-1	-1
512	513	0,0297493	0,0004108	0,0004108	1,25501E+12	7,11172E+12	9,59361E-15
512	514	0,0326171	0,0004108	0,0004108	1,25363E+12	7,10391E+12	9,60416E-15
395	515	0,0295659	0,0021530	0,0021530	0	-1	-1
515	516	0,0089274	0,0017088	0,0017088	18152998559	1,02867E+11	6,63255E-13
515	517	0,0241717	0,0017088	0,0017088	0	-1	-1
517	518	0,0164671	0,0017088	0,0017088	18102095104	1,02579E+11	6,6512E-13
392	519	0,0301907	0,0031052	0,0031052	0	-1	-1
519	520	0,0251739	0,0031052	0,0031052	0	-1	-1
520	521	0,0126094	0,0021530	0,0021530	0	-1	-1
521	522	0,0265054	0,0021530	0,0021530	0	-1	-1
522	523	0,0186083	0,0021530	0,0021530	0	-1	-1
523	524	0,0092630	0,0021530	0,0021530	9056724645	51321439657	1,32941E-12
520	525	0,0151379	0,0021530	0,0021530	0	-1	-1
525	526	0,0173784	0,0017088	0,0017088	0	-1	-1
526	527	0,0173073	0,0017088	0,0017088	18123829106	1,02702E+11	6,64323E-13
525	528	0,0191751	0,0017088	0,0017088	18148726912	1,02843E+11	6,63411E-13
520	529	0,0383867	0,0021530	0,0021530	0	-1	-1

529	530	0,0108741	0,0021530	0,0021530	0	-1	-1
530	531	0,0191328	0,0021530	0,0021530	0	-1	-1
531	532	0,0185697	0,0014928	0,0014928	0	-1	-1
532	533	0,0140938	0,0011848	0,0011848	0	-1	-1
533	534	0,0275817	0,0011848	0,0011848	0	-1	-1
534	535	0,0191946	0,0011848	0,0011848	0	-1	-1
535	536	0,0103859	0,0008215	0,0008215	0	-1	-1
536	537	0,0173741	0,0008215	0,0008215	0	-1	-1
537	538	0,0169718	0,0008215	0,0008215	0	-1	-1
538	539	0,0064986	0,0008215	0,0008215	1,5989E+11	9,06043E+11	7,53022E-14
535	540	0,0178116	0,0008215	0,0008215	0	-1	-1
540	541	0,0172288	0,0008215	0,0008215	0	-1	-1
541	542	0,0266853	0,0008215	0,0008215	0	-1	-1
542	543	0,0192644	0,0005696	0,0005696	0	-1	-1
543	544	0,0251730	0,0005696	0,0005696	0	-1	-1
544	545	0,0220174	0,0005696	0,0005696	4,70083E+11	2,66381E+12	2,56126E-14
542	546	0,0204000	0,0005696	0,0005696	0	-1	-1
546	547	0,0090683	0,0004521	0,0004521	0	-1	-1
547	548	0,0084356	0,0004521	0,0004521	9,46408E+11	5,36298E+12	1,27219E-14
546	549	0,0193029	0,0004521	0,0004521	9,45818E+11	5,35964E+12	1,27298E-14
542	550	0,0255405	0,0005696	0,0005696	0	-1	-1
550	551	0,0222352	0,0005696	0,0005696	0	-1	-1
551	552	0,0133817	0,0003949	0,0003949	1,41001E+12	7,99004E+12	8,53901E-15
551	553	0,0318779	0,0003949	0,0003949	0	-1	-1
553	554	0,0084974	0,0003949	0,0003949	0	-1	-1
554	555	0,0085293	0,0003135	0,0003135	2,77755E+12	1,57395E+13	4,33478E-15
554	556	0,0163547	0,0003135	0,0003135	0	-1	-1
556	557	0,0121471	0,0003135	0,0003135	2,74924E+12	1,5579E+13	4,37942E-15
551	558	0,0321878	0,0003949	0,0003949	0	-1	-1
558	559	0,0201485	0,0003135	0,0003135	0	-1	-1
559	560	0,0099405	0,0002488	0,0002488	5,50508E+12	3,11954E+13	2,18708E-15
559	561	0,0268839	0,0002488	0,0002488	5,44455E+12	3,08525E+13	2,2114E-15
558	562	0,0237773	0,0003135	0,0003135	0	-1	-1
562	563	0,0092218	0,0002173	0,0002173	8,23889E+12	4,6687E+13	1,46137E-15
562	564	0,0348289	0,0002173	0,0002173	0	-1	-1
564	565	0,0180456	0,0001369	0,0001369	3,16245E+13	1,79205E+14	3,8072E-16
564	566	0,0218358	0,0001369	0,0001369	0	-1	-1
566	567	0,0066996	0,0001087	0,0001087	6,22963E+13	3,53012E+14	1,93271E-16
566	568	0,0175676	0,0001087	0,0001087	6,12297E+13	3,46968E+14	1,96638E-16
564	569	0,0234407	0,0001369	0,0001369	3,14144E+13	1,78015E+14	3,83266E-16
564	570	0,0417243	0,0001369	0,0001369	0	-1	-1
570	571	0,0118723	0,0001369	0,0001369	3,02399E+13	1,7136E+14	3,98151E-16
562	572	0,0414099	0,0002173	0,0002173	0	-1	-1
572	573	0,0193919	0,0002173	0,0002173	7,92251E+12	4,48943E+13	1,51973E-15
535	574	0,0284124	0,0008215	0,0008215	0	-1	-1
574	575	0,0122467	0,0006520	0,0006520	0	-1	-1
575	576	0,0071166	0,0005175	0,0005175	6,3909E+11	3,62151E+12	1,88394E-14
575	577	0,0139157	0,0005175	0,0005175	0	-1	-1
577	578	0,0092832	0,0003588	0,0003588	0	-1	-1
578	579	0,0152118	0,0002848	0,0002848	3,77978E+12	2,14188E+13	3,18539E-15
578	580	0,0214703	0,0002848	0,0002848	3,76676E+12	2,1345E+13	3,1964E-15
577	581	0,0172223	0,0003588	0,0003588	0	-1	-1
581	582	0,0177613	0,0002848	0,0002848	3,76137E+12	2,13144E+13	3,20098E-15

581	583	0,0254404	0,0002848	0,0002848	0	-1	-1
583	584	0,0267632	0,0002261	0,0002261	0	-1	-1
584	585	0,0130307	0,0001794	0,0001794	0	-1	-1
585	586	0,0137790	0,0001424	0,0001424	0	-1	-1
586	587	0,0051937	0,0001130	0,0001130	5,67625E+13	3,21654E+14	2,12113E-16
586	588	0,0154696	0,0001130	0,0001130	5,59006E+13	3,1677E+14	2,15383E-16
585	589	0,0304223	0,0001424	0,0001424	2,80451E+13	1,58922E+14	4,29311E-16
584	590	0,0307112	0,0001794	0,0001794	0	-1	-1
590	591	0,0152949	0,0001424	0,0001424	2,80815E+13	1,59129E+14	4,28754E-16
590	592	0,0195861	0,0001424	0,0001424	2,79387E+13	1,58319E+14	4,30946E-16
583	593	0,0312173	0,0002261	0,0002261	0	-1	-1
593	594	0,0239128	0,0002261	0,0002261	0	-1	-1
594	595	0,0271634	0,0001567	0,0001567	0	-1	-1
595	596	0,0165532	0,0001567	0,0001567	2,06138E+13	1,16812E+14	5,84078E-16
594	597	0,0285563	0,0001567	0,0001567	0	-1	-1
597	598	0,0140917	0,0001567	0,0001567	0	-1	-1
598	599	0,0086866	0,0001567	0,0001567	0	-1	-1
599	600	0,0085332	0,0001567	0,0001567	2,02475E+13	1,14736E+14	5,94645E-16
594	601	0,0306792	0,0001567	0,0001567	0	-1	-1
601	602	0,0356922	0,0001567	0,0001567	2,01E+13	1,139E+14	5,99009E-16
577	603	0,0203616	0,0003588	0,0003588	0	-1	-1
603	604	0,0183083	0,0002488	0,0002488	5,62431E+12	3,18711E+13	2,14072E-15
603	605	0,0258883	0,0002488	0,0002488	0	-1	-1
605	606	0,0185193	0,0001975	0,0001975	1,10278E+13	6,24906E+13	1,0918E-15
605	607	0,0202008	0,0001975	0,0001975	0	-1	-1
607	608	0,0208905	0,0001975	0,0001975	0	-1	-1
608	609	0,0200272	0,0001975	0,0001975	0	-1	-1
609	610	0,0070677	0,0001975	0,0001975	1,05807E+13	5,99573E+13	1,13793E-15
603	611	0,0337593	0,0002488	0,0002488	0	-1	-1
611	612	0,0064915	0,0001975	0,0001975	1,10798E+13	6,27855E+13	1,08667E-15
611	613	0,0213634	0,0001975	0,0001975	0	-1	-1
613	614	0,0144198	0,0001567	0,0001567	2,15648E+13	1,222E+14	5,58321E-16
613	615	0,0289894	0,0001567	0,0001567	0	-1	-1
615	616	0,0123631	0,0001244	0,0001244	4,17621E+13	2,36652E+14	2,88301E-16
615	617	0,0164173	0,0001244	0,0001244	4,15304E+13	2,35339E+14	2,8991E-16
574	618	0,0142064	0,0006520	0,0006520	3,20076E+11	1,81376E+12	3,76163E-14
532	619	0,0307422	0,0011848	0,0011848	0	-1	-1
619	620	0,0203303	0,0009404	0,0009404	0	-1	-1
620	621	0,0107083	0,0009404	0,0009404	0	-1	-1
621	622	0,0184146	0,0009404	0,0009404	0	-1	-1
622	623	0,0300215	0,0009404	0,0009404	0	-1	-1
623	624	0,0166145	0,0009404	0,0009404	0	-1	-1
624	625	0,0095050	0,0009404	0,0009404	1,0619E+11	6,01744E+11	1,13382E-13
619	626	0,0285800	0,0009404	0,0009404	0	-1	-1
626	627	0,0131897	0,0006520	0,0006520	3,21615E+11	1,82249E+12	3,74363E-14
626	628	0,0192793	0,0006520	0,0006520	0	-1	-1
628	629	0,0082741	0,0006520	0,0006520	0	-1	-1
629	630	0,0262946	0,0005175	0,0005175	0	-1	-1
630	631	0,0189428	0,0005175	0,0005175	6,32423E+11	3,58373E+12	1,9038E-14
629	632	0,0264357	0,0005175	0,0005175	0	-1	-1
632	633	0,0215242	0,0004108	0,0004108	1,26167E+12	7,14948E+12	9,54294E-15
632	634	0,0496755	0,0004108	0,0004108	0	-1	-1
634	635	0,0180038	0,0003260	0,0003260	0	-1	-1

635	636	0,0117005	0,0002054	0,0002054	9,80783E+12	5,55777E+13	1,2276E-15
635	637	0,0134925	0,0002054	0,0002054	9,79405E+12	5,54996E+13	1,22933E-15
635	638	0,0228387	0,0002054	0,0002054	9,72215E+12	5,50922E+13	1,23842E-15
635	639	0,0253675	0,0002054	0,0002054	9,70269E+12	5,49819E+13	1,2409E-15
634	640	0,0335204	0,0003260	0,0003260	2,45566E+12	1,39154E+13	4,90298E-15
626	641	0,0245157	0,0006520	0,0006520	0	-1	-1
641	642	0,0194357	0,0006520	0,0006520	0	-1	-1
642	643	0,0207781	0,0006520	0,0006520	0	-1	-1
643	644	0,0253037	0,0005175	0,0005175	0	-1	-1
644	645	0,0148067	0,0004108	0,0004108	1,25407E+12	7,10642E+12	9,60076E-15
644	646	0,0255309	0,0004108	0,0004108	0	-1	-1
646	647	0,0151216	0,0003260	0,0003260	2,47951E+12	1,40506E+13	4,85582E-15
646	648	0,0235777	0,0003260	0,0003260	0	-1	-1
648	649	0,0091886	0,0002261	0,0002261	0	-1	-1
649	650	0,0184416	0,0002261	0,0002261	0	-1	-1
650	651	0,0055718	0,0002261	0,0002261	7,23376E+12	4,09913E+13	1,66443E-15
648	652	0,0189040	0,0002261	0,0002261	0	-1	-1
652	653	0,0117107	0,0002261	0,0002261	7,24732E+12	4,10682E+13	1,66131E-15
648	654	0,0240620	0,0002261	0,0002261	7,28167E+12	4,12628E+13	1,65348E-15
643	655	0,0294961	0,0005175	0,0005175	6,29797E+11	3,56885E+12	1,91174E-14
531	656	0,0205523	0,0014928	0,0014928	27110836705	1,53628E+11	4,44105E-13
531	657	0,0461842	0,0014928	0,0014928	0	-1	-1
657	658	0,0092275	0,0009404	0,0009404	1,07999E+11	6,11996E+11	1,11483E-13
657	659	0,0252842	0,0009404	0,0009404	1,07718E+11	6,10403E+11	1,11774E-13
657	660	0,0279774	0,0009404	0,0009404	0	-1	-1
660	661	0,0129921	0,0009404	0,0009404	1,07444E+11	6,08848E+11	1,12059E-13
657	662	0,0307411	0,0009404	0,0009404	0	-1	-1
662	663	0,0151207	0,0007464	0,0007464	0	-1	-1
663	664	0,0169367	0,0005924	0,0005924	0	-1	-1
664	665	0,0254215	0,0005924	0,0005924	0	-1	-1
665	666	0,0208648	0,0005924	0,0005924	0	-1	-1
666	667	0,0079630	0,0004702	0,0004702	8,42034E+11	4,77152E+12	1,42988E-14
666	668	0,0149560	0,0004702	0,0004702	8,40075E+11	4,76043E+12	1,43321E-14
663	669	0,0171458	0,0005924	0,0005924	0	-1	-1
669	670	0,0206251	0,0005924	0,0005924	4,2496E+11	2,40811E+12	2,83322E-14
662	671	0,0213052	0,0007464	0,0007464	0	-1	-1
671	672	0,0204663	0,0007464	0,0007464	0	-1	-1
672	673	0,0227951	0,0005175	0,0005175	0	-1	-1
673	674	0,0154342	0,0005175	0,0005175	0	-1	-1
674	675	0,0182391	0,0004108	0,0004108	1,25706E+12	7,12335E+12	9,57795E-15
674	676	0,0199295	0,0004108	0,0004108	0	-1	-1
676	677	0,0160213	0,0004108	0,0004108	0	-1	-1
677	678	0,0117544	0,0003260	0,0003260	2,48285E+12	1,40695E+13	4,84929E-15
677	679	0,0241378	0,0003260	0,0003260	0	-1	-1
679	680	0,0168958	0,0003260	0,0003260	0	-1	-1
680	681	0,0228869	0,0003260	0,0003260	0	-1	-1
681	682	0,0214917	0,0002588	0,0002588	4,77368E+12	2,70509E+13	2,52218E-15
681	683	0,0249872	0,0002588	0,0002588	0	-1	-1
683	684	0,0192504	0,0001630	0,0001630	0	-1	-1
684	685	0,0119228	0,0001630	0,0001630	0	-1	-1
685	686	0,0259694	0,0001630	0,0001630	1,79443E+13	1,01684E+14	6,70968E-16
683	687	0,0197670	0,0001630	0,0001630	1,86688E+13	1,0579E+14	6,44928E-16
683	688	0,0215966	0,0001630	0,0001630	1,86334E+13	1,05589E+14	6,46156E-16

683	689	0,0222259	0,0001630	0,0001630	1,86212E+13	1,0552E+14	6,46579E-16
672	690	0,0280700	0,0005175	0,0005175	6,34854E+11	3,59751E+12	1,89651E-14
672	691	0,0302342	0,0005175	0,0005175	0	-1	-1
691	692	0,0246585	0,0005175	0,0005175	0	-1	-1
692	693	0,0149053	0,0005175	0,0005175	0	-1	-1
693	694	0,0110261	0,0005175	0,0005175	6,24788E+11	3,54047E+12	1,92706E-14
390	695	0,0310352	0,0044784	0,0044784	0	-1	-1
695	696	0,0038160	0,0035545	0,0035545	0	-1	-1
696	697	0,0080305	0,0028212	0,0028212	0	-1	-1
697	698	0,0164814	0,0022392	0,0022392	0	-1	-1
698	699	0,0295792	0,0017773	0,0017773	0	-1	-1
699	700	0,0339921	0,0017773	0,0017773	0	-1	-1
700	701	0,0065524	0,0017773	0,0017773	16094649472	91203013674	7,48079E-13
698	702	0,0395212	0,0017773	0,0017773	16136633727	91440924455	7,46133E-13
697	703	0,0241893	0,0022392	0,0022392	0	-1	-1
703	704	0,0314894	0,0022392	0,0022392	0	-1	-1
704	705	0,0239925	0,0017773	0,0017773	0	-1	-1
705	706	0,0141261	0,0014106	0,0014106	0	-1	-1
706	707	0,0162763	0,0014106	0,0014106	0	-1	-1
707	708	0,0126537	0,0014106	0,0014106	0	-1	-1
708	709	0,0150566	0,0011196	0,0011196	0	-1	-1
709	710	0,0131689	0,0008886	0,0008886	1,27775E+11	7,2406E+11	9,42285E-14
709	711	0,0308488	0,0008886	0,0008886	0	-1	-1
711	712	0,0146494	0,0005598	0,0005598	0	-1	-1
712	713	0,0348602	0,0005598	0,0005598	5,02648E+11	2,84834E+12	2,39533E-14
711	714	0,0152524	0,0005598	0,0005598	5,07423E+11	2,8754E+12	2,37279E-14
711	715	0,0169020	0,0005598	0,0005598	5,07193E+11	2,87409E+12	2,37386E-14
711	716	0,0251746	0,0005598	0,0005598	0	-1	-1
716	717	0,0114069	0,0003881	0,0003881	1,51124E+12	8,5637E+12	7,96701E-15
716	718	0,0128465	0,0003881	0,0003881	1,51037E+12	8,55878E+12	7,97159E-15
716	719	0,0219778	0,0003881	0,0003881	0	-1	-1
719	720	0,0102320	0,0003081	0,0003081	0	-1	-1
720	721	0,0097842	0,0002445	0,0002445	5,9509E+12	3,37218E+13	2,02323E-15
720	722	0,0310635	0,0002445	0,0002445	0	-1	-1
722	723	0,0158563	0,0002445	0,0002445	5,8087E+12	3,2916E+13	2,07276E-15
719	724	0,0242727	0,0003081	0,0003081	0	-1	-1
724	725	0,0152134	0,0003081	0,0003081	2,94973E+12	1,67151E+13	4,08175E-15
708	726	0,0175264	0,0011196	0,0011196	0	-1	-1
726	727	0,0060911	0,0008886	0,0008886	1,27888E+11	7,24696E+11	9,41457E-14
726	728	0,0086565	0,0008886	0,0008886	1,27831E+11	7,24377E+11	9,41872E-14
705	729	0,0331896	0,0014106	0,0014106	0	-1	-1
729	730	0,0140053	0,0008886	0,0008886	1,28156E+11	7,26215E+11	9,39488E-14
729	731	0,0210523	0,0008886	0,0008886	0	-1	-1
731	732	0,0171651	0,0007053	0,0007053	0	-1	-1
732	733	0,0090205	0,0005598	0,0005598	5,08848E+11	2,88347E+12	2,36614E-14
732	734	0,0259470	0,0005598	0,0005598	0	-1	-1
734	735	0,0128560	0,0005598	0,0005598	0	-1	-1
735	736	0,0214933	0,0005598	0,0005598	0	-1	-1
736	737	0,0124118	0,0004443	0,0004443	9,99043E+11	5,66124E+12	1,20516E-14
736	738	0,0141158	0,0004443	0,0004443	0	-1	-1
738	739	0,0086221	0,0003527	0,0003527	0	-1	-1
739	740	0,0344610	0,0003527	0,0003527	1,95876E+12	1,10996E+13	6,14678E-15
738	741	0,0201931	0,0003527	0,0003527	0	-1	-1

741	742	0,0192966	0,0003527	0,0003527	0	-1	-1
742	743	0,0180927	0,0002799	0,0002799	3,88353E+12	2,20067E+13	3,10029E-15
742	744	0,0209187	0,0002799	0,0002799	0	-1	-1
744	745	0,0097111	0,0002222	0,0002222	7,69989E+12	4,36327E+13	1,56367E-15
744	746	0,0199596	0,0002222	0,0002222	0	-1	-1
746	747	0,0236717	0,0002222	0,0002222	0	-1	-1
747	748	0,0094960	0,0002222	0,0002222	0	-1	-1
748	749	0,0031483	0,0001763	0,0001763	1,48673E+13	8,42478E+13	8,09838E-16
748	750	0,0234816	0,0001763	0,0001763	0	-1	-1
750	751	0,0141136	0,0001763	0,0001763	1,43795E+13	8,14838E+13	8,37308E-16
731	752	0,0182930	0,0007053	0,0007053	2,5499E+11	1,44494E+12	4,72178E-14
729	753	0,0226336	0,0008886	0,0008886	0	-1	-1
753	754	0,0317032	0,0008886	0,0008886	0	-1	-1
754	755	0,0192641	0,0007053	0,0007053	0	-1	-1
755	756	0,0199863	0,0007053	0,0007053	2,5237E+11	1,43009E+12	4,77081E-14
754	757	0,0224347	0,0007053	0,0007053	0	-1	-1
757	758	0,0155954	0,0005598	0,0005598	0	-1	-1
758	759	0,0134909	0,0005598	0,0005598	0	-1	-1
759	760	0,0167971	0,0005598	0,0005598	0	-1	-1
760	761	0,0122675	0,0004443	0,0004443	0	-1	-1
761	762	0,0239758	0,0004443	0,0004443	9,8768E+11	5,59685E+12	1,21903E-14
760	763	0,0133938	0,0004443	0,0004443	0	-1	-1
763	764	0,0074911	0,0003081	0,0003081	2,97573E+12	1,68625E+13	4,04609E-15
763	765	0,0107600	0,0003081	0,0003081	0	-1	-1
765	766	0,0115905	0,0002445	0,0002445	0	-1	-1
766	767	0,0486751	0,0002445	0,0002445	0	-1	-1
767	768	0,0198171	0,0001941	0,0001941	0	-1	-1
768	769	0,0154200	0,0001223	0,0001223	4,39765E+13	2,492E+14	2,73784E-16
768	770	0,0157219	0,0001223	0,0001223	0	-1	-1
770	771	0,0120336	0,0001223	0,0001223	0	-1	-1
771	772	0,0182772	0,0001223	0,0001223	4,2101E+13	2,38572E+14	2,85981E-16
768	773	0,0206023	0,0001223	0,0001223	0	-1	-1
773	774	0,0194049	0,0001223	0,0001223	4,24701E+13	2,40664E+14	2,83495E-16
768	775	0,0291501	0,0001223	0,0001223	4,31353E+13	2,44433E+14	2,79123E-16
767	776	0,0219755	0,0001941	0,0001941	0	-1	-1
776	777	0,0156484	0,0001941	0,0001941	0	-1	-1
777	778	0,0166207	0,0001941	0,0001941	1,08981E+13	6,1756E+13	1,10478E-15
765	779	0,0149931	0,0002445	0,0002445	5,88412E+12	3,33433E+13	2,0462E-15
763	780	0,0286794	0,0003081	0,0003081	0	-1	-1
780	781	0,0109148	0,0003081	0,0003081	2,92695E+12	1,6586E+13	4,11352E-15
757	782	0,0158159	0,0005598	0,0005598	0	-1	-1
782	783	0,0221045	0,0005598	0,0005598	0	-1	-1
783	784	0,0087294	0,0004443	0,0004443	9,99563E+11	5,66419E+12	1,20453E-14
783	785	0,0105231	0,0004443	0,0004443	9,98933E+11	5,66062E+12	1,20529E-14
729	786	0,0382428	0,0008886	0,0008886	0	-1	-1
786	787	0,0234041	0,0007053	0,0007053	0	-1	-1
787	788	0,0101003	0,0004125	0,0004125	1,26499E+12	7,16826E+12	9,51794E-15
787	789	0,0196068	0,0004125	0,0004125	1,26049E+12	7,14278E+12	9,55188E-15
787	790	0,0207344	0,0004125	0,0004125	1,25996E+12	7,13976E+12	9,55593E-15
787	791	0,0273172	0,0004125	0,0004125	0	-1	-1
791	792	0,0222435	0,0002860	0,0002860	0	-1	-1
792	793	0,0161986	0,0002270	0,0002270	0	-1	-1
793	794	0,0253424	0,0002270	0,0002270	0	-1	-1

794	795	0,0242375	0,0002270	0,0002270	0	-1	-1
795	796	0,0417327	0,0002270	0,0002270	0	-1	-1
796	797	0,0121227	0,0001430	0,0001430	2,7186E+13	1,54054E+14	4,42878E-16
796	798	0,0198669	0,0001430	0,0001430	0	-1	-1
798	799	0,0242317	0,0001430	0,0001430	0	-1	-1
799	800	0,0138975	0,0001430	0,0001430	2,56841E+13	1,45543E+14	4,68775E-16
796	801	0,0281048	0,0001430	0,0001430	0	-1	-1
801	802	0,0217072	0,0001430	0,0001430	0	-1	-1
802	803	0,0092901	0,0001430	0,0001430	0	-1	-1
803	804	0,0256028	0,0001430	0,0001430	0	-1	-1
804	805	0,0013501	0,0001135	0,0001135	0	-1	-1
805	806	0,0232686	0,0001135	0,0001135	0	-1	-1
806	807	0,0198378	0,0001135	0,0001135	4,59519E+13	2,60394E+14	2,62015E-16
804	808	0,0161129	0,0001135	0,0001135	4,82901E+13	2,73644E+14	2,49328E-16
796	809	0,0369857	0,0001430	0,0001430	0	-1	-1
809	810	0,0287686	0,0001430	0,0001430	0	-1	-1
810	811	0,0085508	0,0000901	0,0000901	9,99429E+13	5,66343E+14	1,20469E-16
810	812	0,0102835	0,0000901	0,0000901	0	-1	-1
812	813	0,0366965	0,0000901	0,0000901	9,19543E+13	5,21074E+14	1,30935E-16
810	814	0,0201353	0,0000901	0,0000901	9,75347E+13	5,52697E+14	1,23444E-16
810	815	0,0395823	0,0000901	0,0000901	0	-1	-1
815	816	0,0154253	0,0000715	0,0000715	1,78904E+14	1,01379E+15	6,7299E-17
815	817	0,0214244	0,0000715	0,0000715	0	-1	-1
817	818	0,0139810	0,0000715	0,0000715	1,68438E+14	9,54483E+14	7,14806E-17
792	819	0,0275884	0,0002270	0,0002270	0	-1	-1
819	820	0,0156364	0,0001802	0,0001802	1,44124E+13	8,16705E+13	8,35394E-16
819	821	0,0239275	0,0001802	0,0001802	1,43047E+13	8,106E+13	8,41685E-16
791	822	0,0247463	0,0002860	0,0002860	3,7199E+12	2,10794E+13	3,23666E-15
791	823	0,0268997	0,0002860	0,0002860	0	-1	-1
823	824	0,0194037	0,0002270	0,0002270	0	-1	-1
824	825	0,0101342	0,0002270	0,0002270	0	-1	-1
825	826	0,0149060	0,0002270	0,0002270	0	-1	-1
826	827	0,0147450	0,0002270	0,0002270	7,12581E+12	4,03796E+13	1,68964E-15
823	828	0,0385688	0,0002270	0,0002270	0	-1	-1
828	829	0,0121426	0,0001574	0,0001574	0	-1	-1
829	830	0,0188916	0,0001574	0,0001574	0	-1	-1
830	831	0,0264002	0,0001574	0,0001574	2,04151E+13	1,15685E+14	5,89764E-16
828	832	0,0192203	0,0001574	0,0001574	2,12676E+13	1,20516E+14	5,66123E-16
828	833	0,0297272	0,0001574	0,0001574	0	-1	-1
833	834	0,0227248	0,0001249	0,0001249	4,07889E+13	2,31137E+14	2,9518E-16
833	835	0,0301599	0,0001249	0,0001249	0	-1	-1
835	836	0,0238410	0,0000866	0,0000866	0	-1	-1
836	837	0,0136118	0,0000866	0,0000866	0	-1	-1
837	838	0,0115089	0,0000866	0,0000866	1,09204E+14	6,18822E+14	1,10253E-16
835	839	0,0296686	0,0000866	0,0000866	1,13897E+14	6,45414E+14	1,05711E-16
835	840	0,0368651	0,0000866	0,0000866	1,12146E+14	6,35495E+14	1,0736E-16
787	841	0,0337872	0,0004125	0,0004125	0	-1	-1
841	842	0,0140365	0,0002860	0,0002860	3,73263E+12	2,11516E+13	3,22562E-15
841	843	0,0279190	0,0002860	0,0002860	0	-1	-1
843	844	0,0141681	0,0002270	0,0002270	7,33541E+12	4,15673E+13	1,64136E-15
843	845	0,0361738	0,0002270	0,0002270	0	-1	-1
845	846	0,0098241	0,0001802	0,0001802	0	-1	-1
846	847	0,0168750	0,0001802	0,0001802	0	-1	-1

847	848	0,0085494	0,0001802	0,0001802	0	-1	-1
848	849	0,0233288	0,0001802	0,0001802	0	-1	-1
849	850	0,0209418	0,0001430	0,0001430	2,668E+13	1,51187E+14	4,51276E-16
849	851	0,0254170	0,0001430	0,0001430	0	-1	-1
851	852	0,0071587	0,0001430	0,0001430	0	-1	-1
852	853	0,0138910	0,0001430	0,0001430	2,58444E+13	1,46452E+14	4,65868E-16
845	854	0,0183076	0,0001802	0,0001802	0	-1	-1
854	855	0,0193354	0,0001430	0,0001430	2,7779E+13	1,57415E+14	4,33423E-16
854	856	0,0217467	0,0001430	0,0001430	0	-1	-1
856	857	0,0200427	0,0001430	0,0001430	0	-1	-1
857	858	0,0145762	0,0001430	0,0001430	2,65667E+13	1,50545E+14	4,53201E-16
841	859	0,0350247	0,0002860	0,0002860	0	-1	-1
859	860	0,0241999	0,0002860	0,0002860	0	-1	-1
860	861	0,0113644	0,0002270	0,0002270	7,22175E+12	4,09232E+13	1,6672E-15
860	862	0,0211520	0,0002270	0,0002270	7,17128E+12	4,06373E+13	1,67893E-15
786	863	0,0248771	0,0007053	0,0007053	0	-1	-1
863	864	0,0148372	0,0005598	0,0005598	5,05675E+11	2,86549E+12	2,38099E-14
863	865	0,0343158	0,0005598	0,0005598	0	-1	-1
865	866	0,0206695	0,0003527	0,0003527	0	-1	-1
866	867	0,0240441	0,0002799	0,0002799	0	-1	-1
867	868	0,0066385	0,0002222	0,0002222	7,82964E+12	4,4368E+13	1,53775E-15
867	869	0,0355346	0,0002222	0,0002222	0	-1	-1
869	870	0,0189694	0,0001540	0,0001540	2,25406E+13	1,2773E+14	5,34151E-16
869	871	0,0200460	0,0001540	0,0001540	0	-1	-1
871	872	0,0172670	0,0001540	0,0001540	0	-1	-1
872	873	0,0241439	0,0001223	0,0001223	4,27099E+13	2,42023E+14	2,81903E-16
872	874	0,0254622	0,0001223	0,0001223	0	-1	-1
874	875	0,0170427	0,0001223	0,0001223	0	-1	-1
875	876	0,0170563	0,0001223	0,0001223	4,054E+13	2,29727E+14	2,96992E-16
869	877	0,0247687	0,0001540	0,0001540	0	-1	-1
877	878	0,0258227	0,0001068	0,0001068	0	-1	-1
878	879	0,0098177	0,0001068	0,0001068	0	-1	-1
879	880	0,0095155	0,0001068	0,0001068	6,24484E+13	3,53874E+14	1,928E-16
877	881	0,0312781	0,0001068	0,0001068	0	-1	-1
881	882	0,0215647	0,0000848	0,0000848	1,221E+14	6,919E+14	9,86082E-17
881	883	0,0238153	0,0000848	0,0000848	0	-1	-1
883	884	0,0276947	0,0000848	0,0000848	0	-1	-1
884	885	0,0134846	0,0000673	0,0000673	2,19317E+14	1,24279E+15	5,48981E-17
884	886	0,0149081	0,0000673	0,0000673	2,18366E+14	1,23741E+15	5,51371E-17
877	887	0,0322846	0,0001068	0,0001068	0	-1	-1
887	888	0,0143208	0,0000848	0,0000848	1,23809E+14	7,01582E+14	9,72474E-17
887	889	0,0182835	0,0000848	0,0000848	0	-1	-1
889	890	0,0192675	0,0000673	0,0000673	0	-1	-1
890	891	0,0090727	0,0000673	0,0000673	2,26586E+14	1,28399E+15	5,31369E-17
889	892	0,0203334	0,0000673	0,0000673	0	-1	-1
892	893	0,0127934	0,0000534	0,0000534	4,42335E+14	2,50656E+15	2,72194E-17
892	894	0,0183217	0,0000534	0,0000534	4,33029E+14	2,45383E+15	2,78043E-17
866	895	0,0291599	0,0002799	0,0002799	0	-1	-1
895	896	0,0153124	0,0001763	0,0001763	1,54714E+13	8,76715E+13	7,78213E-16
895	897	0,0166224	0,0001763	0,0001763	1,54529E+13	8,75663E+13	7,79147E-16
895	898	0,0307133	0,0001763	0,0001763	0	-1	-1
898	899	0,0123777	0,0001400	0,0001400	0	-1	-1
899	900	0,0182592	0,0001400	0,0001400	2,94136E+13	1,66677E+14	4,09337E-16

898	901	0,0219764	0,0001400	0,0001400	0	-1	-1
901	902	0,0059755	0,0001400	0,0001400	2,95094E+13	1,6722E+14	4,08008E-16
895	903	0,0380157	0,0001763	0,0001763	0	-1	-1
903	904	0,0171623	0,0001031	0,0001031	7,3672E+13	4,17475E+14	1,63428E-16
903	905	0,0199972	0,0001031	0,0001031	0	-1	-1
905	906	0,0097150	0,0001031	0,0001031	7,21526E+13	4,08865E+14	1,66869E-16
903	907	0,0258845	0,0001031	0,0001031	0	-1	-1
907	908	0,0189529	0,0001031	0,0001031	7,03215E+13	3,98488E+14	1,71215E-16
903	909	0,0276891	0,0001031	0,0001031	0	-1	-1
909	910	0,0125982	0,0000818	0,0000818	0	-1	-1
910	911	0,0107930	0,0000818	0,0000818	1,37659E+14	7,80069E+14	8,74628E-17
909	912	0,0233095	0,0000818	0,0000818	1,37684E+14	7,8021E+14	8,7447E-17
903	913	0,0424346	0,0001031	0,0001031	0	-1	-1
913	914	0,0285670	0,0000818	0,0000818	0	-1	-1
914	915	0,0060418	0,0000650	0,0000650	0	-1	-1
915	916	0,0222402	0,0000650	0,0000650	0	-1	-1
916	917	0,0190044	0,0000650	0,0000650	2,2867E+14	1,2958E+15	5,26526E-17
914	918	0,0206191	0,0000650	0,0000650	2,4917E+14	1,41196E+15	4,83208E-17
913	919	0,0334169	0,0000818	0,0000818	0	-1	-1
919	920	0,0147297	0,0000650	0,0000650	0	-1	-1
920	921	0,0316894	0,0000650	0,0000650	0	-1	-1
921	922	0,0176165	0,0000650	0,0000650	0	-1	-1
922	923	0,0100529	0,0000450	0,0000450	6,05069E+14	3,42873E+15	1,98987E-17
922	924	0,0177106	0,0000450	0,0000450	0	-1	-1
924	925	0,0127826	0,0000450	0,0000450	5,37083E+14	3,04347E+15	2,24175E-17
922	926	0,0280534	0,0000450	0,0000450	5,45199E+14	3,08946E+15	2,20838E-17
919	927	0,0266250	0,0000650	0,0000650	0	-1	-1
927	928	0,0231680	0,0000650	0,0000650	2,23784E+14	1,26811E+15	5,38022E-17
865	929	0,0252479	0,0003527	0,0003527	0	-1	-1
929	930	0,0247867	0,0003527	0,0003527	0	-1	-1
930	931	0,0028575	0,0002799	0,0002799	3,92874E+12	2,22629E+13	3,06461E-15
930	932	0,0090091	0,0002799	0,0002799	3,91503E+12	2,21851E+13	3,07535E-15
865	933	0,0282592	0,0003527	0,0003527	0	-1	-1
933	934	0,0100707	0,0003527	0,0003527	0	-1	-1
934	935	0,0086105	0,0003527	0,0003527	0	-1	-1
935	936	0,0205163	0,0003527	0,0003527	1,95214E+12	1,10621E+13	6,16763E-15
865	937	0,0299888	0,0003527	0,0003527	0	-1	-1
937	938	0,0140632	0,0002799	0,0002799	0	-1	-1
938	939	0,0248241	0,0002799	0,0002799	0	-1	-1
939	940	0,0312889	0,0002799	0,0002799	0	-1	-1
940	941	0,0089092	0,0002222	0,0002222	7,57814E+12	4,29428E+13	1,58879E-15
940	942	0,0266214	0,0002222	0,0002222	0	-1	-1
942	943	0,0161108	0,0001540	0,0001540	0	-1	-1
943	944	0,0241430	0,0001540	0,0001540	2,14571E+13	1,2159E+14	5,61123E-16
942	945	0,0192371	0,0001540	0,0001540	2,19681E+13	1,24486E+14	5,48071E-16
942	946	0,0269556	0,0001540	0,0001540	0	-1	-1
946	947	0,0259027	0,0001223	0,0001223	4,19739E+13	2,37852E+14	2,86847E-16
946	948	0,0307884	0,0001223	0,0001223	4,16746E+13	2,36156E+14	2,88907E-16
937	949	0,0214708	0,0002799	0,0002799	0	-1	-1
949	950	0,0059492	0,0002222	0,0002222	7,812E+12	4,4268E+13	1,54123E-15
949	951	0,0158427	0,0002222	0,0002222	0	-1	-1
951	952	0,0152838	0,0002222	0,0002222	0	-1	-1
952	953	0,0189380	0,0002222	0,0002222	7,5641E+12	4,28632E+13	1,59174E-15

704	954	0,0261308	0,0017773	0,0017773	0	-1	-1
954	955	0,0046654	0,0014106	0,0014106	0	-1	-1
955	956	0,0180347	0,0014106	0,0014106	32146170315	1,82162E+11	3,74541E-13
954	957	0,0127707	0,0014106	0,0014106	32180496331	1,82356E+11	3,74142E-13
696	958	0,0090538	0,0028212	0,0028212	4051978930	22961213936	2,9714E-12
695	959	0,0299636	0,0035545	0,0035545	0	-1	-1
959	960	0,0118171	0,0024646	0,0024646	0	-1	-1
960	961	0,0161407	0,0024646	0,0024646	6063804327	34361557851	1,98556E-12
959	962	0,0159967	0,0024646	0,0024646	0	-1	-1
962	963	0,0178066	0,0024646	0,0024646	0	-1	-1
963	964	0,0135445	0,0019561	0,0019561	12110609207	68626785508	9,94175E-13
963	965	0,0356962	0,0019561	0,0019561	0	-1	-1
965	966	0,0141115	0,0013563	0,0013563	0	-1	-1
966	967	0,0189489	0,0013563	0,0013563	0	-1	-1
967	968	0,0180874	0,0013563	0,0013563	0	-1	-1
968	969	0,0089680	0,0007932	0,0007932	1,80004E+11	1,02002E+12	6,68878E-14
968	970	0,0210132	0,0007932	0,0007932	0	-1	-1
970	971	0,0245813	0,0007932	0,0007932	0	-1	-1
971	972	0,0198964	0,0007932	0,0007932	0	-1	-1
972	973	0,0183310	0,0007932	0,0007932	0	-1	-1
973	974	0,0455495	0,0007932	0,0007932	0	-1	-1
974	975	0,0069941	0,0006295	0,0006295	3,5107E+11	1,9894E+12	3,42953E-14
974	976	0,0101109	0,0006295	0,0006295	3,50799E+11	1,98786E+12	3,43219E-14
968	977	0,0296035	0,0007932	0,0007932	0	-1	-1
977	978	0,0118426	0,0006295	0,0006295	0	-1	-1
978	979	0,0140080	0,0004997	0,0004997	7,12021E+11	4,03479E+12	1,69097E-14
978	980	0,0157744	0,0004997	0,0004997	0	-1	-1
980	981	0,0060139	0,0004997	0,0004997	0	-1	-1
981	982	0,0200876	0,0004997	0,0004997	0	-1	-1
982	983	0,0152725	0,0004997	0,0004997	0	-1	-1
983	984	0,0087700	0,0004997	0,0004997	7,00622E+11	3,97019E+12	1,71848E-14
977	985	0,0119521	0,0006295	0,0006295	3,57539E+11	2,02605E+12	3,36748E-14
968	986	0,0362799	0,0007932	0,0007932	0	-1	-1
986	987	0,0055743	0,0004997	0,0004997	0	-1	-1
987	988	0,0246890	0,0004997	0,0004997	0	-1	-1
988	989	0,0210390	0,0004997	0,0004997	0	-1	-1
989	990	0,0328646	0,0004997	0,0004997	6,97755E+11	3,95395E+12	1,72554E-14
986	991	0,0115133	0,0004997	0,0004997	0	-1	-1
991	992	0,0115265	0,0004997	0,0004997	7,11178E+11	4,03001E+12	1,69297E-14
986	993	0,0141585	0,0004997	0,0004997	7,13129E+11	4,04106E+12	1,68834E-14
986	994	0,0205950	0,0004997	0,0004997	0	-1	-1
994	995	0,0119412	0,0003966	0,0003966	1,41682E+12	8,02866E+12	8,49793E-15
994	996	0,0221478	0,0003966	0,0003966	0	-1	-1
996	997	0,0150092	0,0002498	0,0002498	0	-1	-1
997	998	0,0148541	0,0001983	0,0001983	1,10524E+13	6,26304E+13	1,08936E-15
997	999	0,0300534	0,0001983	0,0001983	0	-1	-1
999	1000	0,0182929	0,0001574	0,0001574	2,14276E+13	1,21423E+14	5,61895E-16
999	1001	0,0185791	0,0001574	0,0001574	0	-1	-1
1001	1002	0,0234773	0,0001574	0,0001574	0	-1	-1
1002	1003	0,0244278	0,0001574	0,0001574	0	-1	-1
1003	1004	0,0193498	0,0001574	0,0001574	0	-1	-1
1004	1005	0,0078068	0,0001574	0,0001574	0	-1	-1
1005	1006	0,0161714	0,0001574	0,0001574	1,93859E+13	1,09854E+14	6,21073E-16

996	1007	0,0208144	0,0002498	0,0002498	0	-1	-1
1007	1008	0,0097986	0,0001732	0,0001732	0	-1	-1
1008	1009	0,0125484	0,0001732	0,0001732	1,6375E+13	9,27917E+13	7,35271E-16
1007	1010	0,0291289	0,0001732	0,0001732	0	-1	-1
1010	1011	0,0056259	0,0001375	0,0001375	0	-1	-1
1011	1012	0,0111640	0,0000953	0,0000953	9,51346E+13	5,39096E+14	1,26558E-16
1011	1013	0,0119424	0,0000953	0,0000953	0	-1	-1
1013	1014	0,0283884	0,0000953	0,0000953	0	-1	-1
1014	1015	0,0065412	0,0000757	0,0000757	1,77869E+14	1,00792E+15	6,76906E-17
1014	1016	0,0154777	0,0000757	0,0000757	1,74137E+14	9,86775E+14	6,91414E-17
1011	1017	0,0347525	0,0000953	0,0000953	9,1225E+13	5,16942E+14	1,31982E-16
1010	1018	0,0387934	0,0001375	0,0001375	0	-1	-1
1018	1019	0,0187326	0,0001375	0,0001375	0	-1	-1
1019	1020	0,0234848	0,0001375	0,0001375	0	-1	-1
1020	1021	0,0091556	0,0000953	0,0000953	0	-1	-1
1021	1022	0,0116435	0,0000953	0,0000953	8,48745E+13	4,80956E+14	1,41857E-16
1020	1023	0,0256153	0,0000953	0,0000953	8,40763E+13	4,76432E+14	1,43204E-16
1020	1024	0,0269710	0,0000953	0,0000953	8,38516E+13	4,75159E+14	1,43588E-16
1007	1025	0,0309608	0,0001732	0,0001732	0	-1	-1
1025	1026	0,0206758	0,0001732	0,0001732	0	-1	-1
1026	1027	0,0087679	0,0001732	0,0001732	1,57965E+13	8,95133E+13	7,622E-16
996	1028	0,0220520	0,0002498	0,0002498	0	-1	-1
1028	1029	0,0145954	0,0001983	0,0001983	0	-1	-1
1029	1030	0,0178743	0,0001983	0,0001983	0	-1	-1
1030	1031	0,0137299	0,0001983	0,0001983	0	-1	-1
1031	1032	0,0087340	0,0001983	0,0001983	1,06481E+13	6,03392E+13	1,13072E-15
1028	1033	0,0217563	0,0001983	0,0001983	0	-1	-1
1033	1034	0,0390516	0,0001983	0,0001983	0	-1	-1
1034	1035	0,0086236	0,0001160	0,0001160	5,23277E+13	2,96524E+14	2,3009E-16
1034	1036	0,0178561	0,0001160	0,0001160	5,16289E+13	2,92564E+14	2,33204E-16
1034	1037	0,0206784	0,0001160	0,0001160	5,14152E+13	2,91353E+14	2,34173E-16
1034	1038	0,0213587	0,0001160	0,0001160	0	-1	-1
1038	1039	0,0217378	0,0000920	0,0000920	9,85812E+13	5,58627E+14	1,22133E-16
1038	1040	0,0304896	0,0000920	0,0000920	0	-1	-1
1040	1041	0,0262191	0,0000920	0,0000920	0	-1	-1
1041	1042	0,0124858	0,0000920	0,0000920	8,95294E+13	5,07333E+14	1,34482E-16
1034	1043	0,0283248	0,0001160	0,0001160	0	-1	-1
1043	1044	0,0256456	0,0001160	0,0001160	0	-1	-1
1044	1045	0,0145830	0,0001160	0,0001160	4,77913E+13	2,70818E+14	2,5193E-16
996	1046	0,0231468	0,0002498	0,0002498	0	-1	-1
1046	1047	0,0087288	0,0002498	0,0002498	5,53271E+12	3,1352E+13	2,17616E-15
968	1048	0,0365682	0,0007932	0,0007932	0	-1	-1
1048	1049	0,0173574	0,0007932	0,0007932	1,78449E+11	1,01121E+12	6,74706E-14
965	1050	0,0166813	0,0013563	0,0013563	36202227965	2,05146E+11	3,32578E-13
965	1051	0,0175425	0,0013563	0,0013563	0	-1	-1
1051	1052	0,0065170	0,0013563	0,0013563	0	-1	-1
1052	1053	0,0293945	0,0013563	0,0013563	0	-1	-1
1053	1054	0,0138812	0,0013563	0,0013563	35997338863	2,03985E+11	3,34471E-13
959	1055	0,0332430	0,0024646	0,0024646	0	-1	-1
1055	1056	0,0071159	0,0019561	0,0019561	0	-1	-1
1056	1057	0,0153946	0,0019561	0,0019561	12102643036	68581643872	9,9483E-13
1055	1058	0,0188263	0,0019561	0,0019561	0	-1	-1
1058	1059	0,0153350	0,0019561	0,0019561	12091751240	68519923694	9,95726E-13

390	1060	0,0635925	0,0044784	0,0044784	0	-1	-1
1060	1061	0,0060377	0,0031052	0,0031052	0	-1	-1
1061	1062	0,0114930	0,0024646	0,0024646	0	-1	-1
1062	1063	0,0125789	0,0017088	0,0017088	0	-1	-1
1063	1064	0,0129425	0,0017088	0,0017088	0	-1	-1
1064	1065	0,0237016	0,0013563	0,0013563	36237367752	2,05345E+11	3,32256E-13
1064	1066	0,0331050	0,0013563	0,0013563	0	-1	-1
1066	1067	0,0090541	0,0010765	0,0010765	72306380920	4,09736E+11	1,66515E-13
1066	1068	0,0313547	0,0010765	0,0010765	0	-1	-1
1068	1069	0,0092316	0,0008544	0,0008544	1,43921E+11	8,15553E+11	8,36574E-14
1068	1070	0,0133162	0,0008544	0,0008544	1,43816E+11	8,14958E+11	8,37185E-14
1062	1071	0,0183823	0,0017088	0,0017088	0	-1	-1
1071	1072	0,0303329	0,0017088	0,0017088	0	-1	-1
1072	1073	0,0109211	0,0011848	0,0011848	0	-1	-1
1073	1074	0,0145545	0,0009404	0,0009404	0	-1	-1
1074	1075	0,0057315	0,0009404	0,0009404	0	-1	-1
1075	1076	0,0177380	0,0009404	0,0009404	0	-1	-1
1076	1077	0,0102744	0,0009404	0,0009404	1,07779E+11	6,1075E+11	1,1171E-13
1073	1078	0,0164965	0,0009404	0,0009404	0	-1	-1
1078	1079	0,0294491	0,0006520	0,0006520	0	-1	-1
1079	1080	0,0295827	0,0004521	0,0004521	9,58641E+11	5,4323E+12	1,25595E-14
1079	1081	0,0300625	0,0004521	0,0004521	9,58484E+11	5,43141E+12	1,25616E-14
1079	1082	0,0316778	0,0004521	0,0004521	0	-1	-1
1082	1083	0,0152876	0,0002848	0,0002848	3,80001E+12	2,15334E+13	3,16843E-15
1082	1084	0,0174100	0,0002848	0,0002848	3,7956E+12	2,15084E+13	3,17211E-15
1082	1085	0,0240211	0,0002848	0,0002848	0	-1	-1
1085	1086	0,0254615	0,0002848	0,0002848	0	-1	-1
1086	1087	0,0137757	0,0001567	0,0001567	2,20608E+13	1,25011E+14	5,45767E-16
1086	1088	0,0169958	0,0001567	0,0001567	0	-1	-1
1088	1089	0,0340110	0,0001567	0,0001567	0	-1	-1
1089	1090	0,0211029	0,0001567	0,0001567	0	-1	-1
1090	1091	0,0276414	0,0001244	0,0001244	0	-1	-1
1091	1092	0,0160595	0,0001244	0,0001244	3,89777E+13	2,20874E+14	3,08896E-16
1090	1093	0,0306297	0,0001244	0,0001244	0	-1	-1
1093	1094	0,0171840	0,0000987	0,0000987	7,69748E+13	4,36191E+14	1,56416E-16
1093	1095	0,0327990	0,0000987	0,0000987	7,47259E+13	4,23447E+14	1,61123E-16
1086	1096	0,0231947	0,0001567	0,0001567	0	-1	-1
1096	1097	0,0240516	0,0001567	0,0001567	0	-1	-1
1097	1098	0,0132078	0,0000987	0,0000987	8,33044E+13	4,72058E+14	1,44531E-16
1097	1099	0,0143595	0,0000987	0,0000987	8,31385E+13	4,71118E+14	1,44819E-16
1097	1100	0,0145529	0,0000987	0,0000987	0	-1	-1
1100	1101	0,0061127	0,0000784	0,0000784	0	-1	-1
1101	1102	0,0286966	0,0000622	0,0000622	0	-1	-1
1102	1103	0,0194149	0,0000622	0,0000622	2,8401E+14	1,60939E+15	4,23931E-17
1101	1104	0,0355878	0,0000622	0,0000622	2,95462E+14	1,67429E+15	4,07499E-17
1100	1105	0,0069864	0,0000784	0,0000784	0	-1	-1
1105	1106	0,0091276	0,0000784	0,0000784	1,60374E+14	9,08783E+14	7,50752E-17
1097	1107	0,0174191	0,0000987	0,0000987	8,26979E+13	4,68621E+14	1,45591E-16
1086	1108	0,0239416	0,0001567	0,0001567	2,18302E+13	1,23705E+14	5,51532E-16
1086	1109	0,0270927	0,0001567	0,0001567	2,17588E+13	1,233E+14	5,53344E-16
1086	1110	0,0280428	0,0001567	0,0001567	0	-1	-1
1110	1111	0,0151945	0,0001244	0,0001244	0	-1	-1
1111	1112	0,0214534	0,0001244	0,0001244	0	-1	-1

1112	1113	0,0289008	0,0001244	0,0001244	0	-1	-1
1113	1114	0,0170702	0,0001244	0,0001244	0	-1	-1
1114	1115	0,0186232	0,0001244	0,0001244	0	-1	-1
1115	1116	0,0096947	0,0001244	0,0001244	0	-1	-1
1116	1117	0,0143076	0,0001244	0,0001244	3,63162E+13	2,05792E+14	3,31534E-16
1110	1118	0,0199104	0,0001244	0,0001244	0	-1	-1
1118	1119	0,0117897	0,0001244	0,0001244	0	-1	-1
1119	1120	0,0227420	0,0000987	0,0000987	0	-1	-1
1120	1121	0,0147409	0,0000987	0,0000987	7,7927E+13	4,41586E+14	1,54504E-16
1119	1122	0,0264908	0,0000987	0,0000987	0	-1	-1
1122	1123	0,0145834	0,0000987	0,0000987	7,74098E+13	4,38656E+14	1,55537E-16
1082	1124	0,0330301	0,0002848	0,0002848	0	-1	-1
1124	1125	0,0062904	0,0002261	0,0002261	0	-1	-1
1125	1126	0,0172566	0,0001567	0,0001567	2,20883E+13	1,25167E+14	5,45088E-16
1125	1127	0,0197903	0,0001567	0,0001567	2,20308E+13	1,24841E+14	5,4651E-16
1125	1128	0,0299671	0,0001567	0,0001567	0	-1	-1
1128	1129	0,0366043	0,0001244	0,0001244	0	-1	-1
1129	1130	0,0092606	0,0000863	0,0000863	0	-1	-1
1130	1131	0,0322938	0,0000863	0,0000863	0	-1	-1
1131	1132	0,0142661	0,0000863	0,0000863	1,1072E+14	6,27413E+14	1,08743E-16
1129	1133	0,0099900	0,0000863	0,0000863	1,22053E+14	6,91636E+14	9,86459E-17
1129	1134	0,0196694	0,0000863	0,0000863	1,1966E+14	6,78072E+14	1,00619E-16
1128	1135	0,0415271	0,0001244	0,0001244	0	-1	-1
1135	1136	0,0229031	0,0001244	0,0001244	0	-1	-1
1136	1137	0,0072066	0,0001244	0,0001244	0	-1	-1
1137	1138	0,0128342	0,0000987	0,0000987	7,7163E+13	4,37257E+14	1,56034E-16
1137	1139	0,0362833	0,0000987	0,0000987	0	-1	-1
1139	1140	0,0108358	0,0000784	0,0000784	1,43639E+14	8,13957E+14	8,38215E-17
1139	1141	0,0212076	0,0000784	0,0000784	0	-1	-1
1141	1142	0,0188144	0,0000622	0,0000622	2,62546E+14	1,48776E+15	4,58589E-17
1141	1143	0,0415926	0,0000622	0,0000622	0	-1	-1
1143	1144	0,0248842	0,0000494	0,0000494	0	-1	-1
1144	1145	0,0122186	0,0000494	0,0000494	0	-1	-1
1145	1146	0,0172857	0,0000494	0,0000494	3,58105E+14	2,02926E+15	3,36216E-17
1143	1147	0,0361463	0,0000494	0,0000494	4,0014E+14	2,26746E+15	3,00896E-17
1124	1148	0,0245056	0,0002261	0,0002261	0	-1	-1
1148	1149	0,0120524	0,0001794	0,0001794	1,46363E+13	8,2939E+13	8,22618E-16
1148	1150	0,0241886	0,0001794	0,0001794	0	-1	-1
1150	1151	0,0130702	0,0001424	0,0001424	2,85169E+13	1,61596E+14	4,22208E-16
1150	1152	0,0283305	0,0001424	0,0001424	0	-1	-1
1152	1153	0,0307177	0,0001424	0,0001424	0	-1	-1
1153	1154	0,0236216	0,0000987	0,0000987	0	-1	-1
1154	1155	0,0139048	0,0000987	0,0000987	7,5555E+13	4,28145E+14	1,59355E-16
1153	1156	0,0289516	0,0000987	0,0000987	7,67899E+13	4,35143E+14	1,56792E-16
1153	1157	0,0311432	0,0000987	0,0000987	0	-1	-1
1157	1158	0,0120199	0,0000987	0,0000987	7,47432E+13	4,23545E+14	1,61086E-16
1078	1159	0,0320306	0,0006520	0,0006520	0	-1	-1
1159	1160	0,0171497	0,0006520	0,0006520	0	-1	-1
1160	1161	0,0232431	0,0006520	0,0006520	0	-1	-1
1161	1162	0,0128927	0,0006520	0,0006520	3,18547E+11	1,8051E+12	3,77968E-14
1078	1163	0,0326343	0,0006520	0,0006520	0	-1	-1
1163	1164	0,0147619	0,0004521	0,0004521	9,62773E+11	5,45572E+12	1,25056E-14
1163	1165	0,0152001	0,0004521	0,0004521	0	-1	-1

1165	1166	0,0128806	0,0004521	0,0004521	9,5841E+11	5,43099E+12	1,25626E-14
1163	1167	0,0261204	0,0004521	0,0004521	0	-1	-1
1167	1168	0,0090846	0,0004521	0,0004521	0	-1	-1
1168	1169	0,0279663	0,0004521	0,0004521	0	-1	-1
1169	1170	0,0118449	0,0002848	0,0002848	0	-1	-1
1170	1171	0,0327498	0,0002848	0,0002848	0	-1	-1
1171	1172	0,0222159	0,0002261	0,0002261	0	-1	-1
1172	1173	0,0166185	0,0001567	0,0001567	2,1443E+13	1,2151E+14	5,61493E-16
1172	1174	0,0173430	0,0001567	0,0001567	2,14265E+13	1,21417E+14	5,61924E-16
1172	1175	0,0314436	0,0001567	0,0001567	2,11067E+13	1,19605E+14	5,70438E-16
1171	1176	0,0417954	0,0002261	0,0002261	0	-1	-1
1176	1177	0,0165843	0,0001794	0,0001794	1,41222E+13	8,00261E+13	8,5256E-16
1176	1178	0,0398165	0,0001794	0,0001794	1,38154E+13	7,8287E+13	8,71499E-16
1169	1179	0,0151112	0,0002848	0,0002848	0	-1	-1
1179	1180	0,0046703	0,0002261	0,0002261	7,48795E+12	4,24317E+13	1,60793E-15
1179	1181	0,0228546	0,0002261	0,0002261	0	-1	-1
1181	1182	0,0222279	0,0001794	0,0001794	1,44916E+13	8,21192E+13	8,30829E-16
1181	1183	0,0346703	0,0001794	0,0001794	0	-1	-1
1183	1184	0,0186974	0,0001244	0,0001244	0	-1	-1
1184	1185	0,0121812	0,0001244	0,0001244	4,1217E+13	2,33563E+14	2,92114E-16
1183	1186	0,0249293	0,0001244	0,0001244	0	-1	-1
1186	1187	0,0152308	0,0000987	0,0000987	8,09205E+13	4,58549E+14	1,48789E-16
1186	1188	0,0266471	0,0000987	0,0000987	0	-1	-1
1188	1189	0,0152849	0,0000987	0,0000987	0	-1	-1
1189	1190	0,0052636	0,0000987	0,0000987	0	-1	-1
1190	1191	0,0197099	0,0000987	0,0000987	0	-1	-1
1191	1192	0,0111701	0,0000987	0,0000987	7,18696E+13	4,07261E+14	1,67527E-16
1183	1193	0,0293664	0,0001244	0,0001244	0	-1	-1
1193	1194	0,0237238	0,0001244	0,0001244	3,99475E+13	2,26369E+14	3,01397E-16
1169	1195	0,0157415	0,0002848	0,0002848	3,7549E+12	2,12778E+13	3,20649E-15
1169	1196	0,0260700	0,0002848	0,0002848	0	-1	-1
1196	1197	0,0146159	0,0002848	0,0002848	0	-1	-1
1197	1198	0,0084019	0,0002848	0,0002848	0	-1	-1
1198	1199	0,0232182	0,0002848	0,0002848	3,63723E+12	2,0611E+13	3,31023E-15
1072	1200	0,0204529	0,0011848	0,0011848	0	-1	-1
1200	1201	0,0125819	0,0009404	0,0009404	1,08272E+11	6,13541E+11	1,11202E-13
1200	1202	0,0167899	0,0009404	0,0009404	0	-1	-1
1202	1203	0,0068350	0,0006520	0,0006520	0	-1	-1
1203	1204	0,0041437	0,0005175	0,0005175	6,47364E+11	3,6684E+12	1,85986E-14
1203	1205	0,0132617	0,0005175	0,0005175	0	-1	-1
1205	1206	0,0286312	0,0005175	0,0005175	0	-1	-1
1206	1207	0,0179244	0,0004108	0,0004108	0	-1	-1
1207	1208	0,0130514	0,0003260	0,0003260	2,5276E+12	1,4323E+13	4,76344E-15
1207	1209	0,0233828	0,0003260	0,0003260	0	-1	-1
1209	1210	0,0122172	0,0002261	0,0002261	7,48119E+12	4,23934E+13	1,60938E-15
1209	1211	0,0228259	0,0002261	0,0002261	7,42558E+12	4,20783E+13	1,62143E-15
1209	1212	0,0346673	0,0002261	0,0002261	0	-1	-1
1212	1213	0,0245277	0,0001794	0,0001794	0	-1	-1
1213	1214	0,0258709	0,0001794	0,0001794	1,40613E+13	7,96806E+13	8,56257E-16
1212	1215	0,0305830	0,0001794	0,0001794	0	-1	-1
1215	1216	0,0189974	0,0001424	0,0001424	2,80137E+13	1,58744E+14	4,29792E-16
1215	1217	0,0224224	0,0001424	0,0001424	0	-1	-1
1217	1218	0,0152704	0,0001130	0,0001130	5,45186E+13	3,08939E+14	2,20843E-16

1217	1219	0,0263672	0,0001130	0,0001130	5,35879E+13	3,03665E+14	2,24679E-16
1206	1220	0,0194143	0,0004108	0,0004108	0	-1	-1
1220	1221	0,0216550	0,0004108	0,0004108	0	-1	-1
1221	1222	0,0244067	0,0004108	0,0004108	0	-1	-1
1222	1223	0,0208818	0,0004108	0,0004108	1,2388E+12	7,01987E+12	9,71913E-15
1202	1224	0,0088249	0,0006520	0,0006520	3,23927E+11	1,83559E+12	3,71691E-14
1202	1225	0,0176501	0,0006520	0,0006520	0	-1	-1
1225	1226	0,0125151	0,0005175	0,0005175	0	-1	-1
1226	1227	0,0089450	0,0005175	0,0005175	6,42422E+11	3,64039E+12	1,87417E-14
1225	1228	0,0141415	0,0005175	0,0005175	0	-1	-1
1228	1229	0,0125079	0,0005175	0,0005175	6,41432E+11	3,63478E+12	1,87706E-14
1072	1230	0,0247942	0,0011848	0,0011848	0	-1	-1
1230	1231	0,0042395	0,0008215	0,0008215	0	-1	-1
1231	1232	0,0096838	0,0008215	0,0008215	0	-1	-1
1232	1233	0,0246492	0,0008215	0,0008215	1,61489E+11	9,15103E+11	7,45567E-14
1230	1234	0,0153233	0,0008215	0,0008215	0	-1	-1
1234	1235	0,0209779	0,0008215	0,0008215	0	-1	-1
1235	1236	0,0128148	0,0006520	0,0006520	0	-1	-1
1236	1237	0,0162498	0,0006520	0,0006520	3,20913E+11	1,81851E+12	3,75182E-14
1235	1238	0,0242714	0,0006520	0,0006520	0	-1	-1
1238	1239	0,0141808	0,0005175	0,0005175	6,39846E+11	3,6258E+12	1,88171E-14
1238	1240	0,0209734	0,0005175	0,0005175	6,3855E+11	3,61845E+12	1,88553E-14
1230	1241	0,0195671	0,0008215	0,0008215	0	-1	-1
1241	1242	0,0120660	0,0008215	0,0008215	1,61697E+11	9,16285E+11	7,44605E-14
1062	1243	0,0201833	0,0017088	0,0017088	18175187771	1,02993E+11	6,62445E-13
1061	1244	0,0254011	0,0024646	0,0024646	0	-1	-1
1244	1245	0,0124918	0,0017088	0,0017088	0	-1	-1
1245	1246	0,0168729	0,0017088	0,0017088	18144970032	1,02821E+11	6,63549E-13
1244	1247	0,0144471	0,0017088	0,0017088	0	-1	-1
1247	1248	0,0231598	0,0013563	0,0013563	0	-1	-1
1248	1249	0,0197005	0,0008544	0,0008544	1,44471E+11	8,18667E+11	8,33392E-14
1248	1250	0,0244584	0,0008544	0,0008544	0	-1	-1
1250	1251	0,0122898	0,0008544	0,0008544	0	-1	-1
1251	1252	0,0075008	0,0008544	0,0008544	1,4384E+11	8,15094E+11	8,37045E-14
1248	1253	0,0249596	0,0008544	0,0008544	0	-1	-1
1253	1254	0,0313650	0,0008544	0,0008544	0	-1	-1
1254	1255	0,0063199	0,0006782	0,0006782	0	-1	-1
1255	1256	0,0131352	0,0004702	0,0004702	8,56275E+11	4,85222E+12	1,4061E-14
1255	1257	0,0138691	0,0004702	0,0004702	8,56069E+11	4,85106E+12	1,40644E-14
1255	1258	0,0143693	0,0004702	0,0004702	8,55929E+11	4,85027E+12	1,40667E-14
1254	1259	0,0204090	0,0006782	0,0006782	0	-1	-1
1259	1260	0,0288827	0,0006782	0,0006782	0	-1	-1
1260	1261	0,0289586	0,0006782	0,0006782	0	-1	-1
1261	1262	0,0252233	0,0006782	0,0006782	2,80363E+11	1,58873E+12	4,29445E-14
1248	1263	0,0269079	0,0008544	0,0008544	0	-1	-1
1263	1264	0,0224124	0,0008544	0,0008544	0	-1	-1
1264	1265	0,0121725	0,0008544	0,0008544	0	-1	-1
1265	1266	0,0126433	0,0005924	0,0005924	4,28787E+11	2,42979E+12	2,80794E-14
1265	1267	0,0142535	0,0005924	0,0005924	0	-1	-1
1267	1268	0,0078165	0,0004702	0,0004702	8,55027E+11	4,84515E+12	1,40815E-14
1267	1269	0,0253554	0,0004702	0,0004702	8,50116E+11	4,81732E+12	1,41629E-14
1265	1270	0,0260998	0,0005924	0,0005924	0	-1	-1
1270	1271	0,0176620	0,0004702	0,0004702	0	-1	-1

1271	1272	0,0256106	0,0004702	0,0004702	8,42466E+11	4,77397E+12	1,42915E-14
1270	1273	0,0212114	0,0004702	0,0004702	8,48643E+11	4,80898E+12	1,41874E-14
1247	1274	0,0282073	0,0013563	0,0013563	0	-1	-1
1274	1275	0,0118242	0,0009404	0,0009404	0	-1	-1
1275	1276	0,0155285	0,0007464	0,0007464	2,16244E+11	1,22538E+12	5,56782E-14
1275	1277	0,0484870	0,0007464	0,0007464	0	-1	-1
1277	1278	0,0159905	0,0005175	0,0005175	0	-1	-1
1278	1279	0,0135831	0,0003027	0,0003027	0	-1	-1
1279	1280	0,0183175	0,0002402	0,0002402	0	-1	-1
1280	1281	0,0165204	0,0001907	0,0001907	0	-1	-1
1281	1282	0,0297659	0,0001513	0,0001513	2,40551E+13	1,36312E+14	5,00521E-16
1281	1283	0,0374312	0,0001513	0,0001513	2,3855E+13	1,35178E+14	5,04719E-16
1280	1284	0,0298162	0,0001907	0,0001907	0	-1	-1
1284	1285	0,0076648	0,0001907	0,0001907	0	-1	-1
1285	1286	0,0004501	0,0001907	0,0001907	1,21942E+13	6,91006E+13	9,87358E-16
1279	1287	0,0282621	0,0002402	0,0002402	0	-1	-1
1287	1288	0,0214115	0,0002402	0,0002402	0	-1	-1
1288	1289	0,0254981	0,0002402	0,0002402	6,05985E+12	3,43392E+13	1,98686E-15
1278	1290	0,0213805	0,0003027	0,0003027	0	-1	-1
1290	1291	0,0308843	0,0003027	0,0003027	0	-1	-1
1291	1292	0,0121952	0,0001907	0,0001907	0	-1	-1
1292	1293	0,0116109	0,0001513	0,0001513	0	-1	-1
1293	1294	0,0248669	0,0001513	0,0001513	2,37659E+13	1,34673E+14	5,06612E-16
1292	1295	0,0265525	0,0001513	0,0001513	0	-1	-1
1295	1296	0,0195759	0,0001513	0,0001513	2,35139E+13	1,33246E+14	5,12039E-16
1291	1297	0,0161955	0,0001907	0,0001907	0	-1	-1
1297	1298	0,0128931	0,0001907	0,0001907	0	-1	-1
1298	1299	0,0117793	0,0001907	0,0001907	1,2062E+13	6,83513E+13	9,98182E-16
1291	1300	0,0163523	0,0001907	0,0001907	1,23159E+13	6,97903E+13	9,776E-16
1291	1301	0,0215786	0,0001907	0,0001907	1,22618E+13	6,94836E+13	9,81917E-16
1278	1302	0,0226224	0,0003027	0,0003027	3,16969E+12	1,79616E+13	3,7985E-15
1278	1303	0,0240496	0,0003027	0,0003027	0	-1	-1
1303	1304	0,0139551	0,0003027	0,0003027	3,1446E+12	1,78194E+13	3,82881E-15
1278	1305	0,0301165	0,0003027	0,0003027	0	-1	-1
1305	1306	0,0112066	0,0003027	0,0003027	0	-1	-1
1306	1307	0,0129640	0,0002402	0,0002402	6,22507E+12	3,52754E+13	1,93412E-15
1306	1308	0,0152467	0,0002402	0,0002402	0	-1	-1
1308	1309	0,0110315	0,0002402	0,0002402	6,17034E+12	3,49653E+13	1,95128E-15
1277	1310	0,0261411	0,0005175	0,0005175	6,39383E+11	3,62317E+12	1,88308E-14
1277	1311	0,0262619	0,0005175	0,0005175	0	-1	-1
1311	1312	0,0175376	0,0005175	0,0005175	0	-1	-1
1312	1313	0,0123190	0,0004108	0,0004108	1,2661E+12	7,17459E+12	9,50954E-15
1312	1314	0,0211492	0,0004108	0,0004108	0	-1	-1
1314	1315	0,0210485	0,0004108	0,0004108	0	-1	-1
1315	1316	0,0165235	0,0004108	0,0004108	1,24379E+12	7,04816E+12	9,68012E-15
1274	1317	0,0124378	0,0009404	0,0009404	0	-1	-1
1317	1318	0,0134683	0,0007464	0,0007464	0	-1	-1
1318	1319	0,0138226	0,0005924	0,0005924	4,3109E+11	2,44284E+12	2,79293E-14
1318	1320	0,0336864	0,0005924	0,0005924	0	-1	-1
1320	1321	0,0159223	0,0004108	0,0004108	1,27899E+12	7,24763E+12	9,41371E-15
1320	1322	0,0203671	0,0004108	0,0004108	0	-1	-1
1322	1323	0,0170389	0,0003260	0,0003260	2,53307E+12	1,4354E+13	4,75316E-15
1322	1324	0,0176766	0,0003260	0,0003260	0	-1	-1

1324	1325	0,0105475	0,0003260	0,0003260	2,51952E+12	1,42773E+13	4,77872E-15
1320	1326	0,0362533	0,0004108	0,0004108	0	-1	-1
1326	1327	0,0391268	0,0004108	0,0004108	0	-1	-1
1327	1328	0,0103466	0,0002588	0,0002588	0	-1	-1
1328	1329	0,0231901	0,0001794	0,0001794	1,46038E+13	8,27547E+13	8,2445E-16
1328	1330	0,0232236	0,0001794	0,0001794	0	-1	-1
1330	1331	0,0191222	0,0001794	0,0001794	1,43507E+13	8,13208E+13	8,38986E-16
1328	1332	0,0299740	0,0001794	0,0001794	0	-1	-1
1332	1333	0,0183836	0,0001424	0,0001424	0	-1	-1
1333	1334	0,0277374	0,0001424	0,0001424	0	-1	-1
1334	1335	0,0119162	0,0001424	0,0001424	2,70965E+13	1,53547E+14	4,4434E-16
1332	1336	0,0372752	0,0001424	0,0001424	0	-1	-1
1336	1337	0,0072704	0,0001130	0,0001130	0	-1	-1
1337	1338	0,0145730	0,0001130	0,0001130	5,37431E+13	3,04544E+14	2,2403E-16
1336	1339	0,0247287	0,0001130	0,0001130	0	-1	-1
1339	1340	0,0153388	0,0000897	0,0000897	0	-1	-1
1340	1341	0,0043860	0,0000897	0,0000897	1,02833E+14	5,82722E+14	1,17083E-16
1339	1342	0,0173544	0,0000897	0,0000897	0	-1	-1
1342	1343	0,0125564	0,0000712	0,0000712	1,99981E+14	1,13323E+15	6,0206E-17
1342	1344	0,0128577	0,0000712	0,0000712	1,99821E+14	1,13232E+15	6,02543E-17
1327	1345	0,0152397	0,0002588	0,0002588	0	-1	-1
1345	1346	0,0220118	0,0002588	0,0002588	0	-1	-1
1346	1347	0,0156594	0,0002588	0,0002588	0	-1	-1
1347	1348	0,0104382	0,0002054	0,0002054	0	-1	-1
1348	1349	0,0200087	0,0002054	0,0002054	9,44594E+12	5,3527E+13	1,27463E-15
1347	1350	0,0168686	0,0002054	0,0002054	9,5504E+12	5,41189E+13	1,26069E-15
1327	1351	0,0192875	0,0002588	0,0002588	4,94273E+12	2,80088E+13	2,43591E-15
1327	1352	0,0218019	0,0002588	0,0002588	4,93506E+12	2,79653E+13	2,4397E-15
1317	1353	0,0279577	0,0007464	0,0007464	0	-1	-1
1353	1354	0,0272983	0,0007464	0,0007464	0	-1	-1
1354	1355	0,0256623	0,0005924	0,0005924	0	-1	-1
1355	1356	0,0095837	0,0004702	0,0004702	0	-1	-1
1356	1357	0,0089522	0,0003732	0,0003732	1,69267E+12	9,5918E+12	7,11306E-15
1356	1358	0,0317940	0,0003732	0,0003732	0	-1	-1
1358	1359	0,0271982	0,0003732	0,0003732	0	-1	-1
1359	1360	0,0194204	0,0003732	0,0003732	1,64366E+12	9,31407E+12	7,32516E-15
1355	1361	0,0191469	0,0004702	0,0004702	8,46816E+11	4,79862E+12	1,4218E-14
1354	1362	0,0303870	0,0005924	0,0005924	0	-1	-1
1362	1363	0,0321941	0,0005924	0,0005924	0	-1	-1
1363	1364	0,0153288	0,0005924	0,0005924	0	-1	-1
1364	1365	0,0145596	0,0005924	0,0005924	0	-1	-1
1365	1366	0,0123032	0,0005924	0,0005924	4,17298E+11	2,36469E+12	2,88525E-14
1274	1367	0,0134956	0,0009404	0,0009404	0	-1	-1
1367	1368	0,0121503	0,0009404	0,0009404	0	-1	-1
1368	1369	0,0096926	0,0007464	0,0007464	2,16017E+11	1,2241E+12	5,57366E-14
1368	1370	0,0236768	0,0007464	0,0007464	2,15401E+11	1,2206E+12	5,58962E-14
1244	1371	0,0243993	0,0017088	0,0017088	0	-1	-1
1371	1372	0,0144369	0,0017088	0,0017088	0	-1	-1
1372	1373	0,0137321	0,0017088	0,0017088	18107723339	1,0261E+11	6,64913E-13
1060	1374	0,0132022	0,0031052	0,0031052	3035674807	17202157241	3,96619E-12
1060	1375	0,0239742	0,0031052	0,0031052	3034088841	17193170099	3,96826E-12
1	1376	0,0563352	0,0071091	0,0071091	0	-1	-1
1376	1377	0,0256901	0,0049292	0,0049292	0	-1	-1

1377	1378	0,0118794	0,0049292	0,0049292	760063152,5	4307024531	1,58409E-11
1376	1379	0,0322580	0,0049292	0,0049292	0	-1	-1
1379	1380	0,0201729	0,0039123	0,0039123	0	-1	-1
1380	1381	0,0251896	0,0027126	0,0027126	0	-1	-1
1381	1382	0,0067931	0,0027126	0,0027126	0	-1	-1
1382	1383	0,0111485	0,0021530	0,0021530	0	-1	-1
1383	1384	0,0222652	0,0021530	0,0021530	0	-1	-1
1384	1385	0,0267544	0,0017088	0,0017088	0	-1	-1
1385	1386	0,0080103	0,0011848	0,0011848	54281771551	3,07597E+11	2,21807E-13
1385	1387	0,0235193	0,0011848	0,0011848	0	-1	-1
1387	1388	0,0125462	0,0008215	0,0008215	0	-1	-1
1388	1389	0,0188413	0,0006520	0,0006520	0	-1	-1
1389	1390	0,0165585	0,0006520	0,0006520	0	-1	-1
1390	1391	0,0285655	0,0006520	0,0006520	3,19447E+11	1,8102E+12	3,76904E-14
1388	1392	0,0194880	0,0006520	0,0006520	0	-1	-1
1392	1393	0,0126754	0,0006520	0,0006520	3,21855E+11	1,82384E+12	3,74084E-14
1387	1394	0,0188204	0,0008215	0,0008215	0	-1	-1
1394	1395	0,0256891	0,0008215	0,0008215	0	-1	-1
1395	1396	0,0124408	0,0008215	0,0008215	1,60811E+11	9,11261E+11	7,4871E-14
1387	1397	0,0302760	0,0008215	0,0008215	0	-1	-1
1397	1398	0,0245093	0,0006520	0,0006520	0	-1	-1
1398	1399	0,0150857	0,0004521	0,0004521	9,59164E+11	5,43526E+12	1,25527E-14
1398	1400	0,0186170	0,0004521	0,0004521	0	-1	-1
1400	1401	0,0150995	0,0004521	0,0004521	0	-1	-1
1401	1402	0,0285047	0,0003588	0,0003588	0	-1	-1
1402	1403	0,0181219	0,0002848	0,0002848	3,72747E+12	2,11223E+13	3,23009E-15
1402	1404	0,0301576	0,0002848	0,0002848	0	-1	-1
1404	1405	0,0208147	0,0002848	0,0002848	0	-1	-1
1405	1406	0,0097410	0,0002848	0,0002848	0	-1	-1
1406	1407	0,0233235	0,0002848	0,0002848	0	-1	-1
1407	1408	0,0164574	0,0002261	0,0002261	7,09442E+12	4,02017E+13	1,69712E-15
1407	1409	0,0332992	0,0002261	0,0002261	7,00613E+12	3,97014E+13	1,7185E-15
1401	1410	0,0328264	0,0003588	0,0003588	0	-1	-1
1410	1411	0,0108480	0,0003588	0,0003588	1,87006E+12	1,0597E+13	6,43833E-15
1398	1412	0,0343216	0,0004521	0,0004521	0	-1	-1
1412	1413	0,0228047	0,0004521	0,0004521	0	-1	-1
1413	1414	0,0319915	0,0003588	0,0003588	0	-1	-1
1414	1415	0,0226082	0,0003588	0,0003588	1,8457E+12	1,0459E+13	6,5233E-15
1413	1416	0,0336173	0,0003588	0,0003588	0	-1	-1
1416	1417	0,0210749	0,0002848	0,0002848	0	-1	-1
1417	1418	0,0134495	0,0002848	0,0002848	3,65423E+12	2,07073E+13	3,29483E-15
1416	1419	0,0253561	0,0002848	0,0002848	0	-1	-1
1419	1420	0,0088355	0,0002848	0,0002848	3,65492E+12	2,07112E+13	3,29421E-15
1397	1421	0,0270623	0,0006520	0,0006520	0	-1	-1
1421	1422	0,0297470	0,0006520	0,0006520	0	-1	-1
1422	1423	0,0160059	0,0005175	0,0005175	6,34792E+11	3,59715E+12	1,8967E-14
1422	1424	0,0346660	0,0005175	0,0005175	0	-1	-1
1424	1425	0,0138953	0,0005175	0,0005175	6,2858E+11	3,56195E+12	1,91544E-14
1385	1426	0,0420204	0,0011848	0,0011848	0	-1	-1
1426	1427	0,0385854	0,0011848	0,0011848	0	-1	-1
1427	1428	0,0228506	0,0009404	0,0009404	0	-1	-1
1428	1429	0,0144077	0,0007464	0,0007464	2,13675E+11	1,21083E+12	5,63476E-14
1428	1430	0,0165483	0,0007464	0,0007464	0	-1	-1

1430	1431	0,0272979	0,0007464	0,0007464	0	-1	-1
1431	1432	0,0281466	0,0007464	0,0007464	2,11136E+11	1,19643E+12	5,70253E-14
1427	1433	0,0240686	0,0009404	0,0009404	0	-1	-1
1433	1434	0,0218098	0,0006520	0,0006520	3,1975E+11	1,81192E+12	3,76546E-14
1433	1435	0,0348788	0,0006520	0,0006520	0	-1	-1
1435	1436	0,0209124	0,0006520	0,0006520	0	-1	-1
1436	1437	0,0302497	0,0006520	0,0006520	0	-1	-1
1437	1438	0,0192752	0,0005175	0,0005175	0	-1	-1
1438	1439	0,0107152	0,0005175	0,0005175	0	-1	-1
1439	1440	0,0048716	0,0005175	0,0005175	6,23121E+11	3,53102E+12	1,93222E-14
1437	1441	0,0213045	0,0005175	0,0005175	0	-1	-1
1441	1442	0,0233327	0,0004108	0,0004108	1,2402E+12	7,02778E+12	9,70819E-15
1441	1443	0,0281462	0,0004108	0,0004108	1,23788E+12	7,01467E+12	9,72634E-15
1433	1444	0,0363448	0,0006520	0,0006520	0	-1	-1
1444	1445	0,0111850	0,0004521	0,0004521	0	-1	-1
1445	1446	0,0141428	0,0004521	0,0004521	9,47651E+11	5,37002E+12	1,27052E-14
1444	1447	0,0200932	0,0004521	0,0004521	0	-1	-1
1447	1448	0,0242139	0,0003588	0,0003588	1,87874E+12	1,06462E+13	6,40859E-15
1447	1449	0,0364635	0,0003588	0,0003588	0	-1	-1
1449	1450	0,0164030	0,0002848	0,0002848	0	-1	-1
1450	1451	0,0081740	0,0002261	0,0002261	7,36341E+12	4,1726E+13	1,63512E-15
1450	1452	0,0361700	0,0002261	0,0002261	7,21665E+12	4,08944E+13	1,66837E-15
1449	1453	0,0260206	0,0002848	0,0002848	0	-1	-1
1453	1454	0,0169724	0,0002261	0,0002261	7,27727E+12	4,12379E+13	1,65448E-15
1453	1455	0,0332882	0,0002261	0,0002261	0	-1	-1
1455	1456	0,0121149	0,0002261	0,0002261	0	-1	-1
1456	1457	0,0060079	0,0002261	0,0002261	7,09674E+12	4,02149E+13	1,69656E-15
1444	1458	0,0212990	0,0004521	0,0004521	9,4897E+11	5,3775E+12	1,26875E-14
1384	1459	0,0390297	0,0017088	0,0017088	0	-1	-1
1459	1460	0,0167698	0,0013563	0,0013563	0	-1	-1
1460	1461	0,0099298	0,0013563	0,0013563	0	-1	-1
1461	1462	0,0244024	0,0013563	0,0013563	0	-1	-1
1462	1463	0,0126441	0,0007932	0,0007932	1,79457E+11	1,01692E+12	6,70917E-14
1462	1464	0,0215719	0,0007932	0,0007932	1,79148E+11	1,01517E+12	6,72073E-14
1462	1465	0,0278323	0,0007932	0,0007932	0	-1	-1
1465	1466	0,0148549	0,0006295	0,0006295	0	-1	-1
1466	1467	0,0075968	0,0006295	0,0006295	3,55907E+11	2,0168E+12	3,38293E-14
1465	1468	0,0171874	0,0006295	0,0006295	3,56365E+11	2,0194E+12	3,37857E-14
1462	1469	0,0294275	0,0007932	0,0007932	0	-1	-1
1469	1470	0,0249369	0,0007932	0,0007932	0	-1	-1
1470	1471	0,0129510	0,0007932	0,0007932	1,77566E+11	1,00621E+12	6,78061E-14
1462	1472	0,0306028	0,0007932	0,0007932	0	-1	-1
1472	1473	0,0204262	0,0007932	0,0007932	0	-1	-1
1473	1474	0,0223033	0,0006295	0,0006295	0	-1	-1
1474	1475	0,0268644	0,0006295	0,0006295	0	-1	-1
1475	1476	0,0198085	0,0006295	0,0006295	3,50248E+11	1,98474E+12	3,43758E-14
1473	1477	0,0273982	0,0006295	0,0006295	0	-1	-1
1477	1478	0,0143785	0,0004997	0,0004997	0	-1	-1
1478	1479	0,0150137	0,0004997	0,0004997	0	-1	-1
1479	1480	0,0216506	0,0003966	0,0003966	1,3906E+12	7,88005E+12	8,65821E-15
1479	1481	0,0344763	0,0003966	0,0003966	0	-1	-1
1481	1482	0,0203157	0,0003966	0,0003966	0	-1	-1
1482	1483	0,0043426	0,0003148	0,0003148	2,73846E+12	1,55179E+13	4,39665E-15

1482	1484	0,0159660	0,0003148	0,0003148	0	-1	-1
1484	1485	0,0267519	0,0003148	0,0003148	2,68495E+12	1,52147E+13	4,48427E-15
1477	1486	0,0152571	0,0004997	0,0004997	0	-1	-1
1486	1487	0,0126619	0,0004997	0,0004997	0	-1	-1
1487	1488	0,0110517	0,0003966	0,0003966	1,39711E+12	7,91695E+12	8,61785E-15
1487	1489	0,0254944	0,0003966	0,0003966	0	-1	-1
1489	1490	0,0170603	0,0003148	0,0003148	0	-1	-1
1490	1491	0,0124733	0,0002183	0,0002183	8,18809E+12	4,63992E+13	1,47044E-15
1490	1492	0,0160084	0,0002183	0,0002183	8,16676E+12	4,62783E+13	1,47428E-15
1490	1493	0,0169502	0,0002183	0,0002183	8,16108E+12	4,62461E+13	1,4753E-15
1489	1494	0,0191310	0,0003148	0,0003148	0	-1	-1
1494	1495	0,0084818	0,0002498	0,0002498	0	-1	-1
1495	1496	0,0435170	0,0001983	0,0001983	1,05614E+13	5,98477E+13	1,14001E-15
1495	1497	0,0436432	0,0001983	0,0001983	1,05602E+13	5,98414E+13	1,14013E-15
1494	1498	0,0291328	0,0002498	0,0002498	5,40076E+12	3,06043E+13	2,22933E-15
1459	1499	0,0266462	0,0013563	0,0013563	0	-1	-1
1499	1500	0,0137757	0,0010765	0,0010765	0	-1	-1
1500	1501	0,0078965	0,0010765	0,0010765	0	-1	-1
1501	1502	0,0202458	0,0010765	0,0010765	71728247313	4,0646E+11	1,67857E-13
1499	1503	0,0165521	0,0010765	0,0010765	71986788064	4,07925E+11	1,67254E-13
1382	1504	0,0116904	0,0021530	0,0021530	0	-1	-1
1504	1505	0,0093055	0,0021530	0,0021530	0	-1	-1
1505	1506	0,0131998	0,0021530	0,0021530	9077209335	51437519568	1,32641E-12
1380	1507	0,0280784	0,0027126	0,0027126	0	-1	-1
1507	1508	0,0416354	0,0027126	0,0027126	0	-1	-1
1508	1509	0,0235676	0,0021530	0,0021530	0	-1	-1
1509	1510	0,0171918	0,0021530	0,0021530	0	-1	-1
1510	1511	0,0199223	0,0021530	0,0021530	0	-1	-1
1511	1512	0,0091111	0,0021530	0,0021530	9035455798	51200916188	1,33254E-12
1508	1513	0,0261046	0,0021530	0,0021530	0	-1	-1
1513	1514	0,0110319	0,0021530	0,0021530	9056258703	51318799314	1,32947E-12
1380	1515	0,0297341	0,0027126	0,0027126	0	-1	-1
1515	1516	0,0316344	0,0027126	0,0027126	0	-1	-1
1516	1517	0,0255036	0,0021530	0,0021530	0	-1	-1
1517	1518	0,0079161	0,0021530	0,0021530	0	-1	-1
1518	1519	0,0312291	0,0021530	0,0021530	0	-1	-1
1519	1520	0,0287973	0,0021530	0,0021530	9024607433	51139442118	1,33414E-12
1516	1521	0,0292681	0,0021530	0,0021530	0	-1	-1
1521	1522	0,0135885	0,0017088	0,0017088	0	-1	-1
1522	1523	0,0326719	0,0017088	0,0017088	0	-1	-1
1523	1524	0,0126051	0,0010765	0,0010765	0	-1	-1
1524	1525	0,0061841	0,0008544	0,0008544	1,44038E+11	8,16215E+11	8,35895E-14
1524	1526	0,0414462	0,0008544	0,0008544	0	-1	-1
1526	1527	0,0281790	0,0006782	0,0006782	0	-1	-1
1527	1528	0,0190842	0,0004272	0,0004272	0	-1	-1
1528	1529	0,0088771	0,0004272	0,0004272	0	-1	-1
1529	1530	0,0097863	0,0004272	0,0004272	0	-1	-1
1530	1531	0,0284975	0,0004272	0,0004272	0	-1	-1
1531	1532	0,0153039	0,0003391	0,0003391	0	-1	-1
1532	1533	0,0229042	0,0003391	0,0003391	0	-1	-1
1533	1534	0,0362066	0,0003391	0,0003391	0	-1	-1
1534	1535	0,0306380	0,0002691	0,0002691	0	-1	-1
1535	1536	0,0238988	0,0002691	0,0002691	0	-1	-1

1536	1537	0,0094850	0,0002691	0,0002691	0	-1	-1
1537	1538	0,0136947	0,0002691	0,0002691	0	-1	-1
1538	1539	0,0163439	0,0002691	0,0002691	4,04263E+12	2,29082E+13	2,97828E-15
1534	1540	0,0310183	0,0002691	0,0002691	4,20712E+12	2,38404E+13	2,86183E-15
1531	1541	0,0243291	0,0003391	0,0003391	0	-1	-1
1541	1542	0,0157365	0,0003391	0,0003391	2,1796E+12	1,2351E+13	5,52399E-15
1527	1543	0,0231444	0,0004272	0,0004272	1,12825E+12	6,39344E+12	1,06714E-14
1527	1544	0,0256986	0,0004272	0,0004272	1,1272E+12	6,38749E+12	1,06814E-14
1527	1545	0,0273981	0,0004272	0,0004272	0	-1	-1
1545	1546	0,0284466	0,0004272	0,0004272	0	-1	-1
1546	1547	0,0314487	0,0004272	0,0004272	0	-1	-1
1547	1548	0,0214568	0,0004272	0,0004272	0	-1	-1
1548	1549	0,0118290	0,0002691	0,0002691	4,34143E+12	2,46015E+13	2,77329E-15
1548	1550	0,0150458	0,0002691	0,0002691	0	-1	-1
1550	1551	0,0121908	0,0002691	0,0002691	4,30123E+12	2,43736E+13	2,79921E-15
1548	1552	0,0178899	0,0002691	0,0002691	4,32562E+12	2,45118E+13	2,78343E-15
1548	1553	0,0206742	0,0002691	0,0002691	0	-1	-1
1553	1554	0,0289705	0,0002136	0,0002136	8,44623E+12	4,7862E+13	1,4255E-15
1553	1555	0,0331837	0,0002136	0,0002136	8,41853E+12	4,7705E+13	1,43019E-15
1526	1556	0,0402181	0,0006782	0,0006782	2,83662E+11	1,60742E+12	4,24451E-14
1523	1557	0,0163068	0,0010765	0,0010765	72060687955	4,08344E+11	1,67082E-13
1523	1558	0,0268332	0,0010765	0,0010765	0	-1	-1
1558	1559	0,0132989	0,0008544	0,0008544	0	-1	-1
1559	1560	0,0080602	0,0006782	0,0006782	0	-1	-1
1560	1561	0,0329403	0,0006782	0,0006782	2,84477E+11	1,61204E+12	4,23235E-14
1559	1562	0,0474421	0,0006782	0,0006782	0	-1	-1
1562	1563	0,0213163	0,0005383	0,0005383	0	-1	-1
1563	1564	0,0298934	0,0004272	0,0004272	0	-1	-1
1564	1565	0,0150996	0,0004272	0,0004272	1,1108E+12	6,29453E+12	1,08391E-14
1563	1566	0,0300925	0,0004272	0,0004272	1,11692E+12	6,32922E+12	1,07797E-14
1562	1567	0,0275665	0,0005383	0,0005383	0	-1	-1
1567	1568	0,0206549	0,0003732	0,0003732	0	-1	-1
1568	1569	0,0116257	0,0002962	0,0002962	3,33193E+12	1,88809E+13	3,61354E-15
1568	1570	0,0130361	0,0002962	0,0002962	3,32942E+12	1,88667E+13	3,61626E-15
1567	1571	0,0301541	0,0003732	0,0003732	0	-1	-1
1571	1572	0,0222606	0,0003732	0,0003732	0	-1	-1
1572	1573	0,0138411	0,0002962	0,0002962	3,28317E+12	1,86046E+13	3,66721E-15
1572	1574	0,0173617	0,0002962	0,0002962	0	-1	-1
1574	1575	0,0099981	0,0002054	0,0002054	0	-1	-1
1575	1576	0,0016123	0,0002054	0,0002054	0	-1	-1
1576	1577	0,0227128	0,0002054	0,0002054	0	-1	-1
1577	1578	0,0082602	0,0001630	0,0001630	1,89732E+13	1,07515E+14	6,34581E-16
1577	1579	0,0170500	0,0001630	0,0001630	1,88029E+13	1,0655E+14	6,40332E-16
1574	1580	0,0257369	0,0002054	0,0002054	9,63274E+12	5,45855E+13	1,24991E-15
1574	1581	0,0318633	0,0002054	0,0002054	0	-1	-1
1581	1582	0,0347377	0,0002054	0,0002054	0	-1	-1
1582	1583	0,0309197	0,0002054	0,0002054	0	-1	-1
1583	1584	0,0119043	0,0001630	0,0001630	1,79302E+13	1,01605E+14	6,71495E-16
1583	1585	0,0190347	0,0001630	0,0001630	1,7792E+13	1,00821E+14	6,76712E-16
1567	1586	0,0390966	0,0003732	0,0003732	0	-1	-1
1586	1587	0,0136347	0,0002588	0,0002588	0	-1	-1
1587	1588	0,0199287	0,0002588	0,0002588	0	-1	-1
1588	1589	0,0109479	0,0002588	0,0002588	0	-1	-1

1589	1590	0,0193310	0,0002054	0,0002054	0	-1	-1
1590	1591	0,0118491	0,0002054	0,0002054	0	-1	-1
1591	1592	0,0239841	0,0001424	0,0001424	0	-1	-1
1592	1593	0,0145666	0,0001424	0,0001424	2,7121E+13	1,53686E+14	4,43938E-16
1591	1594	0,0286126	0,0001424	0,0001424	0	-1	-1
1594	1595	0,0177579	0,0001130	0,0001130	5,34142E+13	3,02681E+14	2,25409E-16
1594	1596	0,0184982	0,0001130	0,0001130	5,33521E+13	3,02329E+14	2,25672E-16
1591	1597	0,0368110	0,0001424	0,0001424	0	-1	-1
1597	1598	0,0226846	0,0001424	0,0001424	0	-1	-1
1598	1599	0,0184676	0,0001424	0,0001424	2,58092E+13	1,46252E+14	4,66504E-16
1589	1600	0,0233120	0,0002054	0,0002054	9,5286E+12	5,39954E+13	1,26357E-15
1586	1601	0,0182140	0,0002588	0,0002588	0	-1	-1
1601	1602	0,0130361	0,0001794	0,0001794	1,46306E+13	8,29065E+13	8,22939E-16
1601	1603	0,0155742	0,0001794	0,0001794	1,4597E+13	8,27166E+13	8,24829E-16
1601	1604	0,0170217	0,0001794	0,0001794	0	-1	-1
1604	1605	0,0226460	0,0001244	0,0001244	0	-1	-1
1605	1606	0,0099744	0,0000987	0,0000987	0	-1	-1
1606	1607	0,0100584	0,0000784	0,0000784	1,63235E+14	9,24996E+14	7,37593E-17
1606	1608	0,0123522	0,0000784	0,0000784	1,62402E+14	9,20279E+14	7,41374E-17
1605	1609	0,0131850	0,0000987	0,0000987	0	-1	-1
1609	1610	0,0072042	0,0000784	0,0000784	0	-1	-1
1610	1611	0,0191209	0,0000784	0,0000784	1,56407E+14	8,86304E+14	7,69793E-17
1609	1612	0,0135392	0,0000784	0,0000784	0	-1	-1
1612	1613	0,0201855	0,0000622	0,0000622	0	-1	-1
1613	1614	0,0205214	0,0000622	0,0000622	2,84868E+14	1,61425E+15	4,22654E-17
1612	1615	0,0206117	0,0000622	0,0000622	0	-1	-1
1615	1616	0,0104034	0,0000494	0,0000494	5,82517E+14	3,30093E+15	2,06691E-17
1615	1617	0,0136316	0,0000494	0,0000494	5,75078E+14	3,25877E+15	2,09364E-17
1604	1618	0,0257723	0,0001244	0,0001244	0	-1	-1
1618	1619	0,0188738	0,0001244	0,0001244	0	-1	-1
1619	1620	0,0200814	0,0001244	0,0001244	4,00343E+13	2,26861E+14	3,00744E-16
1604	1621	0,0267982	0,0001244	0,0001244	0	-1	-1
1621	1622	0,0076329	0,0000863	0,0000863	1,24719E+14	7,0674E+14	9,65377E-17
1621	1623	0,0183063	0,0000863	0,0000863	1,22079E+14	6,91783E+14	9,86249E-17
1621	1624	0,0201726	0,0000863	0,0000863	0	-1	-1
1624	1625	0,0225988	0,0000685	0,0000685	2,29154E+14	1,29854E+15	5,25414E-17
1624	1626	0,0259826	0,0000685	0,0000685	2,27045E+14	1,28659E+15	5,30294E-17
1586	1627	0,0216538	0,0002588	0,0002588	4,92375E+12	2,79013E+13	2,4453E-15
1558	1628	0,0290149	0,0008544	0,0008544	0	-1	-1
1628	1629	0,0355612	0,0008544	0,0008544	0	-1	-1
1629	1630	0,0175004	0,0008544	0,0008544	1,41799E+11	8,03526E+11	8,49095E-14
1523	1631	0,0273869	0,0010765	0,0010765	71947754996	4,07704E+11	1,67345E-13
1521	1632	0,0222476	0,0017088	0,0017088	0	-1	-1
1632	1633	0,0133600	0,0013563	0,0013563	0	-1	-1
1633	1634	0,0214056	0,0013563	0,0013563	0	-1	-1
1634	1635	0,0240354	0,0013563	0,0013563	35952695305	2,03732E+11	3,34886E-13
1632	1636	0,0193421	0,0013563	0,0013563	36112301984	2,04636E+11	3,33406E-13
1379	1637	0,0274757	0,0039123	0,0039123	1518767260	8606347807	7,92753E-12
1376	1638	0,0469950	0,0049292	0,0049292	0	-1	-1
1638	1639	0,0243068	0,0034177	0,0034177	0	-1	-1
1639	1640	0,0147149	0,0034177	0,0034177	0	-1	-1
1640	1641	0,0266720	0,0034177	0,0034177	0	-1	-1
1641	1642	0,0153989	0,0023697	0,0023697	6812144708	38602153347	1,76744E-12

1641	1643	0,0155736	0,0023697	0,0023697	0	-1	-1
1643	1644	0,0130117	0,0023697	0,0023697	0	-1	-1
1644	1645	0,0183741	0,0023697	0,0023697	0	-1	-1
1645	1646	0,0182479	0,0023697	0,0023697	6790523690	38479634241	1,77307E-12
1641	1647	0,0168385	0,0023697	0,0023697	0	-1	-1
1647	1648	0,0267590	0,0023697	0,0023697	0	-1	-1
1648	1649	0,0121262	0,0023697	0,0023697	0	-1	-1
1649	1650	0,0177546	0,0023697	0,0023697	0	-1	-1
1650	1651	0,0115824	0,0016431	0,0016431	0	-1	-1
1651	1652	0,0214323	0,0016431	0,0016431	0	-1	-1
1652	1653	0,0123291	0,0016431	0,0016431	20275636202	1,14895E+11	5,93819E-13
1650	1654	0,0554047	0,0016431	0,0016431	0	-1	-1
1654	1655	0,0177573	0,0013041	0,0013041	0	-1	-1
1655	1656	0,0261202	0,0013041	0,0013041	40305821712	2,284E+11	2,98718E-13
1654	1657	0,0337041	0,0013041	0,0013041	0	-1	-1
1657	1658	0,0074196	0,0009042	0,0009042	0	-1	-1
1658	1659	0,0140392	0,0009042	0,0009042	1,20622E+11	6,83527E+11	9,98161E-14
1657	1660	0,0163736	0,0009042	0,0009042	1,20727E+11	6,84118E+11	9,973E-14
1657	1661	0,0179028	0,0009042	0,0009042	1,20695E+11	6,8394E+11	9,97559E-14
1650	1662	0,0632341	0,0016431	0,0016431	0	-1	-1
1662	1663	0,0270212	0,0016431	0,0016431	0	-1	-1
1663	1664	0,0139216	0,0013041	0,0013041	40316682776	2,28461E+11	2,98637E-13
1663	1665	0,0277155	0,0013041	0,0013041	40251400487	2,28091E+11	2,99122E-13
1638	1666	0,0264248	0,0034177	0,0034177	0	-1	-1
1666	1667	0,0368416	0,0027126	0,0027126	0	-1	-1
1667	1668	0,0180042	0,0021530	0,0021530	0	-1	-1
1668	1669	0,0133097	0,0013563	0,0013563	36255900640	2,0545E+11	3,32086E-13
1668	1670	0,0146874	0,0013563	0,0013563	36250328100	2,05419E+11	3,32137E-13
1668	1671	0,0152917	0,0013563	0,0013563	36247883566	2,05405E+11	3,32159E-13
1668	1672	0,0252345	0,0013563	0,0013563	0	-1	-1
1672	1673	0,0116203	0,0010765	0,0010765	0	-1	-1
1673	1674	0,0173739	0,0010765	0,0010765	0	-1	-1
1674	1675	0,0110416	0,0010765	0,0010765	0	-1	-1
1675	1676	0,0181798	0,0007464	0,0007464	2,1522E+11	1,21958E+12	5,59431E-14
1675	1677	0,0204212	0,0007464	0,0007464	0	-1	-1
1677	1678	0,0110899	0,0005924	0,0005924	0	-1	-1
1678	1679	0,0257815	0,0005924	0,0005924	4,26145E+11	2,41482E+12	2,82534E-14
1677	1680	0,0135633	0,0005924	0,0005924	0	-1	-1
1680	1681	0,0224468	0,0005924	0,0005924	0	-1	-1
1681	1682	0,0136634	0,0004702	0,0004702	0	-1	-1
1682	1683	0,0150384	0,0002962	0,0002962	0	-1	-1
1683	1684	0,0102515	0,0002962	0,0002962	3,34966E+12	1,89814E+13	3,59442E-15
1682	1685	0,0238450	0,0002962	0,0002962	0	-1	-1
1685	1686	0,0145848	0,0002962	0,0002962	0	-1	-1
1686	1687	0,0152519	0,0002962	0,0002962	0	-1	-1
1687	1688	0,0087272	0,0002962	0,0002962	3,28366E+12	1,86074E+13	3,66666E-15
1682	1689	0,0263580	0,0002962	0,0002962	0	-1	-1
1689	1690	0,0181361	0,0002962	0,0002962	3,31551E+12	1,87879E+13	3,63144E-15
1682	1691	0,0268282	0,0002962	0,0002962	0	-1	-1
1691	1692	0,0227599	0,0002962	0,0002962	0	-1	-1
1692	1693	0,0201661	0,0002962	0,0002962	3,2706E+12	1,85334E+13	3,6813E-15
1681	1694	0,0201563	0,0004702	0,0004702	8,46837E+11	4,79875E+12	1,42177E-14
1675	1695	0,0210893	0,0007464	0,0007464	2,15092E+11	1,21885E+12	5,59764E-14

1672	1696	0,0265437	0,0010765	0,0010765	0	-1	-1
1696	1697	0,0289036	0,0008544	0,0008544	0	-1	-1
1697	1698	0,0146852	0,0008544	0,0008544	0	-1	-1
1698	1699	0,0092554	0,0008544	0,0008544	0	-1	-1
1699	1700	0,0184340	0,0008544	0,0008544	0	-1	-1
1700	1701	0,0098496	0,0008544	0,0008544	0	-1	-1
1701	1702	0,0151749	0,0006782	0,0006782	0	-1	-1
1702	1703	0,0026295	0,0005383	0,0005383	0	-1	-1
1703	1704	0,0107393	0,0005383	0,0005383	5,64679E+11	3,19985E+12	2,1322E-14
1702	1705	0,0334966	0,0005383	0,0005383	5,61397E+11	3,18125E+12	2,14466E-14
1701	1706	0,0210111	0,0006782	0,0006782	0	-1	-1
1706	1707	0,0152938	0,0005383	0,0005383	5,6361E+11	3,19379E+12	2,13624E-14
1706	1708	0,0238337	0,0005383	0,0005383	5,62217E+11	3,1859E+12	2,14153E-14
1696	1709	0,0399240	0,0008544	0,0008544	0	-1	-1
1709	1710	0,0085650	0,0008544	0,0008544	0	-1	-1
1710	1711	0,0229049	0,0008544	0,0008544	0	-1	-1
1711	1712	0,0360864	0,0005924	0,0005924	0	-1	-1
1712	1713	0,0112712	0,0004108	0,0004108	0	-1	-1
1713	1714	0,0193267	0,0003260	0,0003260	2,50589E+12	1,42001E+13	4,8047E-15
1713	1715	0,0225783	0,0003260	0,0003260	0	-1	-1
1715	1716	0,0237779	0,0003260	0,0003260	2,47314E+12	1,40145E+13	4,86833E-15
1712	1717	0,0157944	0,0004108	0,0004108	1,26248E+12	7,15405E+12	9,53685E-15
1712	1718	0,0243169	0,0004108	0,0004108	0	-1	-1
1718	1719	0,0332044	0,0004108	0,0004108	0	-1	-1
1719	1720	0,0171379	0,0004108	0,0004108	0	-1	-1
1720	1721	0,0086181	0,0004108	0,0004108	1,23003E+12	6,97018E+12	9,78842E-15
1711	1722	0,0418507	0,0005924	0,0005924	0	-1	-1
1722	1723	0,0230379	0,0005924	0,0005924	4,20157E+11	2,38089E+12	2,86561E-14
1711	1724	0,0433872	0,0005924	0,0005924	0	-1	-1
1724	1725	0,0149647	0,0004108	0,0004108	0	-1	-1
1725	1726	0,0219176	0,0004108	0,0004108	1,24991E+12	7,0828E+12	9,63278E-15
1724	1727	0,0231172	0,0004108	0,0004108	0	-1	-1
1727	1728	0,0095897	0,0003260	0,0003260	2,50143E+12	1,41748E+13	4,81327E-15
1727	1729	0,0171652	0,0003260	0,0003260	0	-1	-1
1729	1730	0,0052809	0,0003260	0,0003260	2,48585E+12	1,40865E+13	4,84343E-15
1724	1731	0,0272120	0,0004108	0,0004108	1,25456E+12	7,10915E+12	9,59708E-15
1667	1732	0,0355956	0,0021530	0,0021530	0	-1	-1
1732	1733	0,0077532	0,0012591	0,0012591	45288912130	2,56637E+11	2,6585E-13
1732	1734	0,0241075	0,0012591	0,0012591	0	-1	-1
1734	1735	0,0186582	0,0012591	0,0012591	0	-1	-1
1735	1736	0,0101881	0,0009993	0,0009993	0	-1	-1
1736	1737	0,0130503	0,0009993	0,0009993	89877500141	5,09306E+11	1,33961E-13
1735	1738	0,0403174	0,0009993	0,0009993	0	-1	-1
1738	1739	0,0255921	0,0007932	0,0007932	0	-1	-1
1739	1740	0,0329720	0,0007932	0,0007932	1,77261E+11	1,00448E+12	6,79229E-14
1738	1741	0,0271703	0,0007932	0,0007932	1,78347E+11	1,01063E+12	6,75094E-14
1732	1742	0,0259112	0,0012591	0,0012591	0	-1	-1
1742	1743	0,0185415	0,0008730	0,0008730	0	-1	-1
1743	1744	0,0171359	0,0008730	0,0008730	1,34729E+11	7,63466E+11	8,93649E-14
1742	1745	0,0289903	0,0008730	0,0008730	0	-1	-1
1745	1746	0,0124791	0,0008730	0,0008730	0	-1	-1
1746	1747	0,0100129	0,0008730	0,0008730	1,34357E+11	7,61355E+11	8,96126E-14
1742	1748	0,0324464	0,0008730	0,0008730	0	-1	-1

1748	1749	0,0366523	0,0008730	0,0008730	0	-1	-1
1749	1750	0,0170316	0,0006929	0,0006929	2,66872E+11	1,51227E+12	4,51155E-14
1749	1751	0,0236609	0,0006929	0,0006929	0	-1	-1
1751	1752	0,0098608	0,0004804	0,0004804	7,96901E+11	4,51578E+12	1,51086E-14
1751	1753	0,0124124	0,0004804	0,0004804	0	-1	-1
1753	1754	0,0081997	0,0004804	0,0004804	7,94139E+11	4,50012E+12	1,51612E-14
1751	1755	0,0129575	0,0004804	0,0004804	7,96106E+11	4,51127E+12	1,51237E-14
1732	1756	0,0321569	0,0012591	0,0012591	0	-1	-1
1756	1757	0,0426492	0,0012591	0,0012591	0	-1	-1
1757	1758	0,0186387	0,0007363	0,0007363	0	-1	-1
1758	1759	0,0181404	0,0007363	0,0007363	2,22906E+11	1,26313E+12	5,40141E-14
1757	1760	0,0270027	0,0007363	0,0007363	0	-1	-1
1760	1761	0,0101109	0,0007363	0,0007363	0	-1	-1
1761	1762	0,0142889	0,0007363	0,0007363	0	-1	-1
1762	1763	0,0069977	0,0005844	0,0005844	4,43629E+11	2,5139E+12	2,714E-14
1762	1764	0,0219826	0,0005844	0,0005844	4,4187E+11	2,50393E+12	2,7248E-14
1757	1765	0,0291899	0,0007363	0,0007363	0	-1	-1
1765	1766	0,0107816	0,0005844	0,0005844	4,45253E+11	2,5231E+12	2,70409E-14
1765	1767	0,0264741	0,0005844	0,0005844	0	-1	-1
1767	1768	0,0185513	0,0004639	0,0004639	0	-1	-1
1768	1769	0,0120755	0,0003682	0,0003682	1,75368E+12	9,93752E+12	6,8656E-15
1768	1770	0,0189140	0,0003682	0,0003682	0	-1	-1
1770	1771	0,0132871	0,0002922	0,0002922	3,47222E+12	1,96759E+13	3,46754E-15
1770	1772	0,0160574	0,0002922	0,0002922	0	-1	-1
1772	1773	0,0316531	0,0002922	0,0002922	3,4076E+12	1,93097E+13	3,5333E-15
1767	1774	0,0302137	0,0004639	0,0004639	8,7789E+11	4,97471E+12	1,37148E-14
1757	1775	0,0303188	0,0007363	0,0007363	0	-1	-1
1775	1776	0,0154060	0,0004639	0,0004639	8,88271E+11	5,03354E+12	1,35545E-14
1775	1777	0,0168577	0,0004639	0,0004639	8,87842E+11	5,03111E+12	1,3561E-14
1775	1778	0,0172012	0,0004639	0,0004639	8,87741E+11	5,03053E+12	1,35626E-14
1775	1779	0,0186996	0,0004639	0,0004639	0	-1	-1
1779	1780	0,0109145	0,0004639	0,0004639	0	-1	-1
1780	1781	0,0272577	0,0004639	0,0004639	0	-1	-1
1781	1782	0,0163428	0,0003216	0,0003216	2,60712E+12	1,47737E+13	4,61814E-15
1781	1783	0,0195091	0,0003216	0,0003216	0	-1	-1
1783	1784	0,0220029	0,0003216	0,0003216	2,57492E+12	1,45912E+13	4,67589E-15
1781	1785	0,0316405	0,0003216	0,0003216	0	-1	-1
1785	1786	0,0154086	0,0002553	0,0002553	5,12543E+12	2,90441E+13	2,34908E-15
1785	1787	0,0222224	0,0002553	0,0002553	0	-1	-1
1787	1788	0,0181403	0,0002553	0,0002553	5,04499E+12	2,85883E+13	2,38654E-15
1757	1789	0,0308210	0,0007363	0,0007363	0	-1	-1
1789	1790	0,0187317	0,0005105	0,0005105	6,65776E+11	3,77273E+12	1,80843E-14
1789	1791	0,0282221	0,0005105	0,0005105	0	-1	-1
1791	1792	0,0036439	0,0004052	0,0004052	1,32588E+12	7,5133E+12	9,08083E-15
1791	1793	0,0311510	0,0004052	0,0004052	1,31191E+12	7,43417E+12	9,1775E-15
1789	1794	0,0292415	0,0005105	0,0005105	0	-1	-1
1794	1795	0,0218780	0,0003216	0,0003216	0	-1	-1
1795	1796	0,0157314	0,0002553	0,0002553	0	-1	-1
1796	1797	0,0210982	0,0002026	0,0002026	1,02338E+13	5,79913E+13	1,17651E-15
1796	1798	0,0306087	0,0002026	0,0002026	0	-1	-1
1798	1799	0,0168119	0,0002026	0,0002026	0	-1	-1
1799	1800	0,0086339	0,0002026	0,0002026	9,9498E+12	5,63822E+13	1,21008E-15
1795	1801	0,0197910	0,0002553	0,0002553	5,18949E+12	2,94071E+13	2,32009E-15

1794	1802	0,0263904	0,0003216	0,0003216	0	-1	-1
1802	1803	0,0192586	0,0003216	0,0003216	2,59623E+12	1,4712E+13	4,63752E-15
1794	1804	0,0269258	0,0003216	0,0003216	0	-1	-1
1804	1805	0,0193341	0,0003216	0,0003216	0	-1	-1
1805	1806	0,0220036	0,0003216	0,0003216	2,5673E+12	1,4548E+13	4,68978E-15
1794	1807	0,0289274	0,0003216	0,0003216	0	-1	-1
1807	1808	0,0109023	0,0003216	0,0003216	0	-1	-1
1808	1809	0,0166046	0,0003216	0,0003216	0	-1	-1
1809	1810	0,0175292	0,0003216	0,0003216	0	-1	-1
1810	1811	0,0120667	0,0003216	0,0003216	2,54457E+12	1,44192E+13	4,73167E-15
1732	1812	0,0434156	0,0012591	0,0012591	0	-1	-1
1812	1813	0,0201914	0,0008730	0,0008730	0	-1	-1
1813	1814	0,0053014	0,0006929	0,0006929	2,69302E+11	1,52604E+12	4,47085E-14
1813	1815	0,0083779	0,0006929	0,0006929	0	-1	-1
1815	1816	0,0300405	0,0006929	0,0006929	0	-1	-1
1816	1817	0,0168588	0,0005500	0,0005500	5,32147E+11	3,0155E+12	2,26254E-14
1816	1818	0,0385355	0,0005500	0,0005500	5,28904E+11	2,99712E+12	2,27642E-14
1812	1819	0,0240521	0,0008730	0,0008730	0	-1	-1
1819	1820	0,0076800	0,0008730	0,0008730	0	-1	-1
1820	1821	0,0286614	0,0008730	0,0008730	0	-1	-1
1821	1822	0,0371341	0,0008730	0,0008730	0	-1	-1
1822	1823	0,0066199	0,0005500	0,0005500	0	-1	-1
1823	1824	0,0220169	0,0004365	0,0004365	0	-1	-1
1824	1825	0,0160582	0,0004365	0,0004365	1,04755E+12	5,93611E+12	1,14936E-14
1823	1826	0,0220816	0,0004365	0,0004365	0	-1	-1
1826	1827	0,0154191	0,0003465	0,0003465	0	-1	-1
1827	1828	0,0328265	0,0003465	0,0003465	0	-1	-1
1828	1829	0,0279548	0,0003465	0,0003465	0	-1	-1
1829	1830	0,0128364	0,0003465	0,0003465	0	-1	-1
1830	1831	0,0131066	0,0003465	0,0003465	2,01011E+12	1,13906E+13	5,98976E-15
1826	1832	0,0530935	0,0003465	0,0003465	2,05671E+12	1,16547E+13	5,85403E-15
1822	1833	0,0164145	0,0005500	0,0005500	5,29487E+11	3,00042E+12	2,27391E-14
1822	1834	0,0191849	0,0005500	0,0005500	0	-1	-1
1834	1835	0,0153171	0,0005500	0,0005500	5,2678E+11	2,98509E+12	2,2856E-14
1822	1836	0,0207499	0,0005500	0,0005500	0	-1	-1
1836	1837	0,0129409	0,0005500	0,0005500	0	-1	-1
1837	1838	0,0031529	0,0005500	0,0005500	5,2643E+11	2,9831E+12	2,28712E-14
1812	1839	0,0309739	0,0008730	0,0008730	0	-1	-1
1839	1840	0,0204089	0,0008730	0,0008730	0	-1	-1
1840	1841	0,0067261	0,0006929	0,0006929	0	-1	-1
1841	1842	0,0143750	0,0005500	0,0005500	0	-1	-1
1842	1843	0,0198096	0,0005500	0,0005500	0	-1	-1
1843	1844	0,0202243	0,0005500	0,0005500	0	-1	-1
1844	1845	0,0150788	0,0005500	0,0005500	5,25096E+11	2,97554E+12	2,29293E-14
1841	1846	0,0225667	0,0005500	0,0005500	0	-1	-1
1846	1847	0,0288416	0,0005500	0,0005500	0	-1	-1
1847	1848	0,0141268	0,0004365	0,0004365	1,05028E+12	5,95157E+12	1,14637E-14
1847	1849	0,0173173	0,0004365	0,0004365	0	-1	-1
1849	1850	0,0180745	0,0004365	0,0004365	1,04226E+12	5,90613E+12	1,15519E-14
1840	1851	0,0324203	0,0006929	0,0006929	0	-1	-1
1851	1852	0,0158744	0,0006929	0,0006929	2,65278E+11	1,50324E+12	4,53865E-14
1666	1853	0,0423148	0,0027126	0,0027126	0	-1	-1
1853	1854	0,0346995	0,0027126	0,0027126	0	-1	-1

1854	1855	0,0235153	0,0021530	0,0021530	0	-1	-1
1855	1856	0,0132379	0,0017088	0,0017088	0	-1	-1
1856	1857	0,0483112	0,0017088	0,0017088	0	-1	-1
1857	1858	0,0326062	0,0017088	0,0017088	0	-1	-1
1858	1859	0,0199338	0,0017088	0,0017088	0	-1	-1
1859	1860	0,0116262	0,0017088	0,0017088	17905423890	1,01464E+11	6,72426E-13
1855	1861	0,0206203	0,0017088	0,0017088	0	-1	-1
1861	1862	0,0273920	0,0017088	0,0017088	0	-1	-1
1862	1863	0,0112930	0,0011848	0,0011848	0	-1	-1
1863	1864	0,0197663	0,0011848	0,0011848	53874743596	3,0529E+11	2,23483E-13
1862	1865	0,0121100	0,0011848	0,0011848	0	-1	-1
1865	1866	0,0190436	0,0011848	0,0011848	0	-1	-1
1866	1867	0,0132048	0,0011848	0,0011848	53782376557	3,04767E+11	2,23866E-13
1862	1868	0,0394567	0,0011848	0,0011848	0	-1	-1
1868	1869	0,0076879	0,0011848	0,0011848	0	-1	-1
1869	1870	0,0240831	0,0009404	0,0009404	1,07105E+11	6,06926E+11	1,12414E-13
1869	1871	0,0295571	0,0009404	0,0009404	1,07009E+11	6,06383E+11	1,12515E-13
1854	1872	0,0336057	0,0021530	0,0021530	9047183896	51267375413	1,33081E-12
1638	1873	0,0299941	0,0034177	0,0034177	0	-1	-1
1873	1874	0,0201300	0,0034177	0,0034177	0	-1	-1
1874	1875	0,0093976	0,0027126	0,0027126	4546634370	25764261430	2,64813E-12
1874	1876	0,0314144	0,0027126	0,0027126	0	-1	-1
1876	1877	0,0060891	0,0017088	0,0017088	18154499372	1,02875E+11	6,632E-13
1876	1878	0,0112832	0,0017088	0,0017088	18146161726	1,02828E+11	6,63505E-13
1876	1879	0,0152841	0,0017088	0,0017088	18139739538	1,02792E+11	6,6374E-13
1876	1880	0,0235249	0,0017088	0,0017088	0	-1	-1
1880	1881	0,0262785	0,0017088	0,0017088	0	-1	-1
1881	1882	0,0203205	0,0013563	0,0013563	36086463305	2,0449E+11	3,33645E-13
1881	1883	0,0257276	0,0013563	0,0013563	0	-1	-1
1883	1884	0,0121064	0,0010765	0,0010765	0	-1	-1
1884	1885	0,0220658	0,0010765	0,0010765	71780886602	4,06758E+11	1,67734E-13
1883	1886	0,0210311	0,0010765	0,0010765	71914826193	4,07517E+11	1,67421E-13
